# Roadmap on emerging concepts in the physical biology of bacterial biofilms: from surface sensing to community formation

**DOI:** 10.1088/1478-3975/abdc0e

**Published:** 2021-06-23

**Authors:** Gerard C L Wong, Jyot D Antani, Pushkar P Lele, Jing Chen, Beiyan Nan, Marco J Kühn, Alexandre Persat, Jean-Louis Bru, Nina Molin Høyland-Kroghsbo, Albert Siryaporn, Jacinta C Conrad, Francesco Carrara, Yutaka Yawata, Roman Stocker, Yves V Brun, Gregory B Whitfield, Calvin K Lee, Jaime de Anda, William C Schmidt, Ramin Golestanian, George A O’Toole, Kyle A Floyd, Fitnat H Yildiz, Shuai Yang, Fan Jin, Masanori Toyofuku, Leo Eberl, Nobuhiko Nomura, Lori A Zacharoff, Mohamed Y El-Naggar, Sibel Ebru Yalcin, Nikhil S Malvankar, Mauricio D Rojas-Andrade, Allon I Hochbaum, Jing Yan, Howard A Stone, Ned S Wingreen, Bonnie L Bassler, Yilin Wu, Haoran Xu, Knut Drescher, Jörn Dunkel

**Affiliations:** 1 Department of Bioengineering, University of California—Los Angeles, Los Angeles, California, CA 90095, United States of America; 2 Department of Chemistry and Biochemistry, University of California—Los Angeles, Los Angeles, California, CA 90095, United States of America; 3 California NanoSystems Institute, University of California—Los Angeles, Los Angeles, California, CA 90095, United States of America; 4 Artie McFerrin Department of Chemical Engineering, Texas A & M University, College Station, TX 77843, United States of America; 5 Department of Biological Sciences, Virginia Polytechnic Institute and State University, Blacksburg, VA24061, United States of America; 6 Department of Biology, Texas A & M University, College Station, Texas, TX 77845, United States of America; 7 Institute of Bioengineering and Global Health Institute, School of Life Sciences, École Polytechnique Fédérale de Lausanne, Lausanne, Switzerland; 8 Department of Molecular Biology & Biochemistry, University of California—Irvine, California, CA 92697, United States of America; 9 Department of Plant and Environmental Sciences, University of Copenhagen, DK-1871 Frederiksberg, Denmark; 10 Department of Physics & Astronomy, University of California—Irvine, California, CA 92697, United States of America; 11 William A Brookshire Department of Chemical and Biomolecular Engineering, University of Houston, Houston, Texas, TX 77204, United States of America; 12 Institute of Environmental Engineering, Department of Civil, Environmental and Geomatic Engineering, ETH Zurich, 8093 Zurich, Switzerland; 13 Faculty of Life and Environmental Sciences, University of Tsukuba, Tsukuba, Ibaraki 305-8572, Japan; 14 Microbiology Research Center for Sustainability, University of Tsukuba, 305-8572 Tsukuba, Japan; 15 University of Montreal, Faculty of Medicine, Montreal, Quebec, H3C 3J7, Canada; 16 Max Planck Institute for Dynamics and Self-Organization (MPIDS), D-37077 Göttingen, Germany; 17 Rudolf Peierls Centre for Theoretical Physics, University of Oxford, Oxford OX1 3PU, United Kingdom; 18 Department of Microbiology and Immunology, Geisel School of Medicine at Dartmouth, Hanover, NH 03755, United States of America; 19 Department of Microbiology and Environmental Toxicology, University of California—Santa Cruz, Santa Cruz, California, CA 95060, United States of America; 20 CAS Key Laboratory of Quantitative Engineering Biology, Shenzhen Institute of Synthetic Biology, Shenzhen Institutes of Advanced Technology, Chinese Academy of Sciences, Shenzhen 518055, People’s Republic of China; 21 Department of Plant and Microbial Biology, University of Zürich, 8008 Zürich, Switzerland; 22 Department of Physics and Astronomy, University of Southern California, Los Angeles, California, CA 90089, United States of America; 23 Department of Chemistry, University of Southern California, Los Angeles, California, CA 90089, United States of America; 24 Department of Biological Sciences, University of Southern California, Los Angeles, California, CA 90089, United States of America; 25 Department of Molecular Biophysics and Biochemistry, Yale University, New Haven, Connecticut, CT 06516, United States of America; 26 Microbial Sciences Institute, Yale University, New Haven, Connecticut, CT 06516, United States of America; 27 Department of Materials Science and Engineering, University of California—Irvine, Irvine, California CA 92697, United States of America; 28 Department of Chemistry, University of California—Irvine, Irvine, California, CA 92697, United States of America; 29 Department of Chemical and Biomolecular Engineering, University of California—Irvine, Irvine, California, CA 92697, United States of America; 30 Department of Molecular, Cellular and Developmental Biology, Yale University, New Haven, Connecticut, CT 06511, United States of America; 31 Department of Mechanical and Aerospace Engineering, Princeton University, Princeton, New Jersey, NJ 08544, United States of America; 32 Department of Molecular Biology, Princeton University, Princeton, New Jersey, NJ 08544, United States of America; 33 Lewis-Sigler Institute for Integrative Genomics, Princeton University, Princeton, New Jersey, NJ 08544, United States of America; 34 The Howard Hughes Medical Institute, Chevy Chase, Maryland MD 20815, United States of America; 35 Department of Physics and Shenzhen Research Institute, The Chinese University of Hong Kong, Shatin, New Territories, Hong Kong, People’s Republic of China; 36 Max Planck Institute for Terrestrial Microbiology, 35043 Marburg, Germany; 37 Department of Physics, Philipps-Universität Marburg, 35043 Marburg, Germany; 38 Department of Mathematics, Massachusetts Institute of Technology, Cambridge, Massachusetts, MA 02139-4307, United States of America

**Keywords:** biofilms, physiology of microbes, cellular organisation, adhesion, motility

## Abstract

Bacterial biofilms are communities of bacteria that exist as aggregates that can adhere to surfaces or be free-standing. This complex, social mode of cellular organization is fundamental to the physiology of microbes and often exhibits surprising behavior. Bacterial biofilms are more than the sum of their parts: single-cell behavior has a complex relation to collective community behavior, in a manner perhaps cognate to the complex relation between atomic physics and condensed matter physics. Biofilm microbiology is a relatively young field by biology standards, but it has already attracted intense attention from physicists. Sometimes, this attention takes the form of seeing biofilms as inspiration for new physics. In this roadmap, we highlight the work of those who have taken the opposite strategy: we highlight the work of physicists and physical scientists who use physics to engage fundamental concepts in bacterial biofilm microbiology, including adhesion, sensing, motility, signaling, memory, energy flow, community formation and cooperativity. These contributions are juxtaposed with microbiologists who have made recent important discoveries on bacterial biofilms using state-of-the-art physical methods. The contributions to this roadmap exemplify how well physics and biology can be combined to achieve a new synthesis, rather than just a division of labor.

## 1. Introduction

Bacterial biofilms are integrated communities of cells that adhere to surfaces and are fundamental to the ecology and biology of bacteria. Bacterial biofilm communities can be harmful, such as those that contribute to lethal airway infections in cystic fibrosis. However, bacterial communities can also be beneficial, and help train your immune system or digest your vegetables, as well as break down hydrocarbons in oil spills. Recent collaborative work between physicists and microbiologists has shown that bacteria employ surprisingly sophisticated physics and chemistry in order to organize these biofilm communities on a surface.

How does one get started in this multidisciplinary field? One of the most common questions from incoming graduate students is whether they have to master biology before doing biophysics. The answer is not a simple one. Adapting an idea from Karl Kraus may begin to answer this question: instead of being someone who masters a language, an artist is rather a servant of the word. Besides depth of inquiry, what unites the contributors in this multidisciplinary roadmap is a cognate sense of service to the field of bacterial biofilm microbiology. Rather than using microbiology as a mere context for new physics, each contributor from physics in this roadmap is interested in microbiology itself, and uses different aspects of physics to discover new microbiology. Their contributions are juxtaposed with those of well-known microbiologists who have made recent important discoveries on bacterial biofilms using state-of-the-art physical methods. Using these organizing principles for this roadmap, we hope it can live up to the onomastic promise of physical biology.

Bacteria have developed various strategies to move, sense, and organize in low Reynolds number environments; these often involve bacterial motility appendages such as flagella. Antani and Lele review the role of the flagellum in motility and mechanosensing: obstructions in the rotation of the flagellar motor will drive recruitment of additional stator units to the motor to increase torque. Kühn and Persat review the mechanics and dynamics of type IV pili (TFP), which are extension–retraction appendages often compared to grappling hooks. In particular, they examine how TFP are coordinated by considering them from the perspective of non-equilibrium systems. Chen and Nan review ‘gliding’ motility, where bacteria do not use appendage technology at all for motility, and employ force-generating complexes along helical tracks instead. Bru, Høyland-Kroghsbo and Siryaporn review how stress responses can redirect movement of bacterial populations and ultimately control bacterial spatial organization, via quorum sensing (QS) and stress signals.

The roadmap also contains sections on how bacteria adapt their existence to complex environments. Conrad explores bacterial mechanisms for controlling adhesion on real, heterogeneous interfaces, both solid and liquid, including for example oil droplets, which are particularly important for mitigating oil spills. Marine microbial environments are often characterized by heterogeneous and transient nutrient fluctuations, which can lead to interesting bacterial ecologies in different environmental niches. Carrara, Yawata and Stocker describe how bacteria solve these problems by gene expression and energetic investments.

The first step in the formation of a bacterial biofilm is contact with the surface on which the community will eventually form, raising the intriguing question: ‘how does a microbe know it is on a surface?’ Intracellular second messengers such as cyclic-AMP (cAMP) and cdiGMP play key roles in this process, and have emerged as a kind of master regulator of bacterial behavior. Brun reviews how TFP are used to surface sense, using labeling and visualization of pili dynamics in live cells. Lee, de Anda, Schmidt, Golestanian, O’Toole and Wong review the signal processing of surface sensing and how it is propagated from mother cell to daughter cell via a kind of multigenerational memory. cdiGMP signaling and downstream biosynthesis of the exopolysaccharide biofilm matrix are pivotal events in bacterial community development. Floyd and Yildiz review the consequences of cdiGMP signaling in *Vibrio cholerae* using an elegant method based on an mRNA riboswitch-based biosensor to determine changes in cdiGMP, and on visualization of pili in live cells. ‘What I cannot create, I do not understand’ was found written on Richard Feynman’s blackboard at the time of his death in 1988. In this spirit, Yang and Jin take a completely different approach to surface sensing based on synthetic biology: they show how we can reprogram bacterial surface sensing behavior using the chemical language of second messengers via optogenetic control of bacterial cdiGMP production.

All bacteria have to solve their energy problems in order to survive. Electron transfer couples the oxidation of electron donors to the reduction of electron acceptors, and constitutes the basis of bacterial respiration. However, bacteria are not limited to electron donors (such as organic molecules in growth media) or electron acceptors (such as oxygen) that exist in solution. They can solve their ‘life or death’ electron transfer problems by coupling directly to a solid surface via extracellular electron transfer (EET), a process that allows metal-reducing and oxidizing bacteria to catalyze generation of electricity and waste degradation. There has been great recent progress in EET, specifically in understanding bacterial nanowires, which were previously thought to be composed of protein-based pilin units: the situation is considerably more complex and diverse. Zacharoff and El-Naggar show that in *Shewanella*, bacterial nanowires take the form of membrane extensions studded with cytochromes. Yalcin and Malvankar show that in *Geobacter*, the nanowires that provide a continuous path for electron flow are polymerized six-heme cytochrome OmcS.

What happens when bacterial communities become progressively more crowded? Ideas about QS have now spread well beyond microbiology. Toyofuku, Eberl and Nomura offer a new perspective. QS signals are often amphiphilic molecules. It turns out that bacteria can use membrane vesicles (MV) rather than solvated signal molecules to mediate a kind of quantized QS signaling. Yan, Stone, Wingreen and Bassler developed methods to image living biofilms with single-cell resolution, and show how *V. cholerae* grew from the founder cell to clusters of different morphologies to biofilms of ~10 000 cells. Using new quantitative imaging techniques, Rojas-Andrade and Hochbaum map out bacterial metabolism in communities, with heterogeneity that fluctuates in space and time. In the review from Wu and Xu, we come full circle, and examine motility, now in the form of self-organized synchronized collective motion of strongly interacting bacteria. In a forward looking review, Drescher and Dunkel examine how data science and machine learning may be used to help formulate the next generation of models for understanding key mechanisms and discovering general principles for biofilm formation.

The excellent individual roadmap sections collected here will attract and reward the attention of beginners and experts alike.

## Figures and Tables

**Figure 1.**
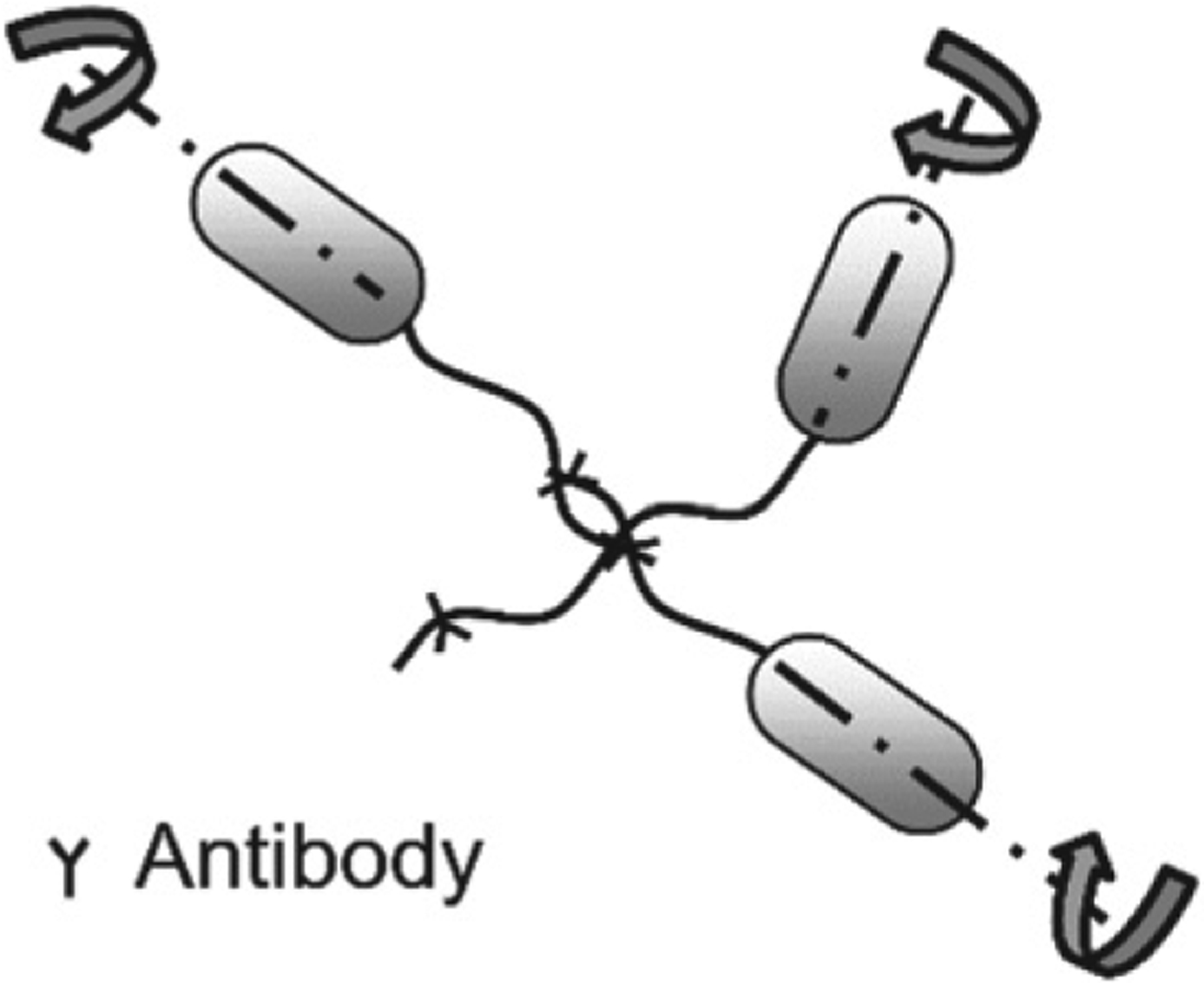
Linking of flagella on multiple singly-flagellated (monotrichous) cells with antibodies fails to stall the motors as the cell bodies are free to rotate.

**Figure 2.**
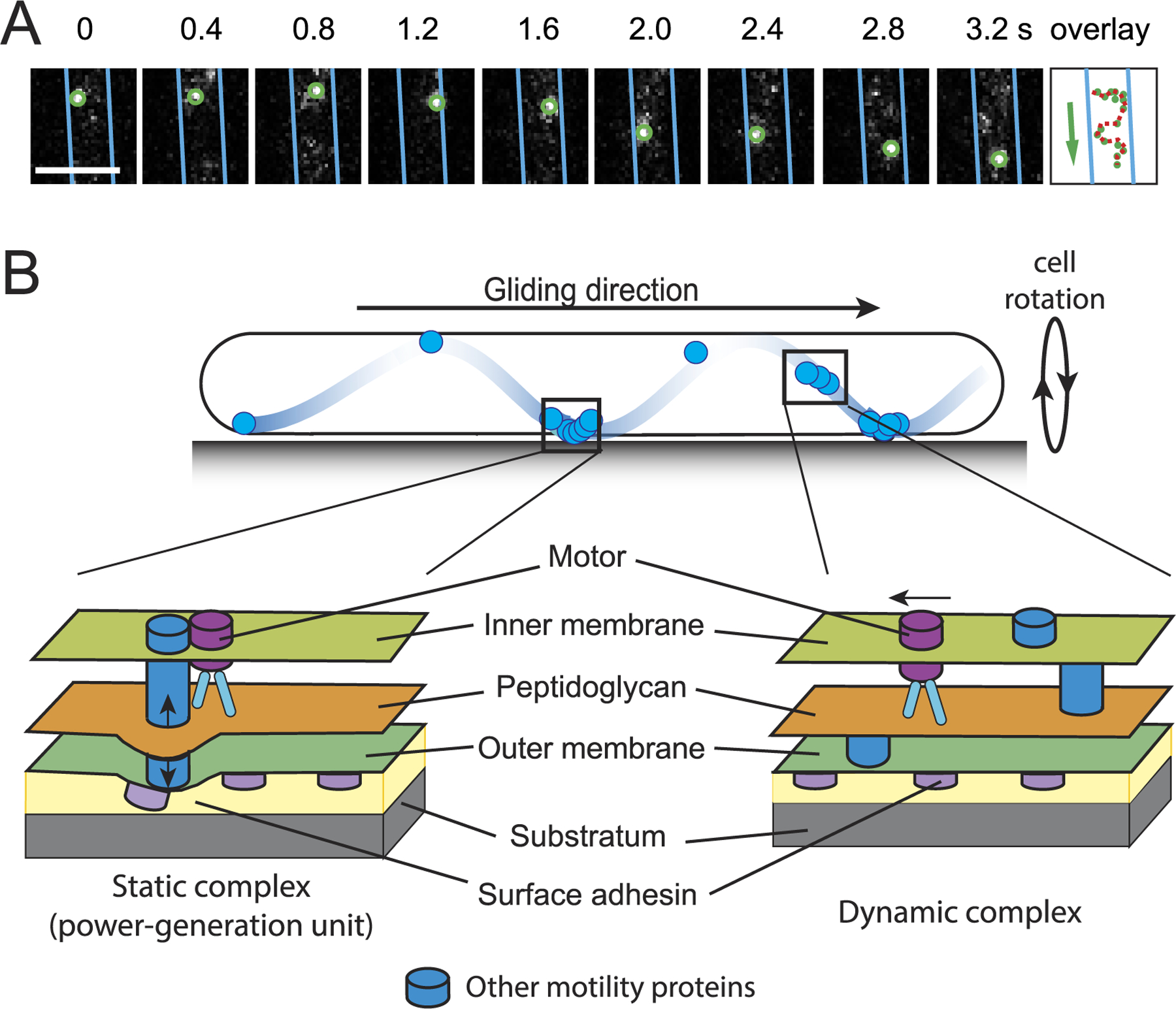
The gliding motility of *M. xanthus*. (A) 2D trajectory of a single gliding motor was recorded at 200 ms intervals. The cell boundaries were marked with blue lines. The overlay shows the positions of the motor in consecutive frames. Scale bar, 1 *μ*m. Adapted from reference [16]. (B) A schematic model for *M. xanthus* gliding. Motors carrying incomplete gliding complexes move rapidly along helical paths but do not generate propulsion (dynamic complexes). These motors stall and become nearly static relative to the substratum when they assemble into complete gliding machineries with other motor-associated proteins at the ventral side of the cell (static complexes). The static complexes exert force against putative outer membrane adhesins. As the adhesins slide, the cell moves forward and the cell body rotates. Adapted with permission from reference [17].

**Figure 3.**
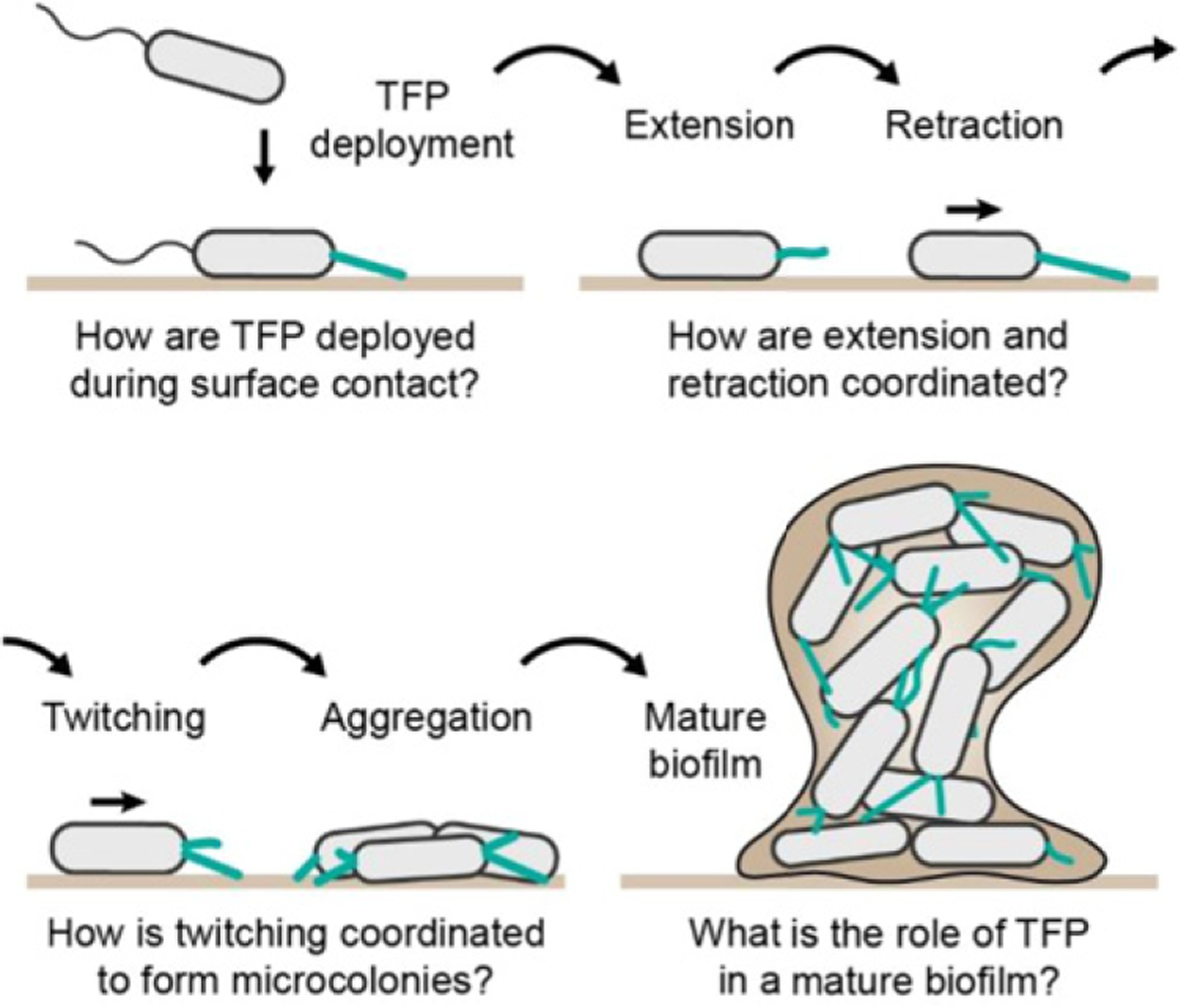
Known and hypothesized contribution of TFP and twitching during different stages of biofilm formation. TFP must rapidly deploy upon surface contact. Rounds of extension and retraction drive twitching motility, which ultimately lead to the formation of cellular aggregates. These microcolonies eventually mature into large, 3D biofilm in which the presence and potential function of TFP remain unclear.

**Figure 4.**
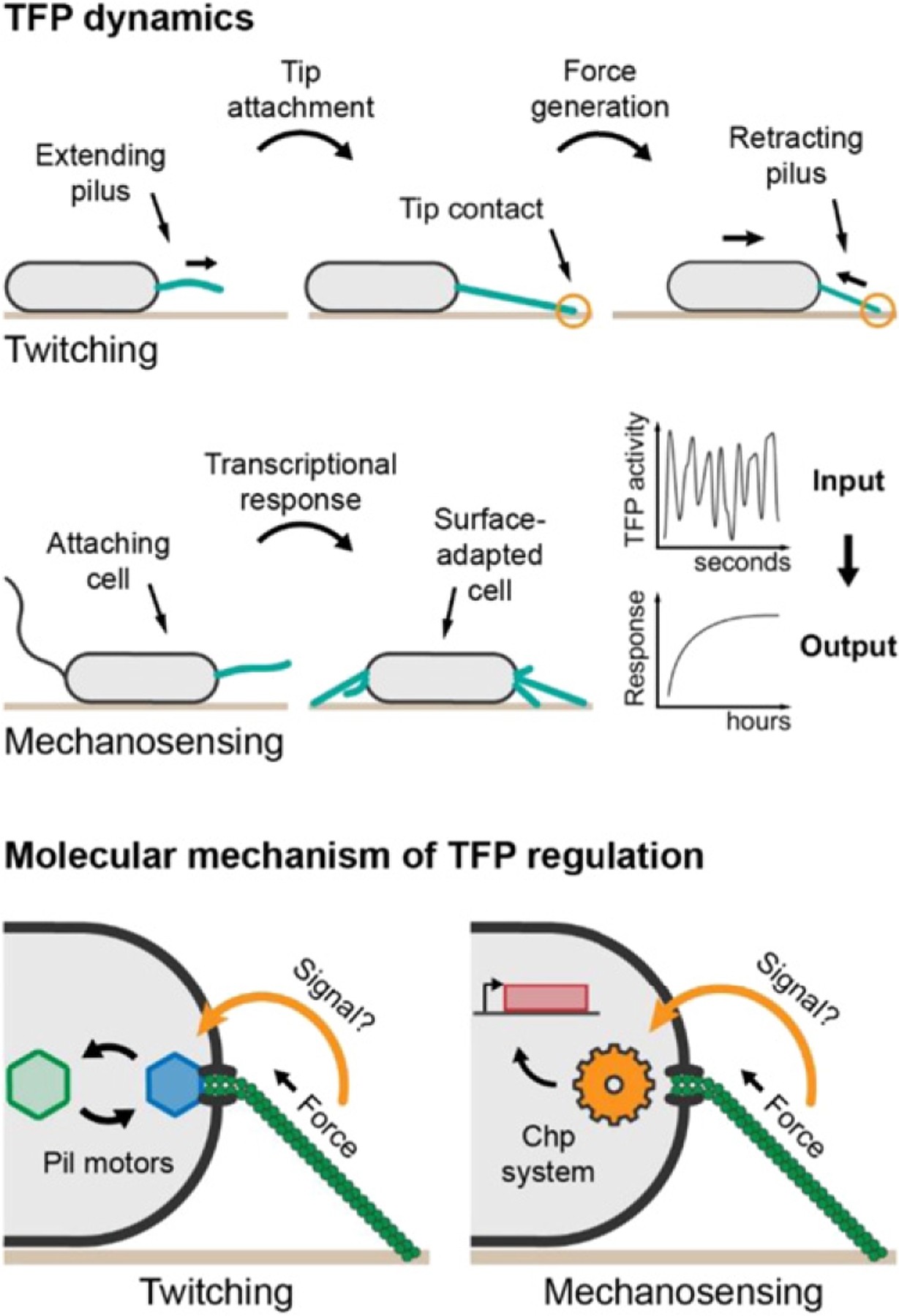
Dynamics of TFP during twitching and surface adaptation and potential signaling mechanism through conformational changes in the pilus induced by retraction force. Top panel: pilus retraction is synchronized with tip attachment to confer efficient twitching. Middle panel: short-term mechanical contact with the surface via TFP induces a long-term transcriptional response. Bottom panel: tension within the pilus, possibly changing its conformation, influences exchange of the pilus motors (left) as well as Chp signaling (right). Hexagons depict pili motors, the gear depicts the Chp system, and the red rectangle depicts a gene.

**Figure 5.**
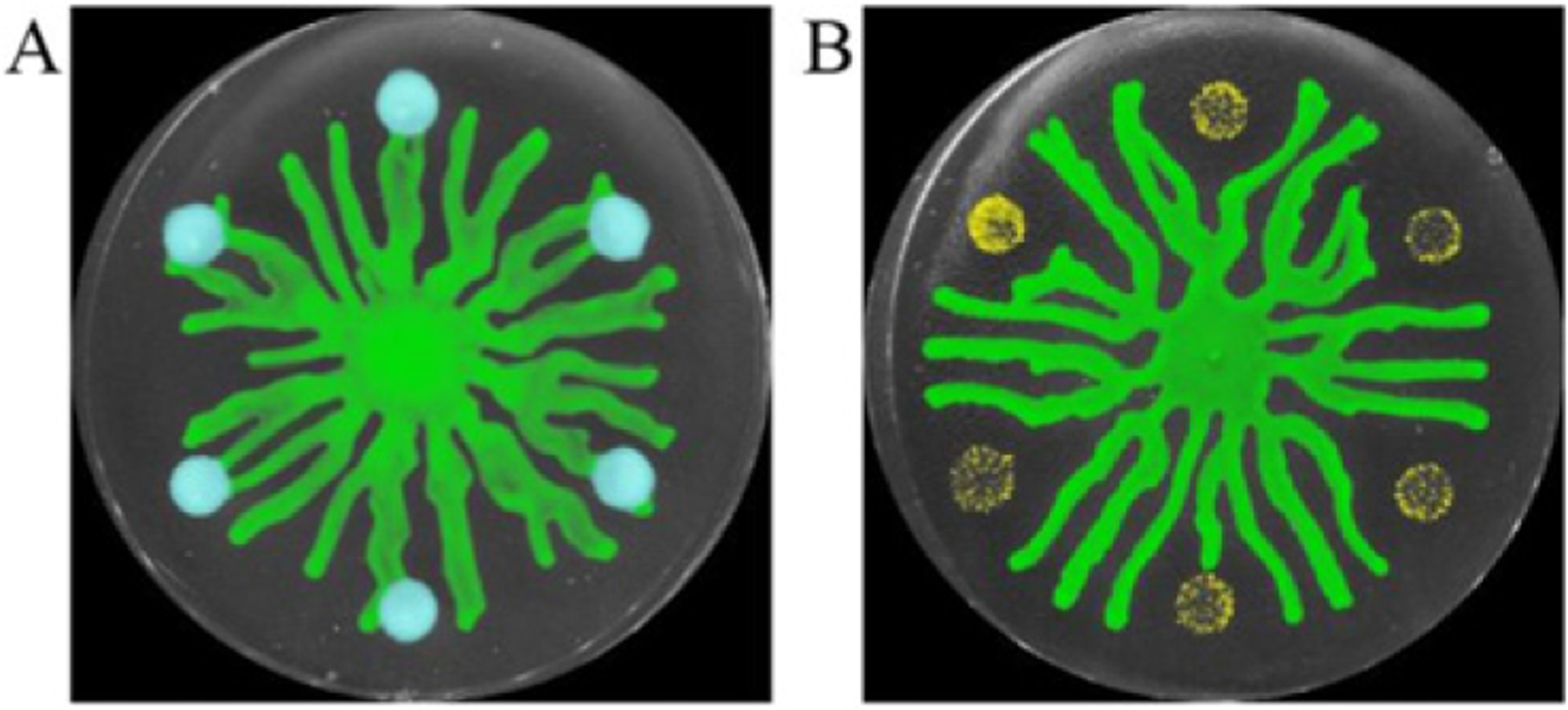
Spatial organization by a stress response in bacterial populations. (A) *P. aeruginosa* swarms (green) merge with unstressed sub-populations (blue). (B) *P. aeruginosa* swarms (green) are re-directed away from sub-populations that are infected by phage (yellow), which release the cell–cell signaling molecule PQS. Images are shown in pseudocolor.

**Figure 6.**
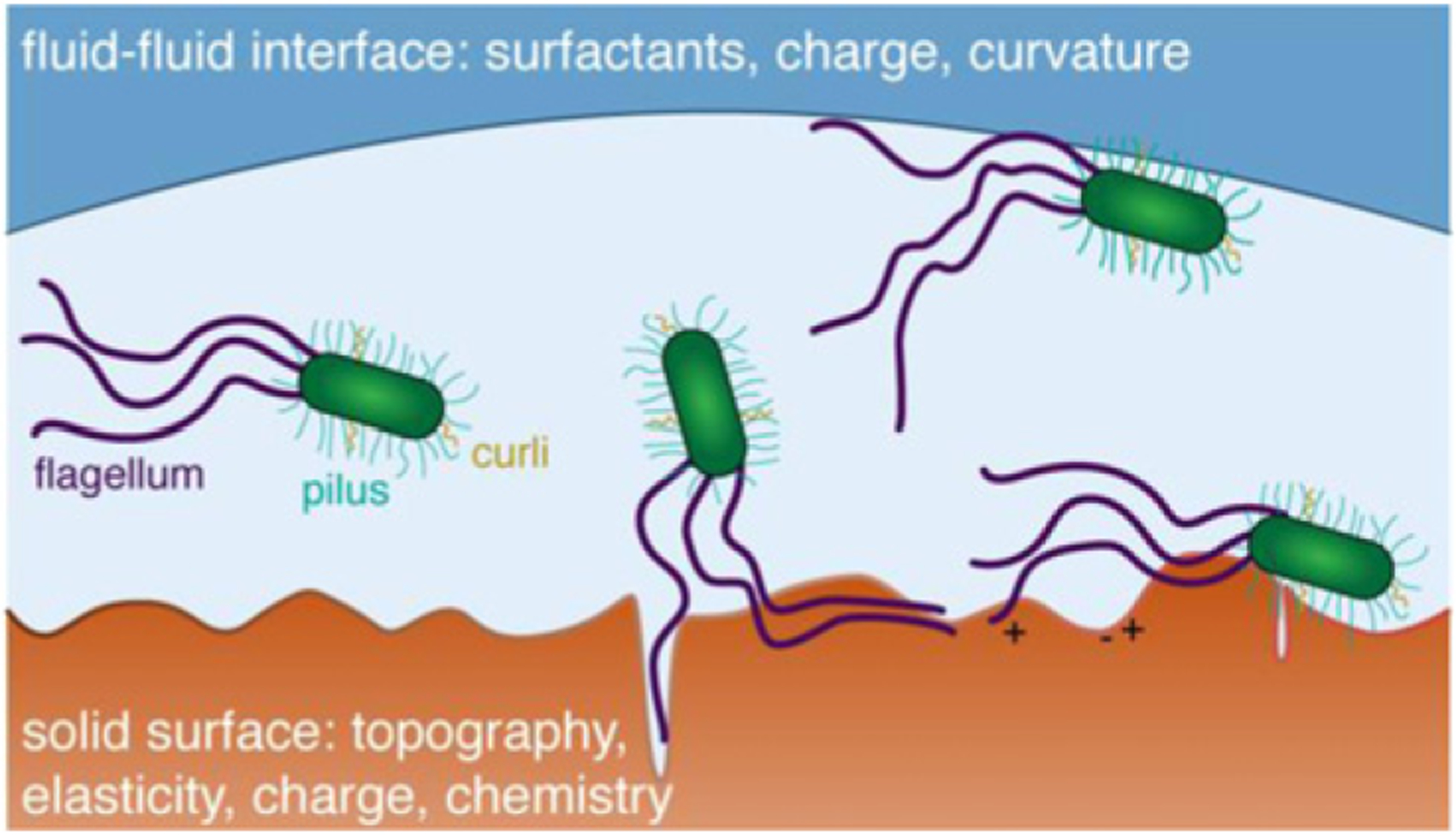
Schematic illustration of bacterial adhesion at liquid–solid and liquid–liquid interfaces. Fibrillar surface structures such as flagella, pili, or curli interact with heterogeneous surfaces (e.g. roughness, charge) to aid adhesion.

**Figure 7.**
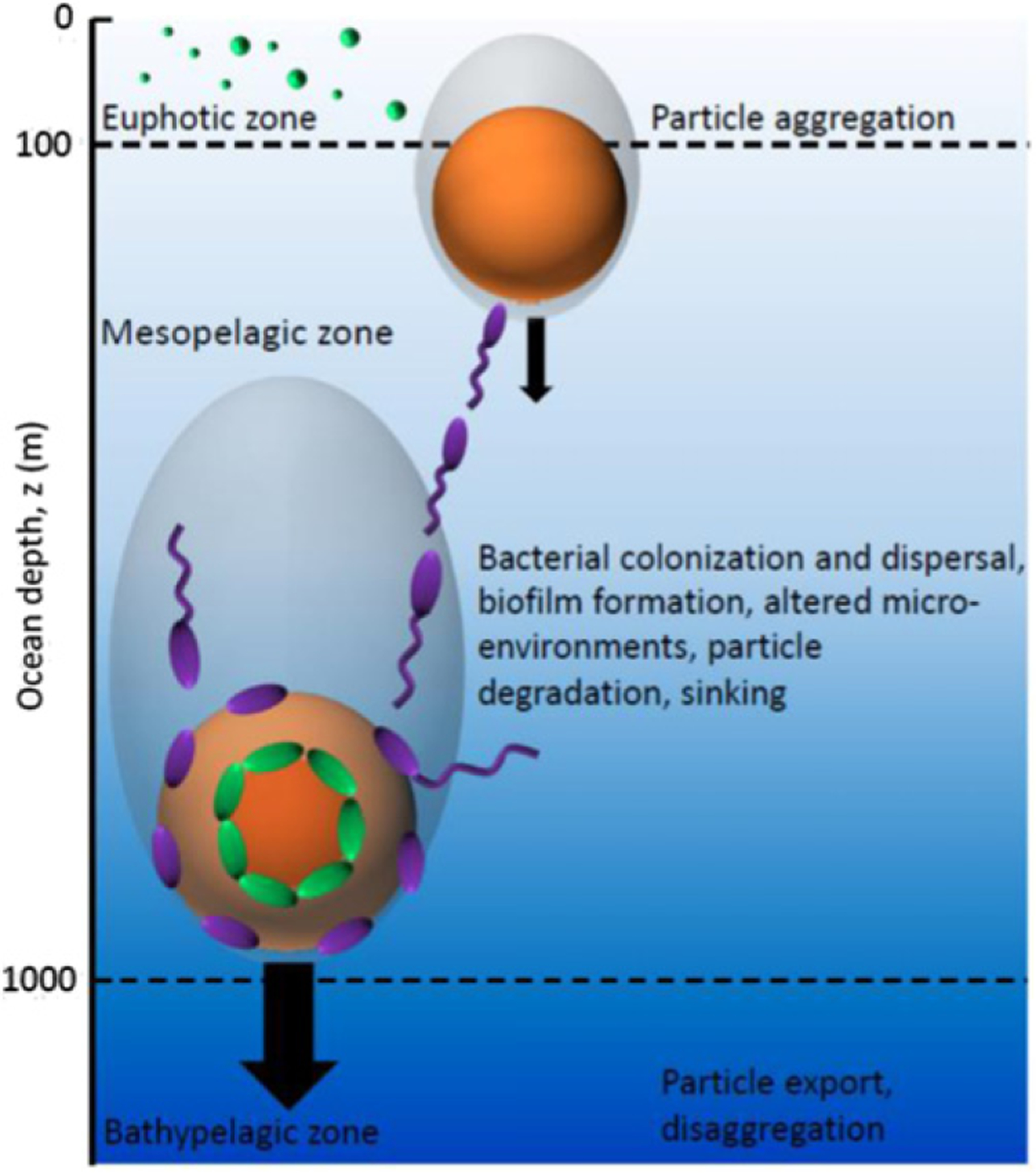
Organic particles, formed by the aggregation of fragments of decaying organisms, leave the euphotic zone, sink through the mesopelagic zone at a size-dependent rate, and are altered as they sink by bacterial activity. Cells adopt a variety of foraging strategies, including irreversible attachment to the particle surface with formation of biofilm (green cells), or a flexible strategy involving repeated colonization and dispersal [62] (violet cells). These processes regulate the biological pump, the process by which particulate carbon sinks and reaches the bathypelagic zone to be sequestered in the ocean depths for thousands of years.

**Figure 8.**
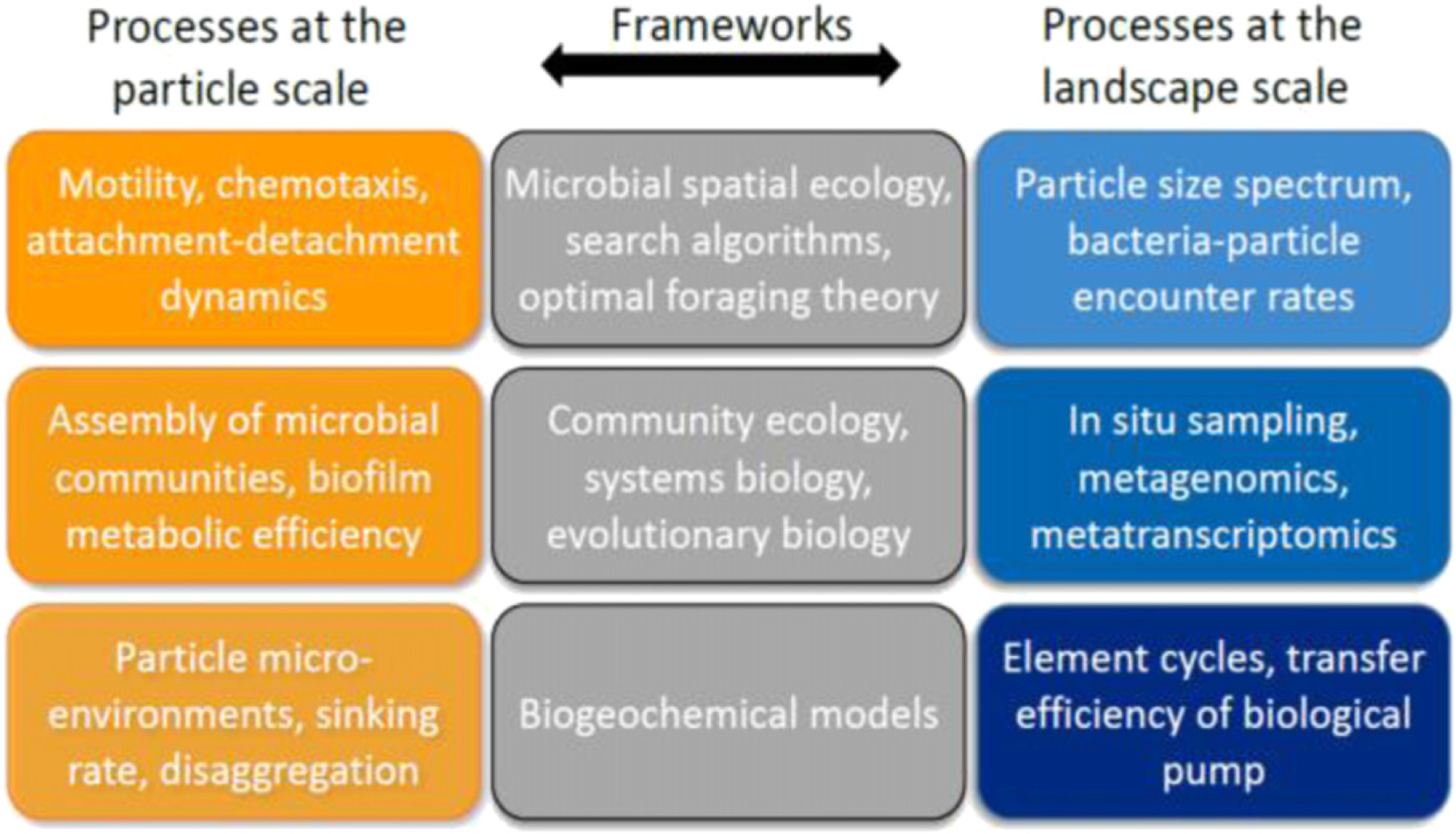
The study of bacterial communities on marine particles, given the multi-scale nature of the processes involved, requires an integrative approach, merging frameworks adopted in microbial spatial ecology with those of community ecology, systems biology and evolutionary theory.

**Figure 9.**
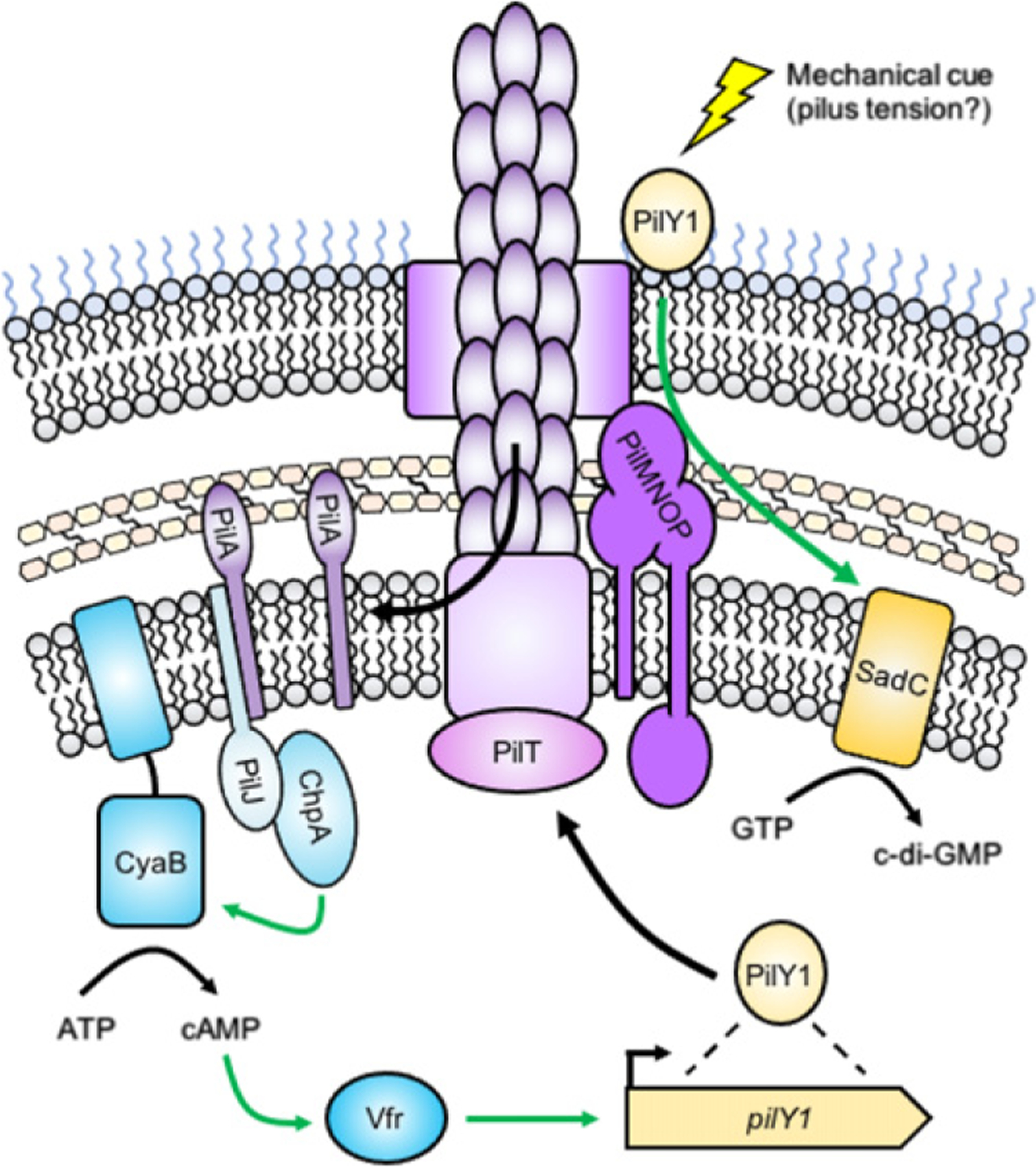
Surface sensing by TFP in *P. aeruginosa*. The pilus fiber is composed of the major pilin subunit PilA, which is polymerized from a pool of monomers that reside in the inner membrane. Pilus retraction by the motor PilT leads to PilA depolymerization and re-incorporation of PilA monomers into the inner membrane (left). PilA monomers are detected by the sensor PilJ, which activates ChpA. ChpA stimulates the activity of the adenylate cyclase CyaB, leading to the production of 3′,5′-cyclic adenosine monophosphate (cAMP). cAMP activates the transcription factor Vfr, which upregulates production of the pilus biogenesis protein PilY1 (right). PilY1 is transported to the cell surface via the action of the pilus. The von Willebrand factor type A (VWFa) domain of PilY1 is thought to sense a mechanical cue, such as pilus tension during retraction due to surface contact. In response, PilY1 stimulates the DGC SadC through the pilus alignment subcomplex, composed of PilMNOP. SadC produces cyclic-3′,5′-dimeric guanosine monophosphate (c-di-GMP), the major signaling molecule that promotes a surface-associated lifestyle. Green arrows indicate positive regulatory effects.

**Figure 10.**
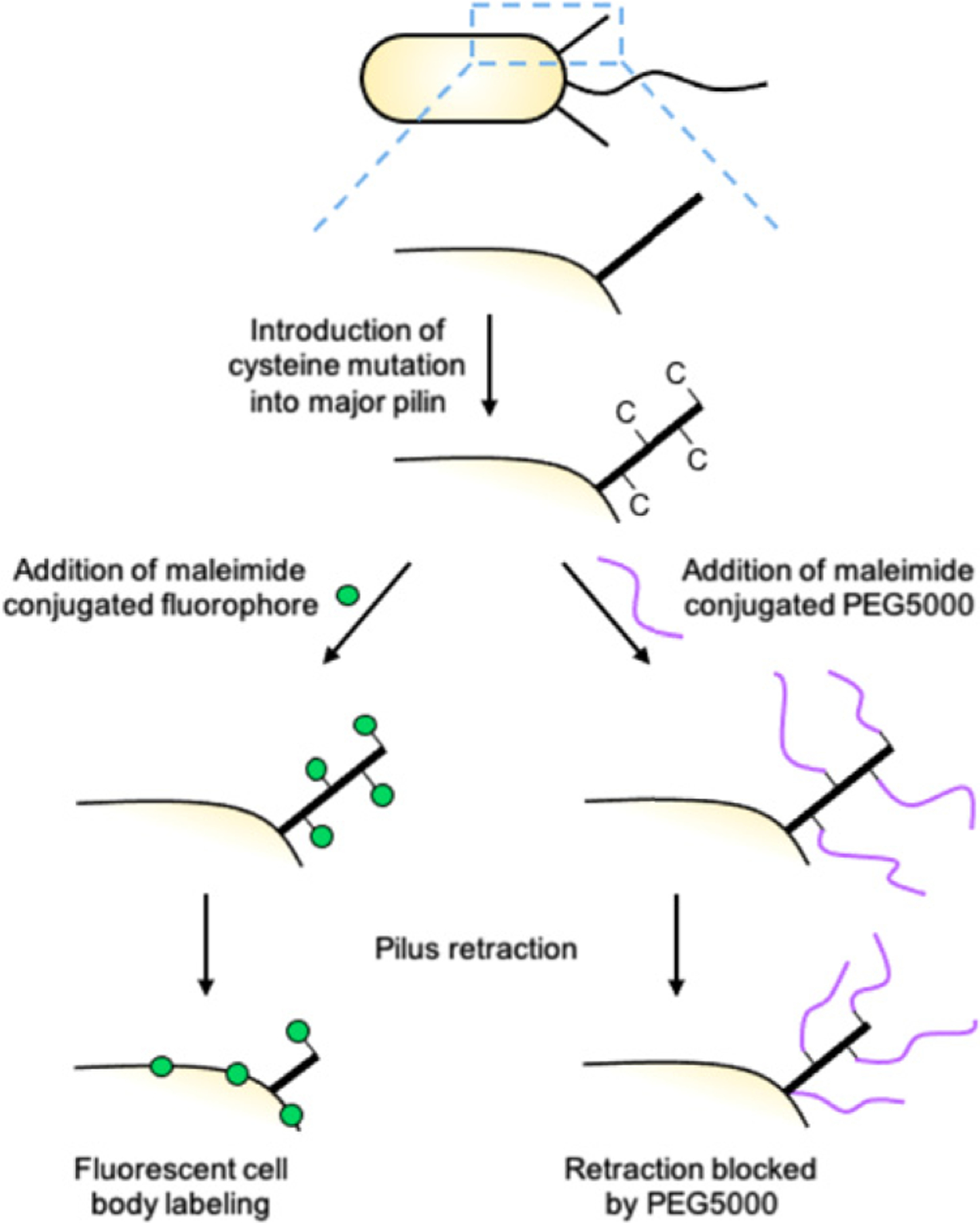
Functionalization of TFP by cysteine mutation and maleimide click chemistry. A cysteine (C) mutation is introduced into the major pilin subunit PilA that does not affect pilus function or dynamics. Cysteine-mutagenized pili can be covalently linked to thiolreactive maleimide conjugants, including fluorophores (left) and/or bulky high molecular weight polyethylene glycols (PEGs; right). Fluorescent maleimide conjugants allow for the visualization of pilus dynamics by epifluorescence microscopy. Labeled pilins are re-incorporated into the inner membrane upon pilus retraction, leading to cell body fluorescence. High molecular weight PEGs, such as PEG5000, block pilus retraction due to physical obstruction, leading to the upregulation of surface sensing phenotypes in the absence of surface contact. These maleimide conjugants can be combined to simultaneously fluorescently label pili and block their retraction.

**Figure 11.**
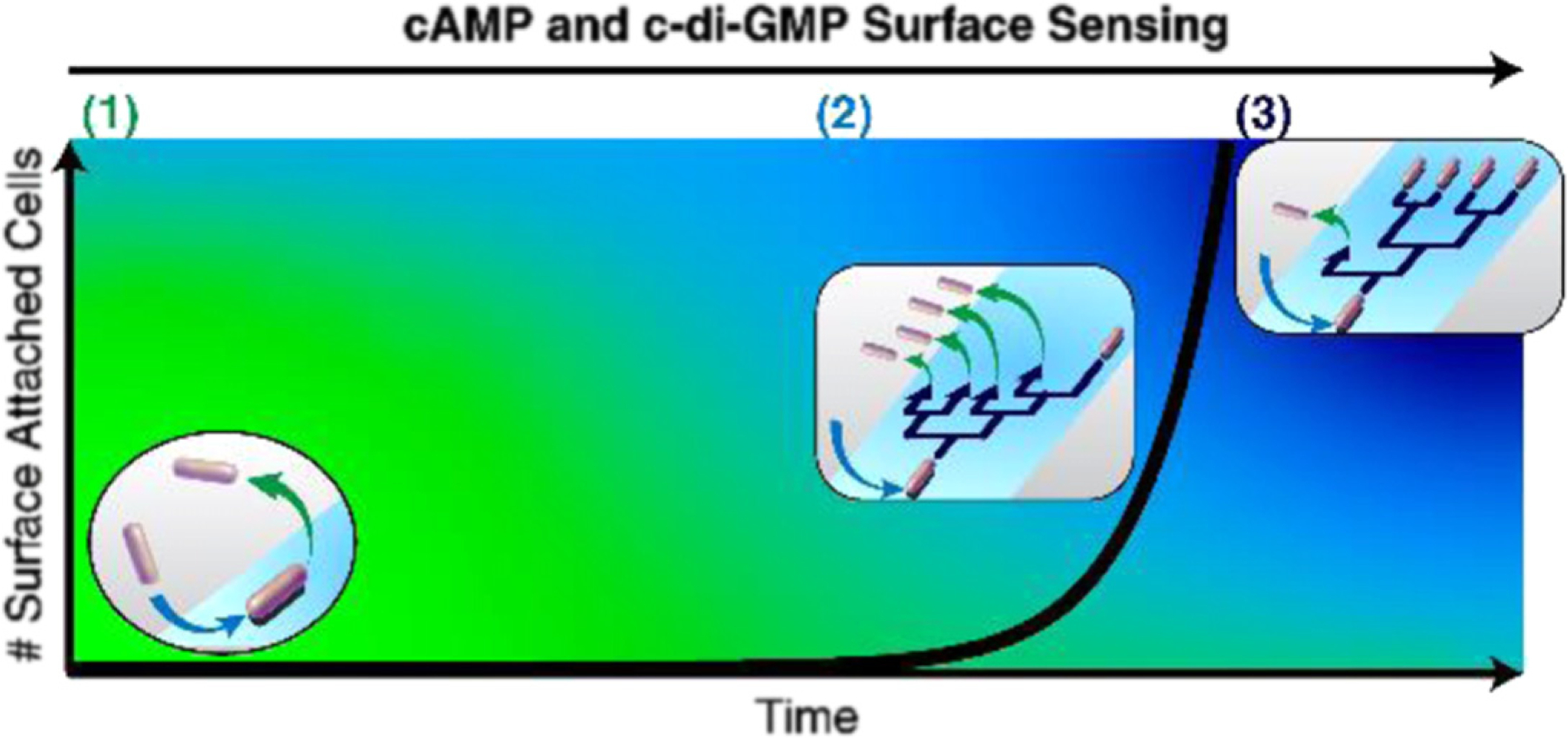
Model for multigenerational lineages at different stages of biofilm growth. Stage (1): cells first encountering a surface are ‘surface-naïve’ and most likely detach from the surface without dividing. During this period, the surface population is constant and/or near-zero. Stage (2): as cells repeatedly encounter the surface, they become ‘surface-sentient’ and eventually stay on the surface long enough to divide. However, detachment is still prevalent, so many families exhibit one-legged division-branching, where one of the daughter cells detaches after division. During this period, the surface population starts to rise, albeit slowly. Detachment is prevalent in stages (1) and (2), so both of these stages are considered to be reversible attachment. Stage (3): eventually, a subpopulation of lineages appears with less detachment and more two-legged division-branching, where both daughter cells stay after division. During this period, the surface population begins rapidly rising at an exponential rate. This signifies the onset of irreversible attachment.

**Figure 12.**
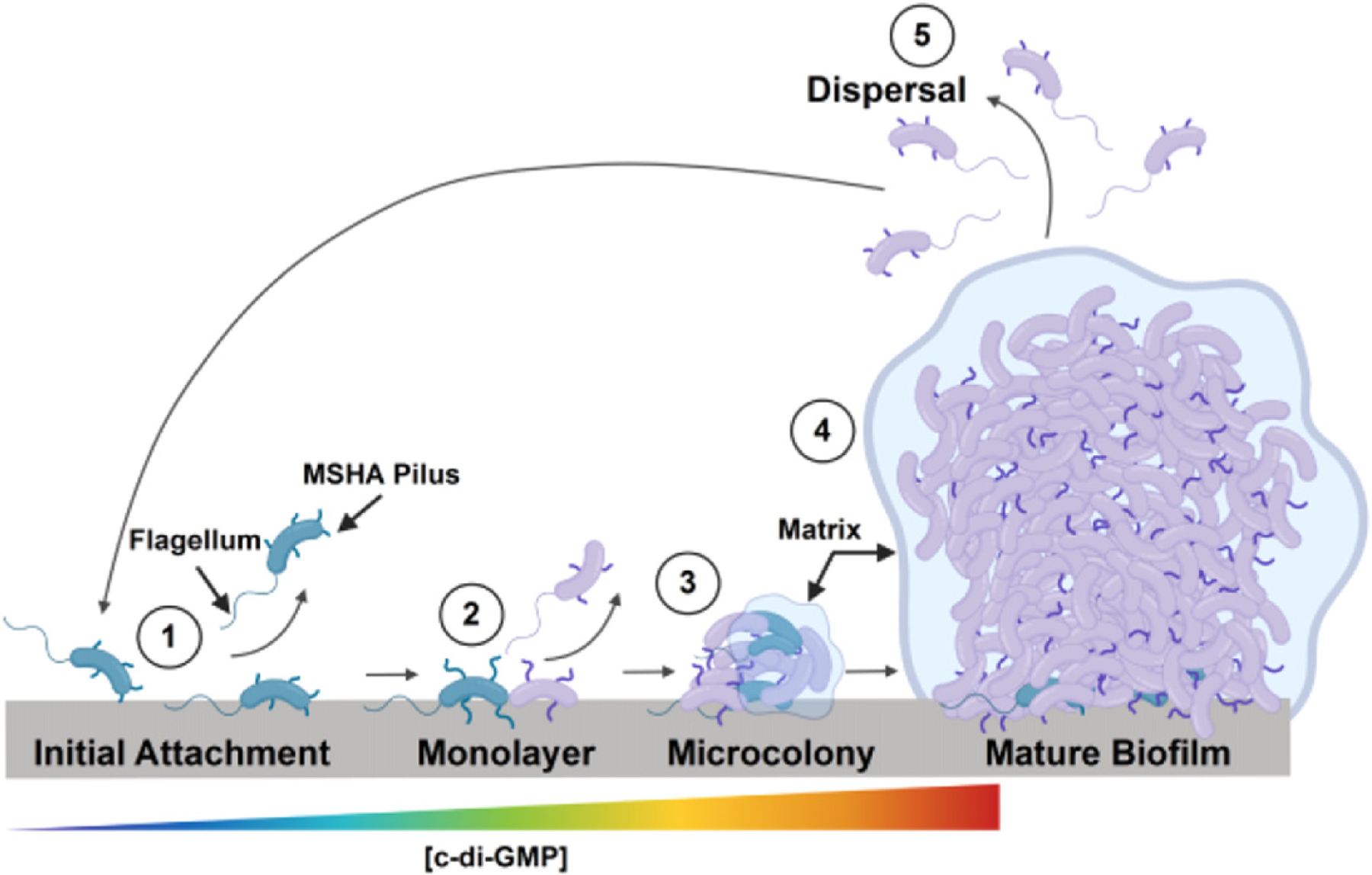
Schematic of *V. cholerae* biofilm formation. Environmental colonization and surface attachment are dependent upon production of MSHA pili (1). Initial surface interactions initiate an increase in intracellular c-di-GMP levels that promote cessation of flagellar motility and further MSHA production to anchor the cell to the surface (2) that allows for microcolony formation and biofilm maturation (3 and 4). The role of c-di-GMP in biofilm dispersal (5) remains to be established (model created with BioRender.com).

**Figure 13.**
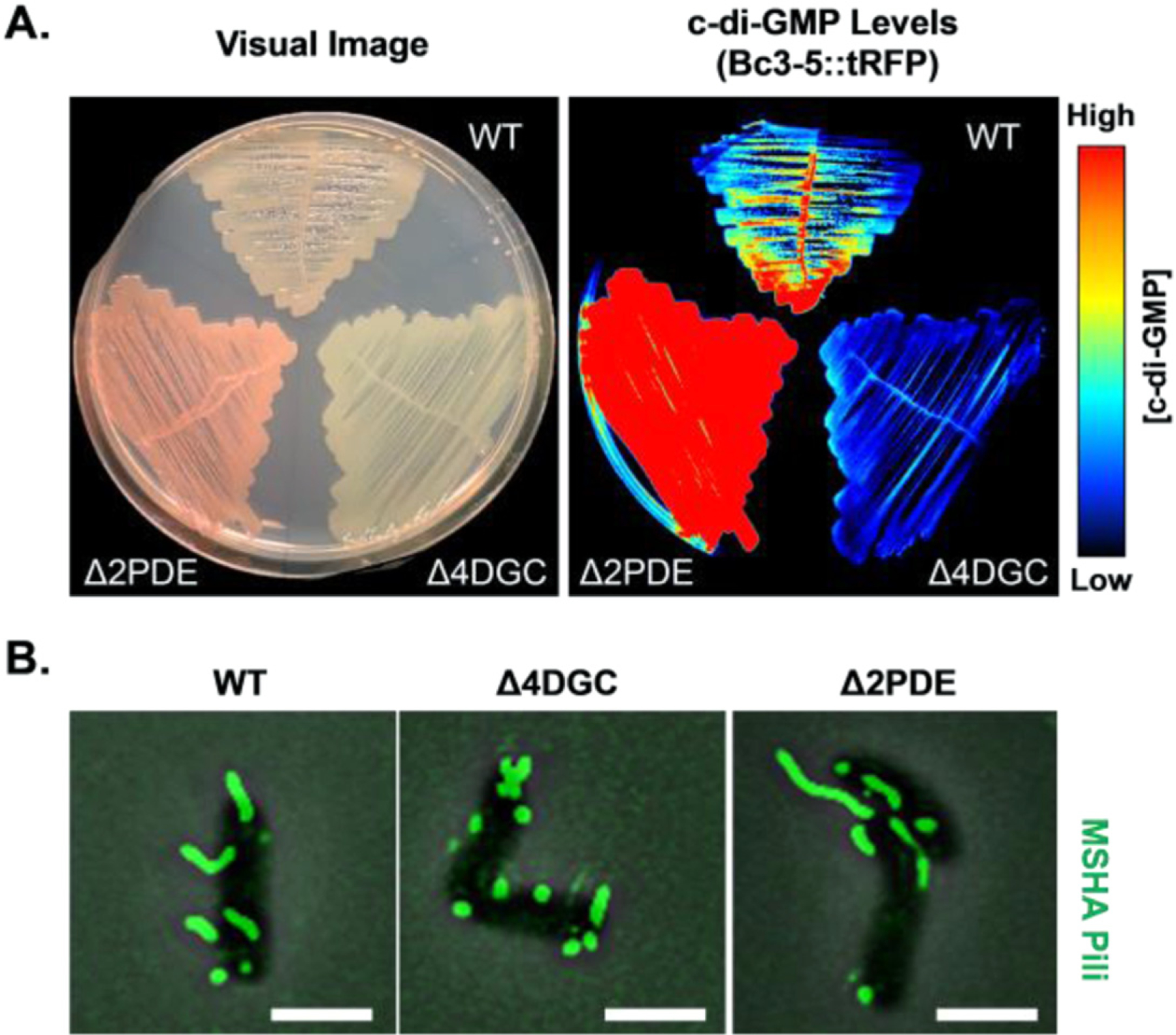
Visualizing intracellular c-di-GMP levels, and impacts on MSHA pilus production. (A) c-di-GMP levels in WT, Δ4DGC (low c-di-GMP), and Δ2PDE (high c-di-GMP) determined via Bc3-5 c-di-GMP-responsive biosensor. (B) Heightened intracellular c-di-GMP levels promote cell–surface MSHA production, as visualized via thiol-reactive dyes. Scale bar = 2 *μ*m.

**Figure 14.**
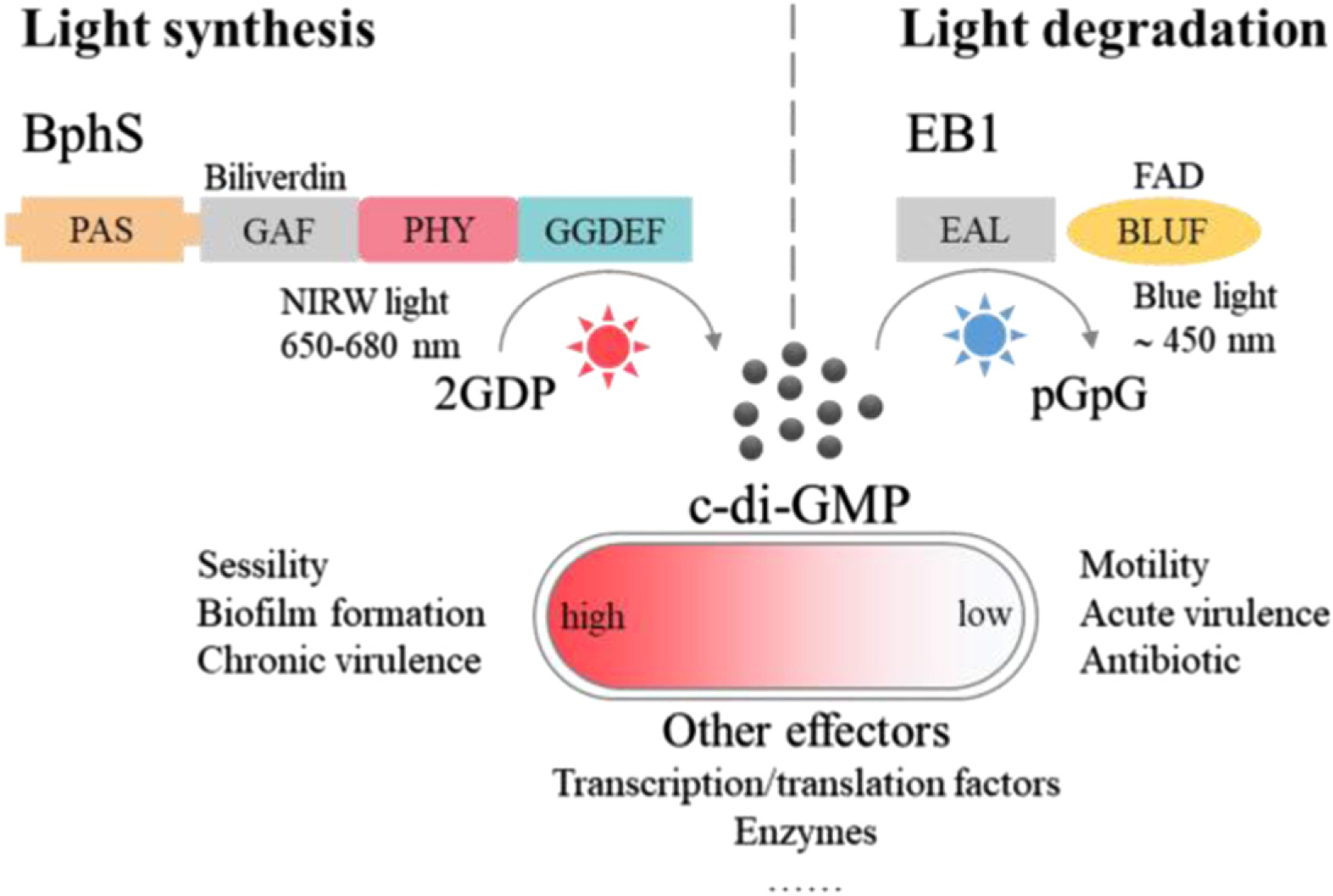
Engineered light-activated DGC and PDE photoreceptors for the synthesis and degradation of c-di-GMP to optogenetic control of many aspects of bacterial physiology and behavior such as the transitions of motile-to-sessile and acute-to-chronic virulence lifestyle.

**Figure 15.**
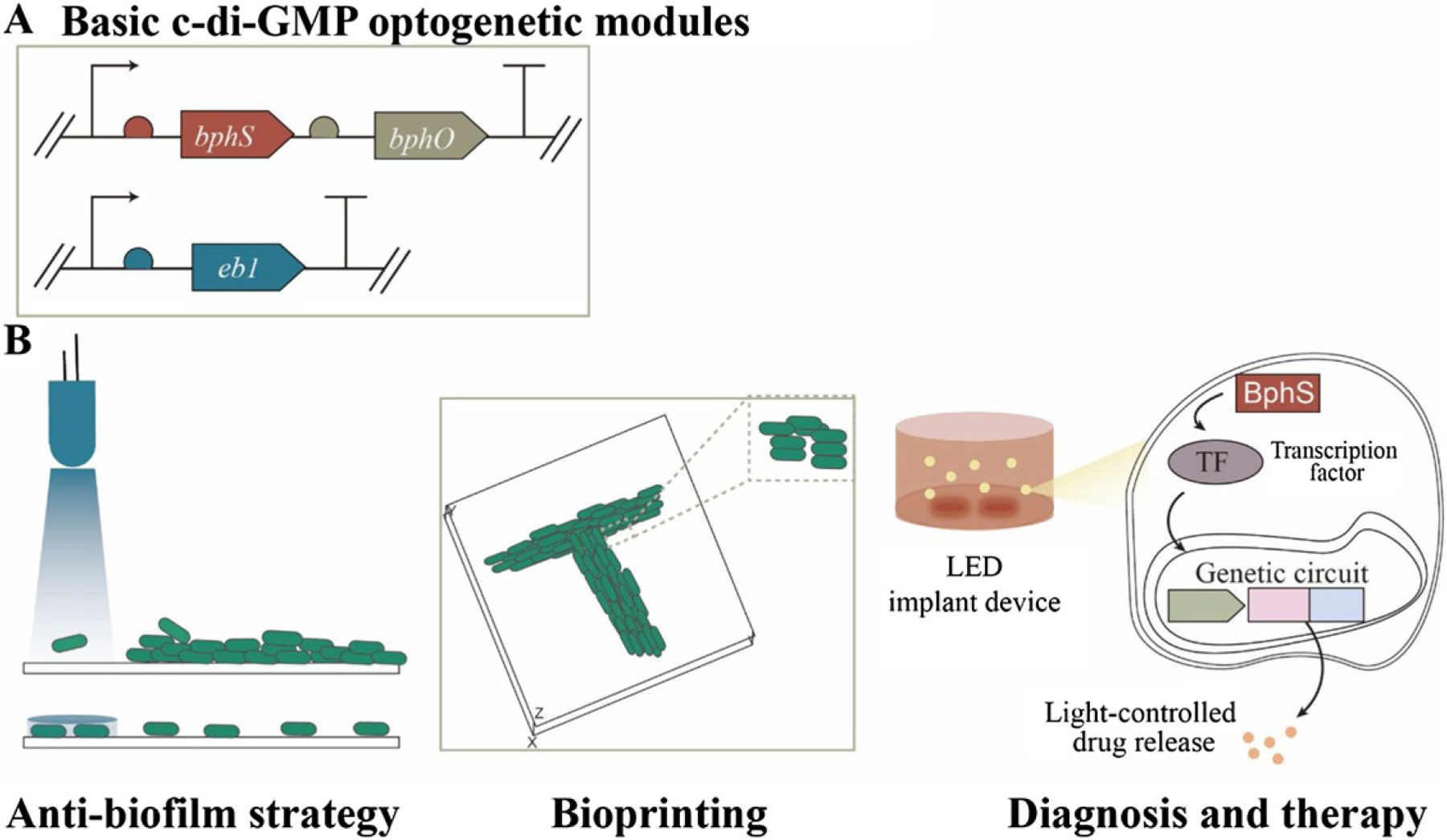
Basic c-di-GMP-based optogenetic modules (A) and the applications in various fields for distinctive purposes (B).

**Figure 16.**
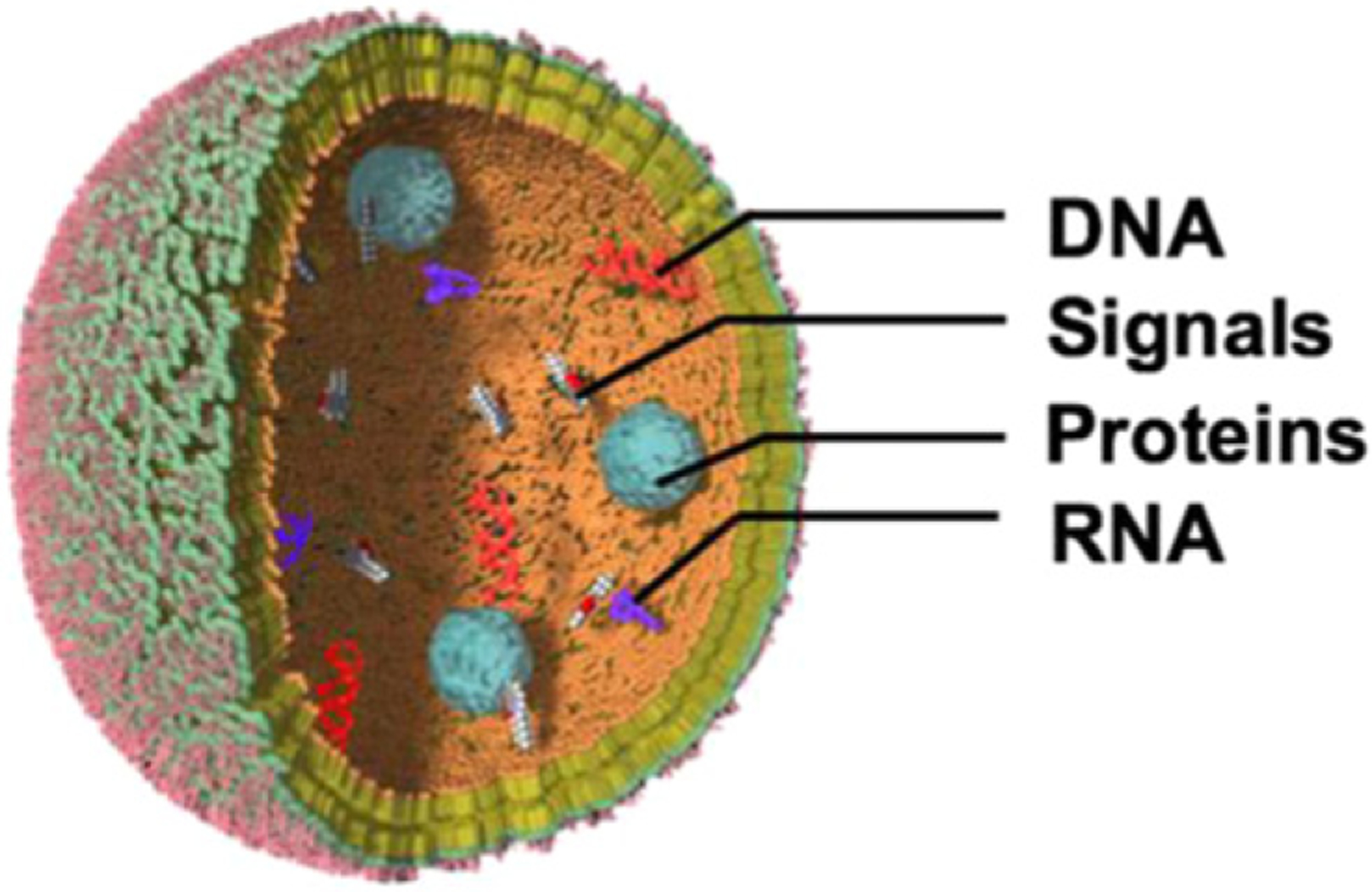
Bacterial MV. MVs can enrich and transport cell components and products including signaling molecules.

**Figure 17.**
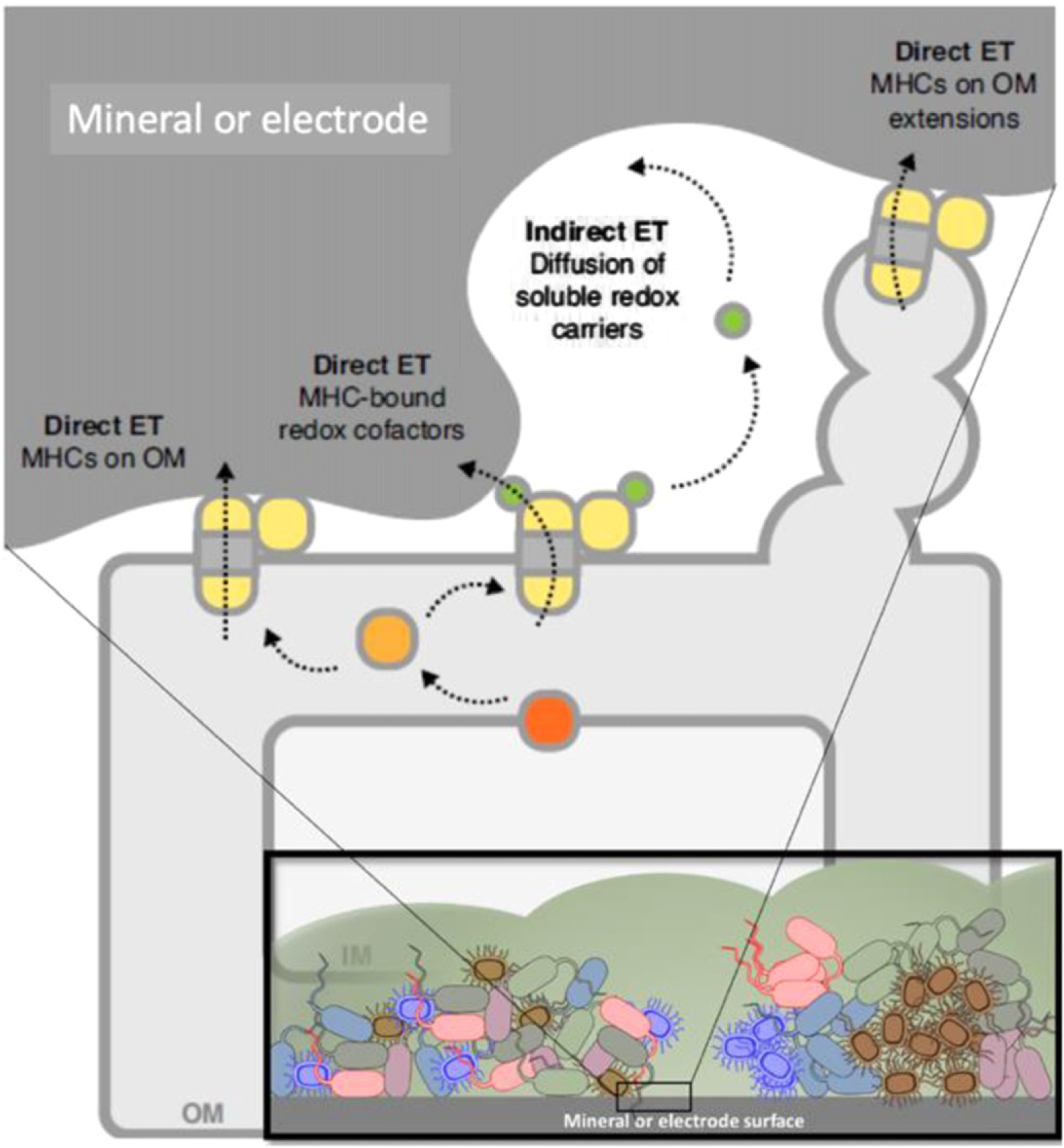
Electroactive biofilms (inset) are complex and can feature a number of microbe–surface EET mechanisms [110], including soluble redox shuttles and MHCs on the cell surface or organized on membrane extensions. Figure based on mechanisms identified in *Shewanella oneidensis* MR-1. Reproduced with permission from Elsevier.

**Figure 18.**
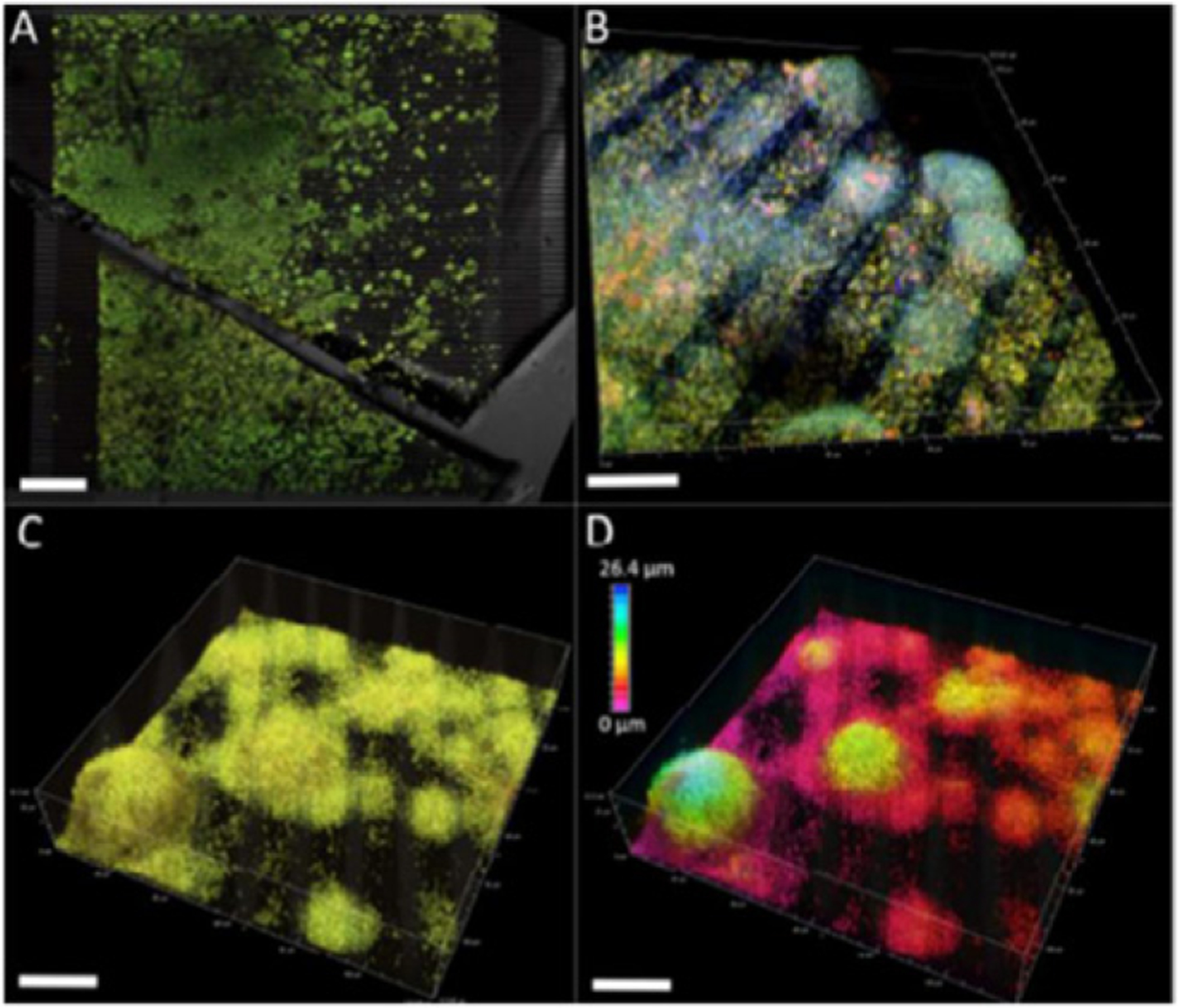
A CO_2_-reducing cathode biofilm cultivated on an IDA surface. Electrodes are 10 *μ*m wide and separated by 5 *μ*m gaps [116]. Staining and confocal imaging emphasize a variety of cell distribution patterns. (A) LIVE/DEAD staining of a thick cathode community. Scalebar = 300 *μ*m. (B) FISH-CLSM of *Ca. Tenderia electrophaga* (green/teal), *Alphaproteobacteria* (orange), and *Gammaproteobacteria* (red). (C) LIVE/DEAD staining of the community on an IDA surface. (D) The same image as (C) but with a color scale to illustrate distance from the electrode surface. (B)–(D) scalebars = 25 *μ*m. Reproduced with permission from the Royal Society of Chemistry.

**Figure 19.**
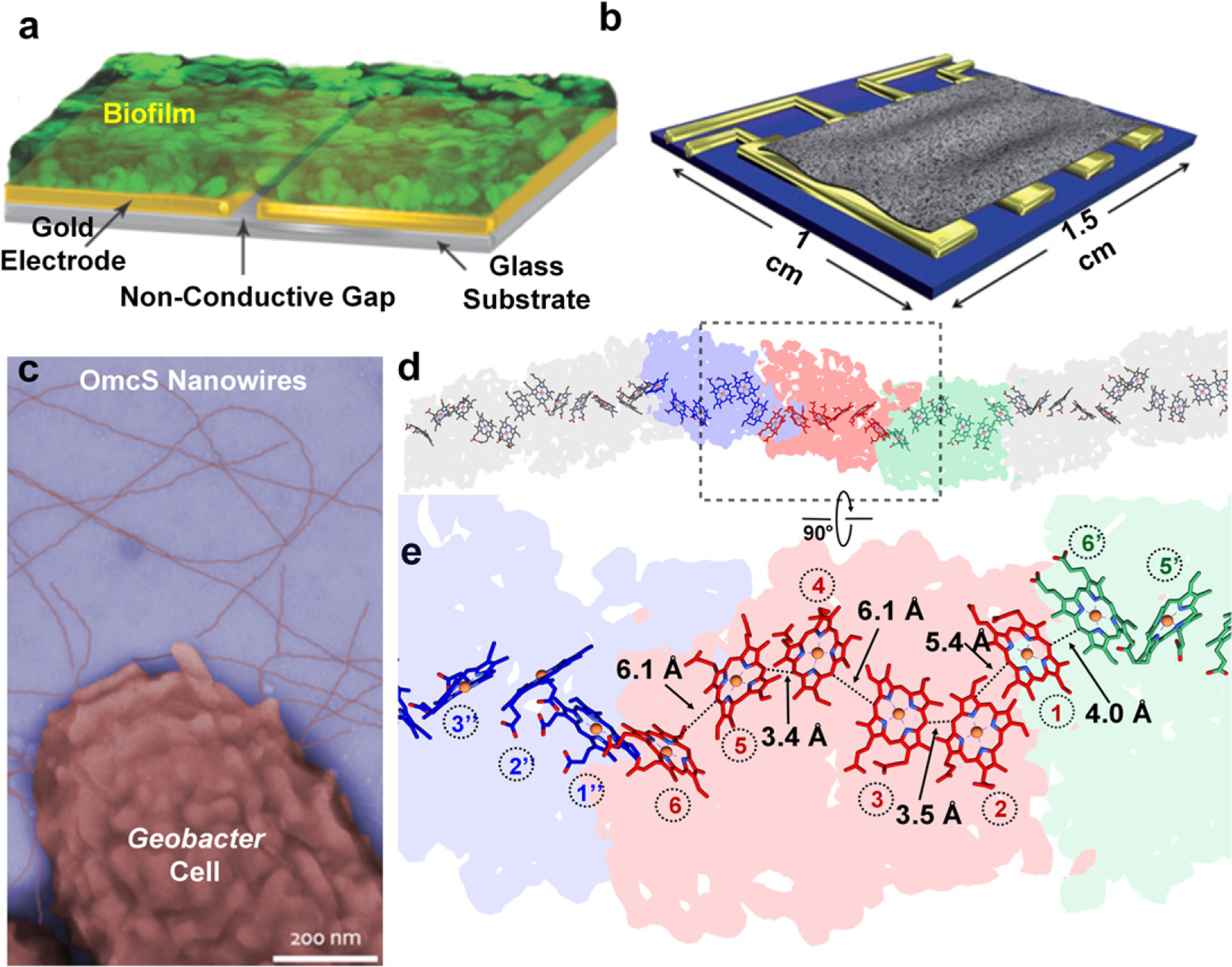
(a) Strategy to measure *in situ* electronic conductivity of living biofilms. (b) Four-electrode setup to measure intrinsic, contact-free conductivity. (c) Colorized TEM image of intact OmcS nanowires attached to cells. (d) and (e) Cryo-EM structure of the OmcS nanowire with region in color magnified. (a) and (b) Reprinted from [120, 121] with permission from Springer Nature. (c)–(e) Reprinted from [112] with permission from Cell Press.

**Figure 20.**
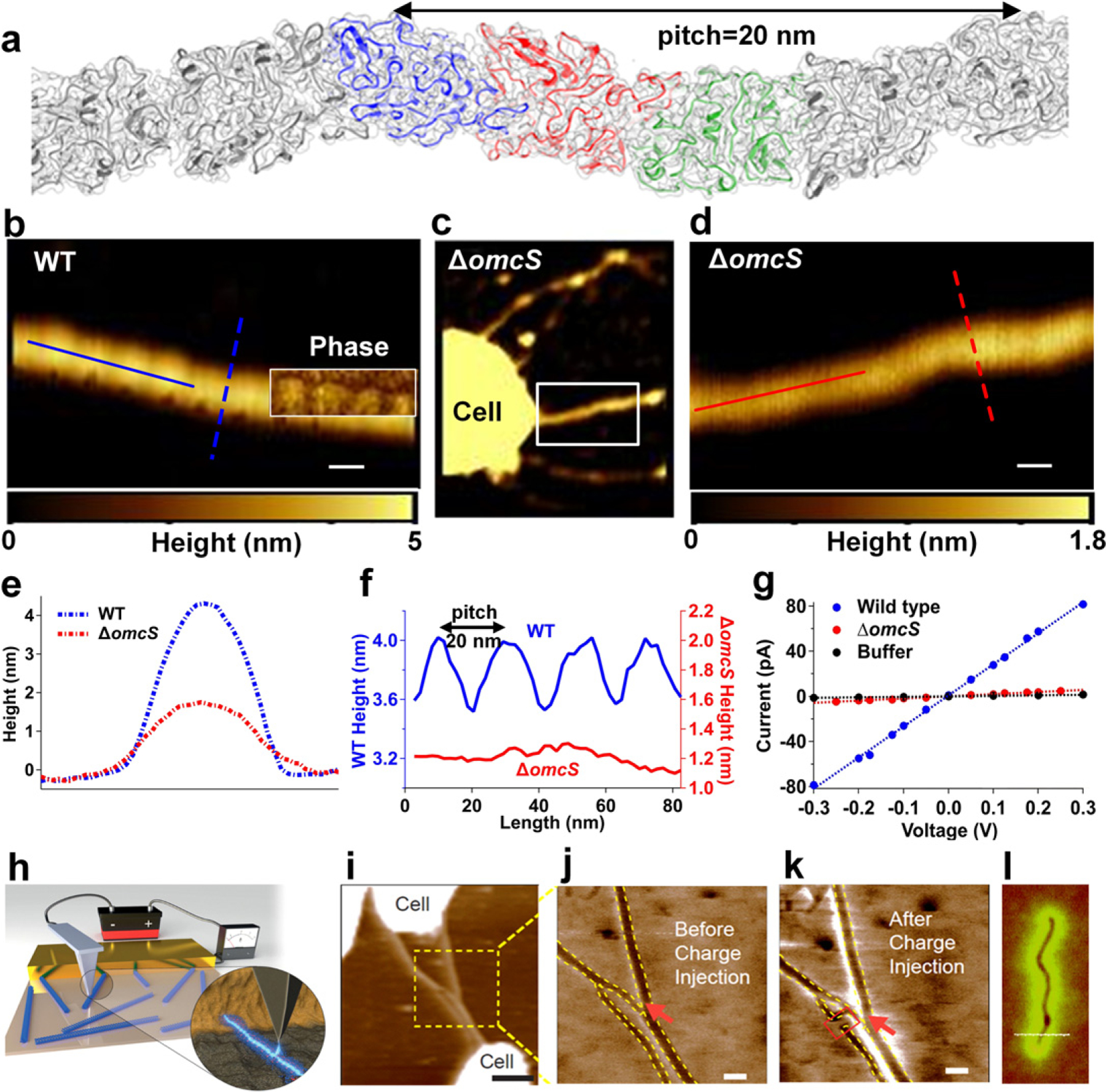
(a) Cryo-EM structure of the OmcS nanowire. (b)–(f) AFM images and corresponding height profiles. Scale bars, 20 nm. (g) Current–voltage profile across gold electrodes. Scale bar, 500 nm. (h) Schematic of EFM. (i) AFM height and EFM phase image of (j) and (k) *Geobacter* nanowires and (l) carbon nanotube (a)–(g) reprinted from [112] with permission from Cell Press. (h)–(k) Modified and reprinted from [130, 131] respectively with permission from Springer Nature. (l) Reprinted from [132] with permission from American Physical Society.

**Figure 21.**
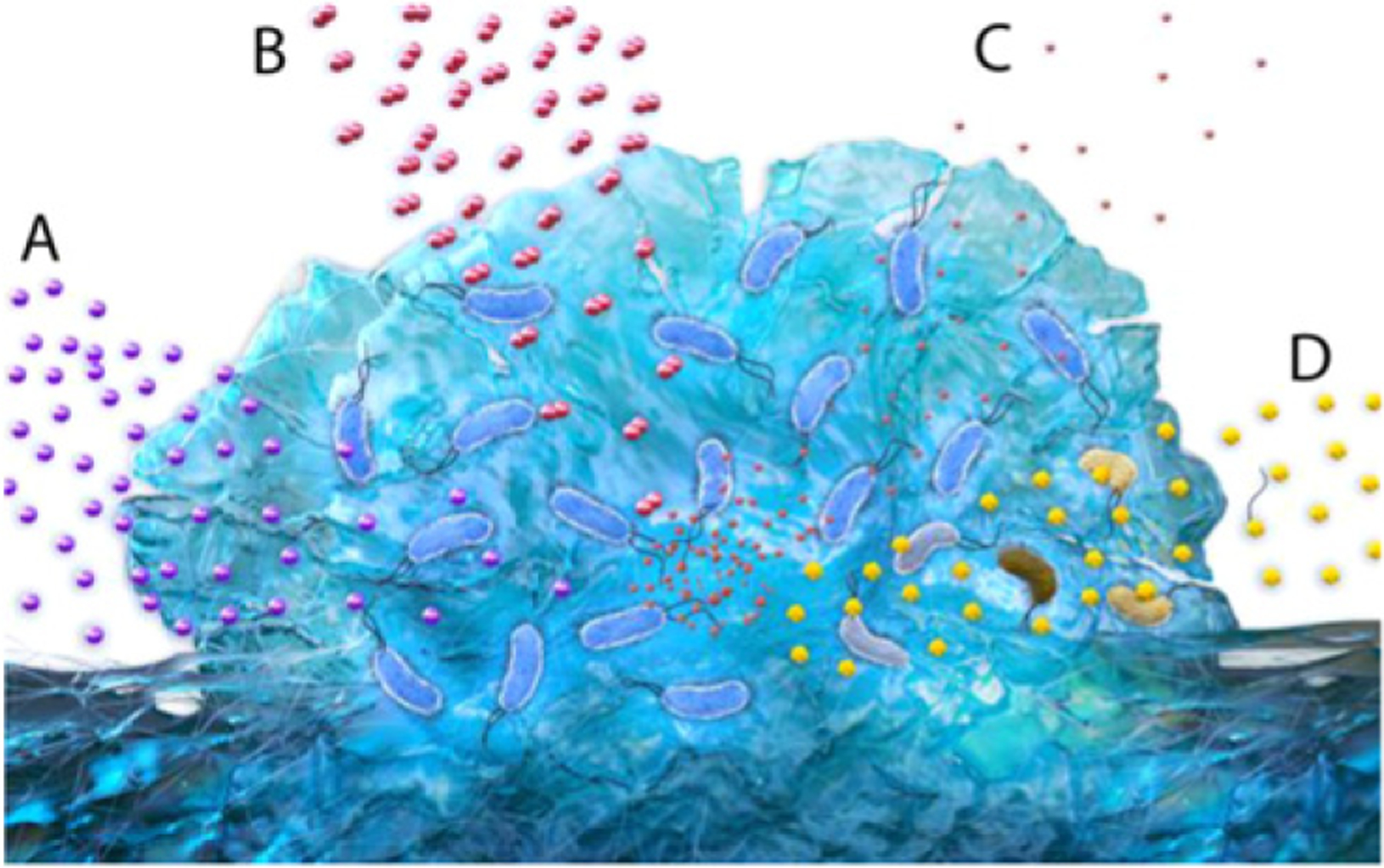
Metabolic heterogeneity arises in the complex chemical environments of bacterial communities. Gradients of nutrients (A) and oxygen (B) starve cells far from the growth medium of C, N, and/or P and electron acceptors. Waste products and other metabolites (C) generated within the community further alter the local environment sensed by the cells. These factors all affect the response of constituent bacteria to exogenous antimicrobial compounds (D).

**Figure 22.**
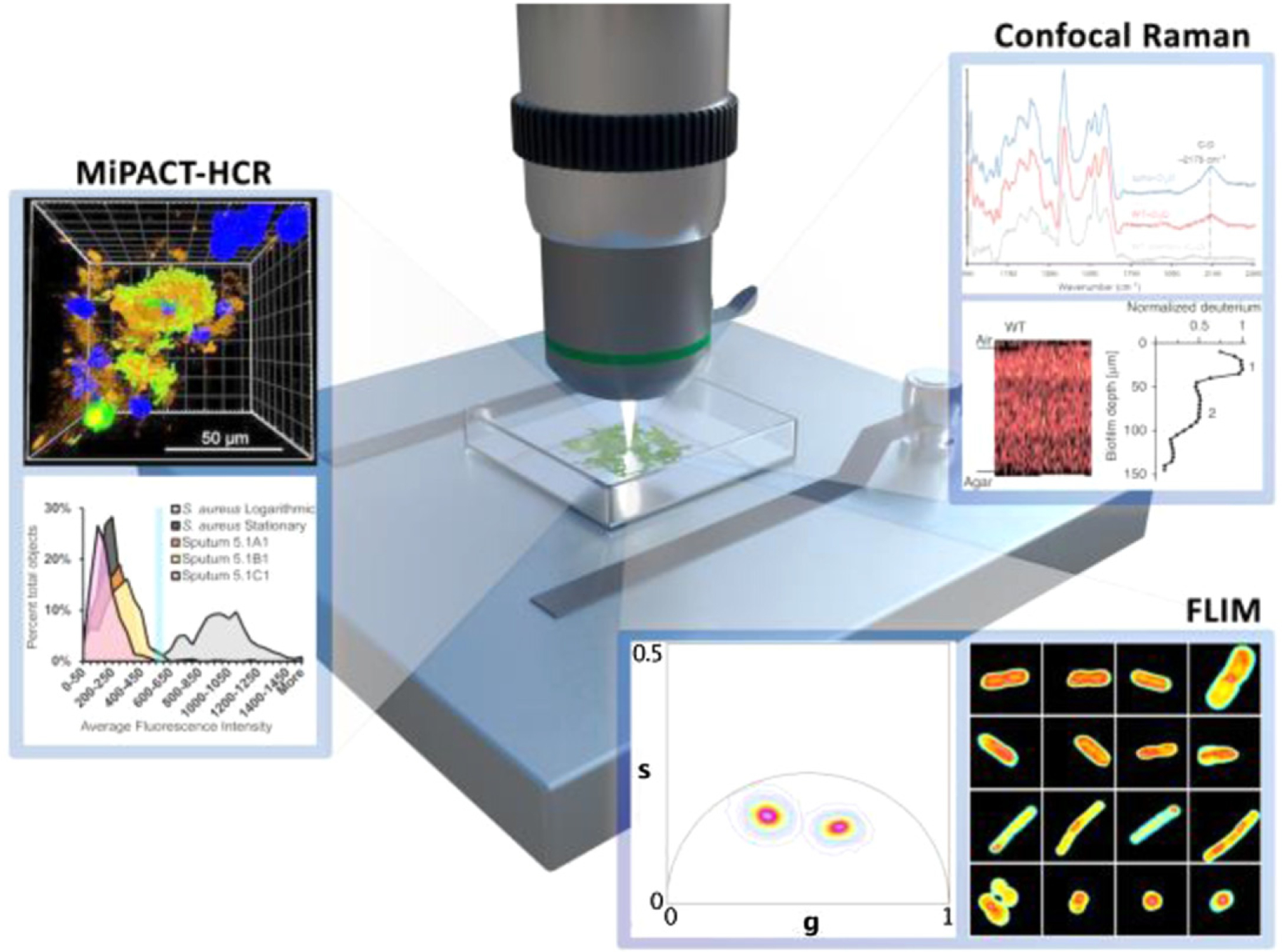
Microscopic imaging techniques using destructive staining and sample preparation methods (MiPACT-HCR) provide static insight into the metabolic heterogeneity and biogeography of samples in complex environments. Non-destructive (confocal Raman) and label-free (FLIM) spectroscopic imaging techniques promise to provide the spatial and temporal resolution required to map metabolic heterogeneity and dynamics associated with antibiotic tolerant and persister phenotypes in bacterial communities. Data panels adapted from references with permission: MiPACT-HR panel adapted from [142]; confocal Raman panel adapted from [144]; FLIM panel reprinted from [145]. CC-BY 4.0.

**Figure 23.**
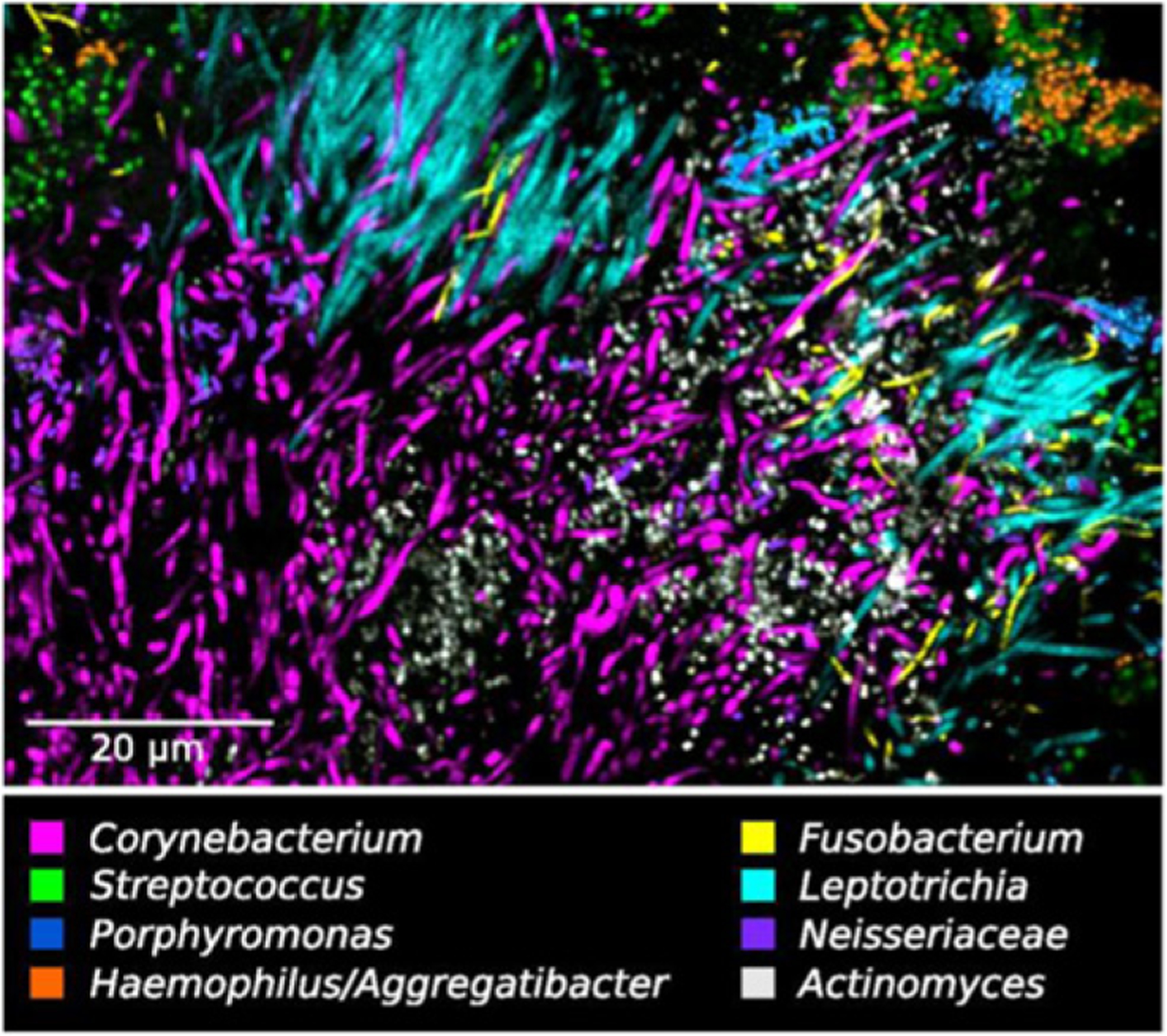
Oral biofilms imaged with CLASI-FISH. Different colors correspond to different bacterial genera. Image adapted from reference [147].

**Figure 24.**
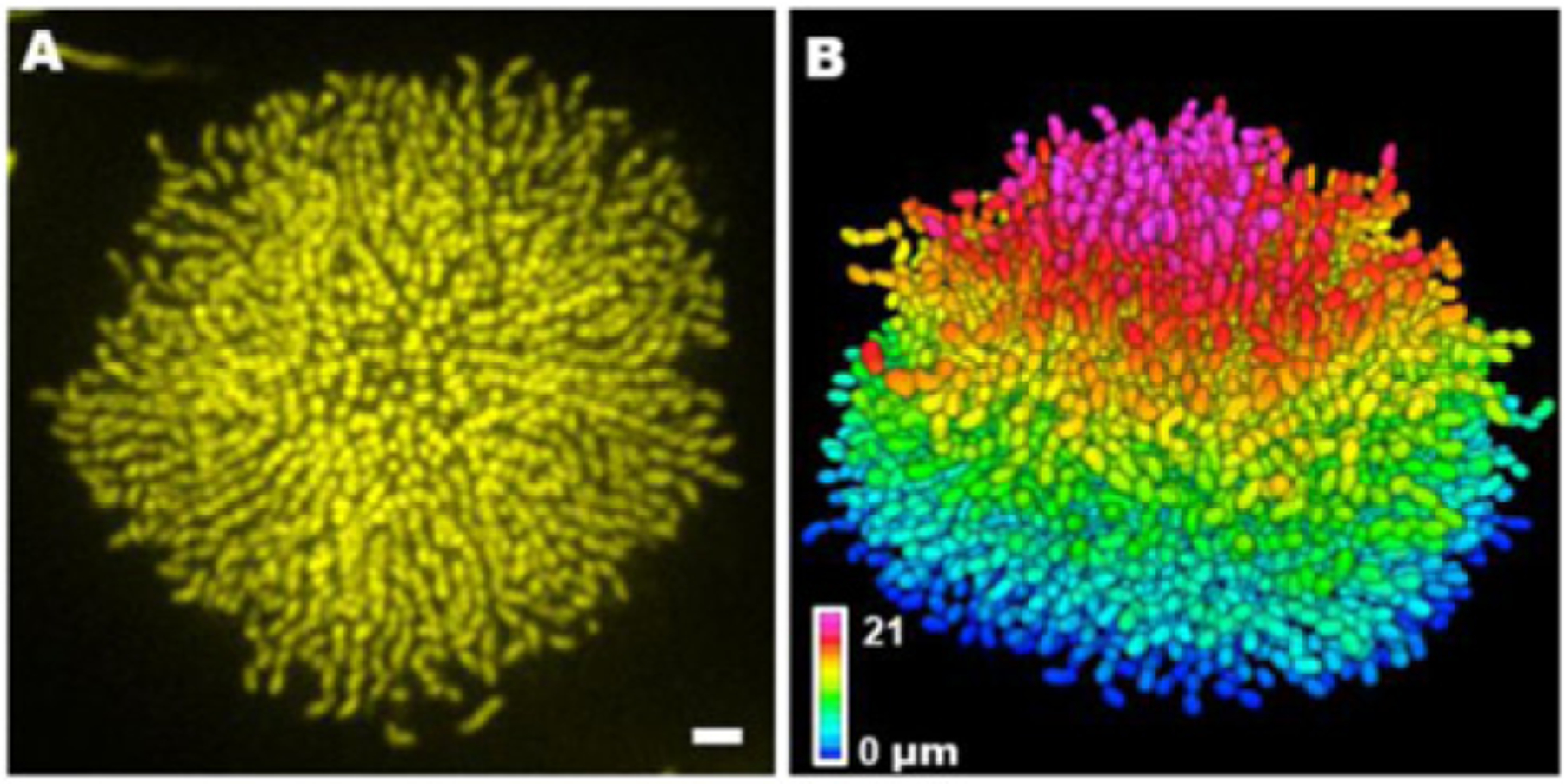
Single-cell live imaging of biofilms. (A) Cross-sectional image of the bottom cell layer of a growing *V. cholerae* biofilm cluster at 18 h and (B) the corresponding segmented image with color-coding according to *z* position. Scale bar: 3 *μ*m. Images adapted from reference [150].

**Figure 25.**
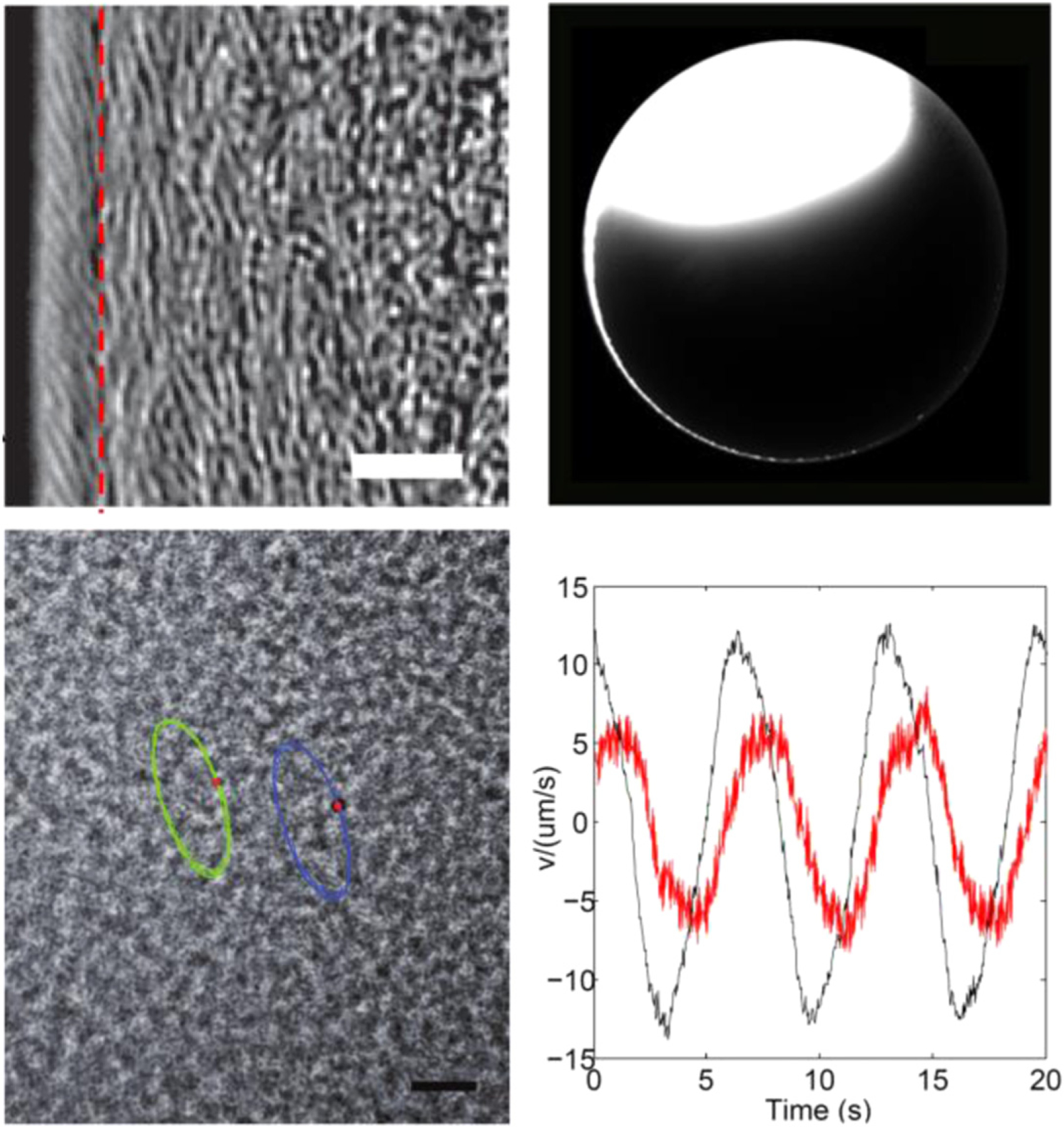
Spatial and temporal self-organization in motile bacterial populations. (a) Self-organization of two adjacent motile rings at the edge of a *Proteus mirabilis* colony. The outer and inner motile rings are located to the left and right of the red dashed line, respectively. Scale bar, 10 *μ*m. (b) Long-range, colony-scale directed transport along the inner motile ring of a *P. mirabilis* colony as demonstrated by the rapid flow of fluorescent microspheres around the colony edge in a counterclockwise manner. Scale bar, 500 *μ*m. (c) Two silicone oil tracers in an *E. coli* swarm moved in elliptical trajectories, reflecting collective oscillatory motion of cells in the swarm. Scale bar, 20 *μ*m. (b) Orthogonal components of collective cellular velocity as a function of time in a swarm undergoing collective oscillation. Panels (a) and (b) are adapted from reference [160] and (c) and (d) from reference [161], with the publishers’ permission.

**Figure 26.**
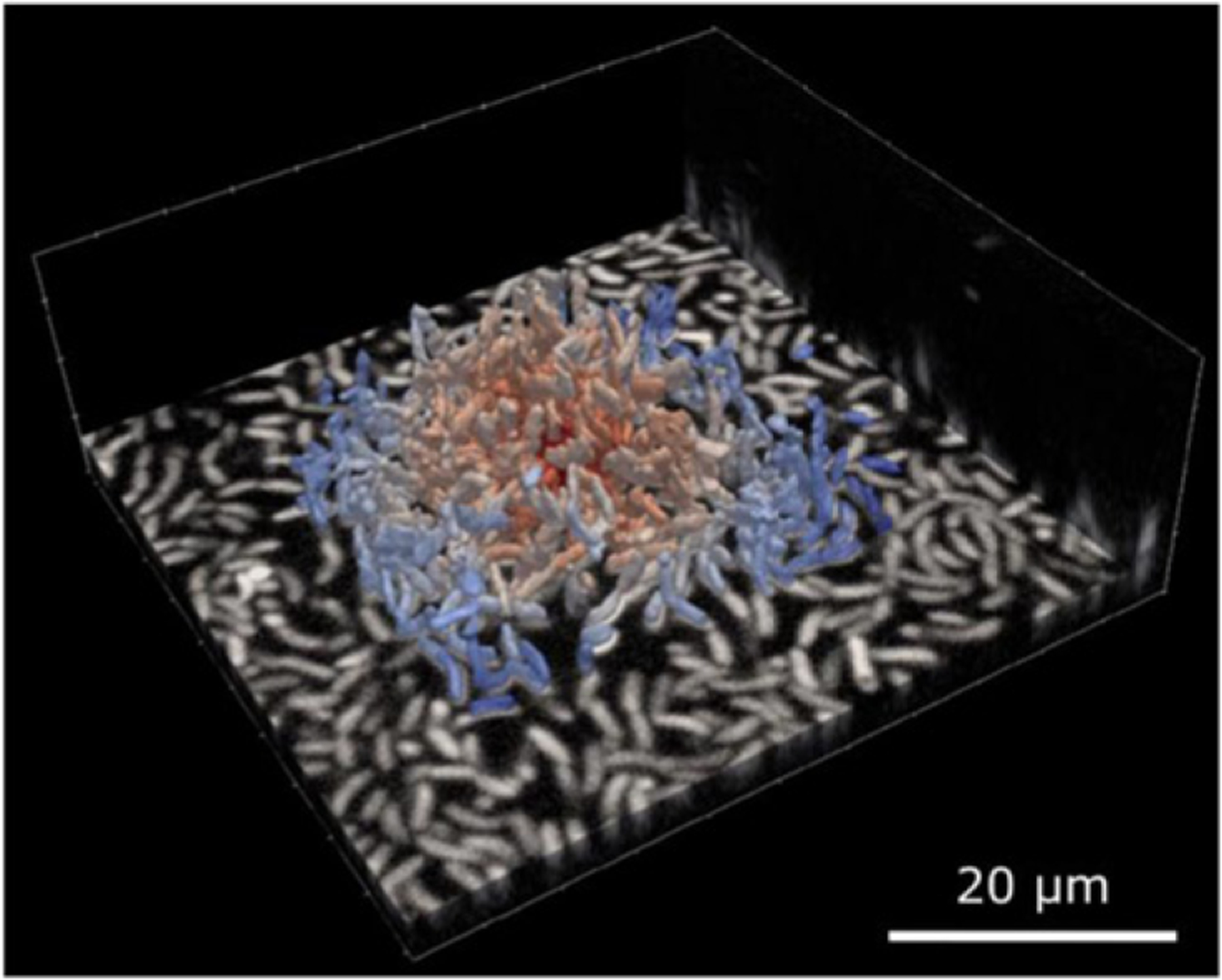
Single-cell-level data acquired during biofilm growth of *V. cholerae*, illustrated by raw confocal microscopy data (bottom layer) from which all individual cell outlines are segmented in three dimensions, and analyzed using BiofilmQ [172]. Each segmented cell is colored according to the distance from the center of the biofilm.

## References

[1] Chawla R, Gupta R, Lele TP and Lele PP 2020 A skeptic’s guide to bacterial mechanosensing J. Mol. Biol 432 523 31629771 10.1016/j.jmb.2019.09.004 PMC7002054

[2] Belas R 2014 Biofilms, flagella, and mechanosensing of surfaces by bacteria Trends Microbiol 22 517–27 24894628 10.1016/j.tim.2014.05.002

[3] Lele PP, Hosu BG and Berg HC 2013 Dynamics of mechanosensing in the bacterial flagellar motor Proc. Natl Acad. Sci 110 11839–44 23818629 10.1073/pnas.1305885110 PMC3718179

[4] Chawla R, Ford KM and Lele PP 2017 Torque, but not FliL, regulates mechanosensitive flagellar motor-function Sci. Rep 7 5565 28717192 10.1038/s41598-017-05521-8 PMC5514156

[5] McCarter L, Hilmen M and Silverman M 1988 Flagellar dynamometer controls swarmer cell differentiation of V. parahaemolyticus Cell 54 345–51 3396074 10.1016/0092-8674(88)90197-3

[6] Hug I, Deshpande S, Sprecher KS, Pfohl T and Jenal U 2017 Second messenger-mediated tactile response by a bacterial rotary motor Science 358 531–4 29074777 10.1126/science.aan5353

[7] Diethmaier C, Chawla R, Canzoneri A, Kearns DB, Lele PP and Dubnau D 2017 Viscous drag on the flagellum activates Bacillus subtilisentry into the K-state Mol. Microbiol 106 367–80 28800172 10.1111/mmi.13770 PMC5653444

[8] Waters CM and Bassler BL 2005 Quorum sensing: cell-to-cell communication in bacteria Annu. Rev. Cell Dev. Biol 21 319–46 16212498 10.1146/annurev.cellbio.21.012704.131001

[9] Berg HC and Purcell EM 1977 Physics of chemoreception Biophys. J 20 193–219 911982 10.1016/S0006-3495(77)85544-6 PMC1473391

[10] Qi LS, Larson MH, Gilbert LA, Doudna JA, Weissman JS, Arkin AP and Lim WA 2013 Repurposing CRISPR as an RNA-guided platform for sequence-specific control of gene expression Cell 152 1173–83 23452860 10.1016/j.cell.2013.02.022 PMC3664290

[11] Nan B and Zusman DR 2016 Novel mechanisms power bacterial gliding motility Mol. Microbiol 101 186–93 27028358 10.1111/mmi.13389 PMC5008027

[12] Faure LM 2016 The mechanism of force transmission at bacterial focal adhesion complexes Nature 539 530–5 27749817 10.1038/nature20121 PMC5465867

[13] Fu G, Bandaria JN, Le Gall AV, Fan X, Yildiz A, Mignot T, Zusman DR and Nan B 2018 MotAB-like machinery drives the movement of MreB filaments during bacterial gliding motility Proc. Natl Acad. Sci. USA 115 2484–9 29463706 10.1073/pnas.1716441115 PMC5877941

[14] Nan B, Chen J, Neu JC, Berry RM, Oster G and Zusman DR 2011 Myxobacteria gliding motility requires cytoskeleton rotation powered by proton motive force Proc. Natl Acad. Sci 108 2498–503 21248229 10.1073/pnas.1018556108 PMC3038734

[15] Treuner-Lange A 2015 The small G-protein MglA connects to the MreB actin cytoskeleton at bacterial focal adhesions J. Cell Biol 210 243–56 26169353 10.1083/jcb.201412047 PMC4508894

[16] Nan B, Bandaria JN, Moghtaderi A, Sun I-H, Yildiz A and Zusman DR 2013 Flagella stator homologs function as motors for myxobacterial gliding motility by moving in helical trajectories Proc. Natl Acad. Sci 110 E1508–13 23576734 10.1073/pnas.1219982110 PMC3631661

[17] Nan B 2017 Bacterial gliding motility: rolling out a consensus model Curr. Biol 27 R154–6 28222296 10.1016/j.cub.2016.12.035

[18] Wartel M (2013). A versatile class of cell surface directional motors gives rise to gliding motility and sporulation in Myxococcus xanthus. PLoS Biol.

[19] Zhang H, Mulholland GA, Seef S, Zhu S, Liu J, Mignot T and Nan B 2020 Establishing rod shape from spherical, peptidoglycan-deficient bacterial spores Proc. Natl Acad. Sci. USA 117 14444–52 32513721 10.1073/pnas.2001384117 PMC7321990

[20] Jakobczak B, Keilberg D, Wuichet K and Søgaard-Andersen L 2015 Contact- and protein transfer-dependent stimulation of assembly of the gliding motility machinery in Myxococcus xanthus PLoS Genet 11 e1005341 26132848 10.1371/journal.pgen.1005341 PMC4488436

[21] Talà L, Fineberg A, Kukura P and Persat A 2019 Pseudomonas aeruginosa orchestrates twitching motility by sequential control of type IV pili movements Nat. Microbiol 4 774–80 30804544 10.1038/s41564-019-0378-9 PMC6522360

[22] Chang Y-W, Rettberg LA, Treuner-Lange A, Iwasa J, Søgaard-Andersen L and Jensen GJ 2016 Architecture of the type IVa pilus machine Science 351 aad2001 26965631 10.1126/science.aad2001 PMC5929464

[23] Wang F, Coureuil M, Osinski T, Orlova A, Altindal T, Gesbert G, Nassif X, Egelman EH and Craig L 2017 Cryoelectron microscopy reconstructions of the Pseudomonas aeruginosa and Neisseria gonorrhoeae type iv pili at sub-nanometer resolution Structure 25 1423–35 28877506 10.1016/j.str.2017.07.016 PMC8189185

[24] Persat A, Inclan YF, Engel JN, Stone HA and Gitai Z 2015 Type IV pili mechanochemically regulate virulence factors in Pseudomonas aeruginosa Proc. Natl Acad. Sci. USA 112 7563–8 26041805 10.1073/pnas.1502025112 PMC4475988

[25] Lee CK 2018 Multigenerational memory and adaptive adhesion in early bacterial biofilm communities Proc. Natl Acad. Sci. USA 115 4471–6 29559526 10.1073/pnas.1720071115 PMC5924909

[26] Ellison CK 2017 Obstruction of pilus retraction stimulates bacterial surface sensing Science 358 535–8 29074778 10.1126/science.aan5706 PMC5805138

[27] Adams DW, Stutzmann S, Stoudmann C and Blokesch M 2019 DNA-uptake pili of Vibrio cholerae are required for chitin colonization and capable of kin recognition via sequence-specific self-interaction Nat. Microbiol 4 1545–57 31182799 10.1038/s41564-019-0479-5 PMC6708440

[28] Koch MD, Fei C, Wingreen NS, Shaevitz JW and Gitai Z 2021 Competitive binding of independent extension and retraction motors explains the quantitative dynamics of type IV pili Proc. Natl. Acad. Sci 118 e2014926118 33593905 10.1073/pnas.2014926118 PMC7923367

[29] Maier B, Potter L, So M, Seifert HS and Sheetz MP 2002 Single pilus motor forces exceed 100 pN Proc. Natl Acad. Sci 99 16012–7 12446837 10.1073/pnas.242523299 PMC138556

[30] Beaussart A, Baker AE, Kuchma SL, El-Kirat-Chatel S, O’Toole GA and Dufrêne YF 2014 Nanoscale Adhesion forces of Pseudomonas aeruginosa type IV pili ACS Nano 8 10723–33 25286300 10.1021/nn5044383 PMC4212785

[31] Dufrêne YF and Persat A 2020 Mechanomicrobiology: how bacteria sense and respond to forces Nat. Rev. Microbiol 18 227–40 31959911 10.1038/s41579-019-0314-2

[32] Mitchison TJ and Cramer LP 1996 Actin-based cell motility and cell locomotion Cell 84 371–9 8608590 10.1016/s0092-8674(00)81281-7

[33] Denis K 2019 Targeting type IV pili as an antivirulence strategy against invasive meningococcal disease Nat. Microbiol 4 972–84 30911127 10.1038/s41564-019-0395-8

[34] Hampton HG, Watson BNJ and Fineran PC 2020 The arms race between bacteria and their phage foes Nature 577 327–36 31942051 10.1038/s41586-019-1894-8

[35] Bru J-L, Rawson B, Trinh C, Whiteson K, Høyland-Kroghsbo NM and Siryaporn A 2019 PQS produced by the Pseudomonas aeruginosa stress response repels swarms away from bacteriophage and antibiotics J. Bacteriol 201 e00383 31451543 10.1128/JB.00383-19 PMC6832071

[36] Testa S, Berger S, Piccardi P, Oechslin F, Resch G and Mitri S 2019 Spatial structure affects phage efficacy in infecting dual-strain biofilms of Pseudomonas aeruginosa Commun. Biol 2 405 31701033 10.1038/s42003-019-0633-x PMC6828766

[37] Díaz-Pascual F (2019). Breakdown of Vibrio cholerae biofilm architecture induced by antibiotics disrupts community barrier function. Nat. Microbiol.

[38] Miller C, Kong J, Tran TT, Arias CA, Saxer G and Shamoo Y 2013 Adaptation of Enterococcus faecalis to daptomycin reveals an ordered progression to resistance Antimicrob. Agents Chemother 57 5373–83 10.1128/AAC.01473-13 PMC3811304 23959318

[39] Walters MC, Roe F, Bugnicourt A, Franklin MJ and Stewart PS 2003 Contributions of antibiotic penetration, oxygen limitation, and low metabolic activity to tolerance of Pseudomonas aeruginosa biofilms to ciprofloxacin and tobramycin Antimicrob. Agents Chemother 47 317–23 10.1128/AAC.47.1.317-323.2003 PMC148957 12499208

[40] Perinbam K, Chacko JV, Kannan A, Digman MA and Siryaporn A 2020 A shift in central metabolism accompanies virulence activation in Pseudomonas aeruginosa mBio 11 e02730–18 32156820 10.1128/mBio.02730-18 PMC7064766

[41] Huh D, Hamilton GA and Ingber DE 2011 From 3D cell culture to organs-on-chips Trends Cell Biol 21 745–54 10.1016/j.tcb.2011.09.005 PMC4386065 22033488

[42] Park SE, Georgescu A and Huh D 2019 Organoids-on-a-chip Science 364 960–5 31171693 10.1126/science.aaw7894 PMC7764943

[43] Gon S, Kumar K-N, Nüsslein K and Santore MM 2012 How bacteria adhere to brushy PEG surfaces: clinging to flaws and compressing the brush Macromolecules 45 8373–81 23148127 10.1021/ma300981r PMC3494094

[44] Sharma S 2016 Subnanometric roughness affects the deposition and mobile adhesion of Escherichia coli on silanized glass surfaces Langmuir 32 5422–33 27158837 10.1021/acs.langmuir.6b00883

[45] Friedlander RS, Vlamakis H, Kim P, Khan M, Kolter R and Aizenberg J 2013 Bacterial flagella explore microscale hummocks and hollows to increase adhesion Proc. Natl Acad. Sci 110 5624–9 23509269 10.1073/pnas.1219662110 PMC3619351

[46] Rosenberg M 2006 Microbial adhesion to hydrocarbons: twenty-five years of doing MATH FEMS Microbiol. Lett 262 129–34 10.1111/j.1574-6968.2006.00291.x 16923066

[47] Abbasnezhad H, Gray M and Foght JM 2011 Influence of adhesion on aerobic biodegradation and bioremediation of liquid hydrocarbons Appl. Microbiol. Biotechnol 92 653–75 10.1007/s00253-011-3589-4 21964551

[48] Deng J, Molaei M, Chisholm NG and Stebe KJ 2020 Motile bacteria at oil–water interfaces: Pseudomonas aeruginosa Langmuir 36 6888–902 32097012 10.1021/acs.langmuir.9b03578

[49] Dewangan NK and Conrad JC 2020 Bacterial motility enhances adhesion to oil droplets Soft Matter 16 8237–44 32935718 10.1039/d0sm00944j

[50] Zhao K, Tseng BS, Beckerman B, Jin F, Gibiansky ML, Harrison JJ, Luijten E, Parsek MR and Wong GCL 2013 Psl trails guide exploration and microcolony formation in Pseudomonas aeruginosa biofilms Nature 497 388–91 23657259 10.1038/nature12155 PMC4109411

[51] Sharma S and Conrad JC 2014 Attachment from flow of Escherichia coli bacteria onto silanized glass substrates Langmuir 30 11147–55 25153944 10.1021/la502313y

[52] Song L, Sjollema J, Sharma PK, Kaper HJ, van der Mei HC and Busscher HJ 2014 Nanoscopic vibrations of bacteria with different cell–wall properties adhering to surfaces under flow and static conditions ACS Nano 8 8457–67 25025495 10.1021/nn5030253

[53] Vissers T (2018). Bacteria as living patchy colloids: phenotypic heterogeneity in surface adhesion. Sci. Adv.

[54] Xia A, Yang S, Zhang R, Ni L, Xing X and Jin F 2019 Imaging the separation distance between the attached bacterial cells and the surface with a total internal reflection dark-field microscope Langmuir 35 8860–6 31194567 10.1021/acs.langmuir.9b01378

[55] McLay RB, Nguyen HN, Jaimes-Lizcano YA, Dewangan NK, Alexandrova S, Rodrigues DF, Cirino PC and Conrad JC 2018 Level of fimbriation alters the adhesion of Escherichia coli bacteria to interfaces Langmuir 34 1133–42 28976770 10.1021/acs.langmuir.7b02447

[56] Wang A, Garmann RF and Manoharan VN 2016 Tracking E. coli runs and tumbles with scattering solutions and digital holographic microscopy Opt. Express 24 23719–25 27828208 10.1364/OE.24.023719

[57] Azam F and Malfatti F 2007 Microbial structuring of marine ecosystems Nat. Rev. Microbiol 5 782–91 17853906 10.1038/nrmicro1747

[58] Kiørboe T, Grossart HP, Ploug H and Tang K 2002 Mechanisms and rates of bacterial colonization of sinking aggregates Appl. Environ. Microbiol 68 3996–4006 12147501 10.1128/AEM.68.8.3996-4006.2002 PMC124032

[59] Cram JA, Weber T, Leung SW, McDonnell AMP, Liang J-H and Deutsch C 2018 The role of particle size, ballast, temperature, and oxygen in the sinking flux to the deep sea Global Biogeochem. Cycles 32 858–76

[60] Enke TN, Datta MS, Schwartzman J, Cermak N, Schmitz D, Barrere J, Pascual-García A and Cordero OX 2019 Modular assembly of polysaccharide-degrading marine microbial communities Curr. Biol 29 1528–35 31031118 10.1016/j.cub.2019.03.047

[61] Dang H and Lovell CR 2016 Microbial surface colonization and biofilm development in marine environments Microbiol. Mol. Biol. Rev 80 91–138 26700108 10.1128/MMBR.00037-15 PMC4711185

[62] Yawata Y, Carrara F, Menolascina F and Stocker R 2020 Constrained optimal foraging by marine bacterioplankton on particulate organic matter Proc. Natl Acad. Sci. USA 117 25571–9 32973087 10.1073/pnas.2012443117 PMC7568300

[63] Yan J, Nadell CD and Bassler BL 2017 Environmental fluctuation governs selection for plasticity in biofilm production ISME J 11 1569–77 28338673 10.1038/ismej.2017.33 PMC5520152

[64] Bianchi D, Weber TS, Kiko R and Deutsch C 2018 Global niche of marine anaerobic metabolisms expanded by particle microenvironments Nat. Geosci 11 263–8

[65] Zhang W (2019). Marine biofilms constitute a bank of hidden microbial diversity and functional potential. Nat. Commun.

[66] Lambert BS 2017 A microfluidics-based in situ chemotaxis assay to study the behaviour of aquatic microbial communities Nat. Microbiol 2 1344–9 28848238 10.1038/s41564-017-0010-9

[67] Burrows LL 2012 Pseudomonas aeruginosa twitching motility: type IV pili in action Annu. Rev. Microbiol 66 493–520 22746331 10.1146/annurev-micro-092611-150055

[68] Snyder RA, Ellison CK, Severin GB, Whitfield GB, Waters CM and Brun YV 2020 Surface sensing stimulates cellular differentiation in Caulobacter crescentus Proc. Natl Acad. Sci. USA 117 17984 32661164 10.1073/pnas.1920291117 PMC7395532

[69] Luo Y, Zhao K, Baker AE, Kuchma SL, Coggan KA, Wolfgang MC, Wong GCL and O’Toole GA 2015 A hierarchical cascade of second messengers regulates Pseudomonas aeruginosa surface behaviors mBio 6 e02456–14 25626906 10.1128/mBio.02456-14 PMC4324313

[70] Siryaporn A, Kuchma SL, O’Toole GA and Gitai Z 2014 Surface attachment induces Pseudomonas aeruginosa virulence Proc. Natl Acad. Sci. USA 111 16860–5 25385640 10.1073/pnas.1415712111 PMC4250119

[71] Medico LD, Cerletti D, Schächle P, Christen M and Christen B 2020 The type IV pilin PilA couples surface attachment and cell-cycle initiation in Caulobacter crescentus Proc. Natl Acad. Sci. USA 117 9546 32295877 10.1073/pnas.1920143117 PMC7196804

[72] Armbruster CR (2019). Heterogeneity in surface sensing suggests a division of labor in Pseudomonas aeruginosa populations. eLife.

[73] Ellison CK, Dalia TN, Vidal Ceballos A, Wang JC-Y, Biais N, Brun YV and Dalia AB 2018 Retraction of DNA-bound type IV competence pili initiates DNA uptake during natural transformation in Vibrio cholerae Nat. Microbiol 3 773–80 29891864 10.1038/s41564-018-0174-y PMC6582970

[74] Ellison CK, Dalia TN, Dalia AB and Brun YV 2019 Real-time microscopy and physical perturbation of bacterial pili using maleimide-conjugated molecules Nat. Protocols 14 1803–19 31028374 10.1038/s41596-019-0162-6 PMC7461830

[75] Cairns LS, Marlow VL, Bissett E, Ostrowski A and Stanley-Wall NR 2013 A mechanical signal transmitted by the flagellum controls signalling in Bacillus subtilis Mol. Microbiol 90 6–21 23888912 10.1111/mmi.12342 PMC3963450

[76] Laventie B-J 2019 A surface-induced asymmetric program promotes tissue colonization by Pseudomonas aeruginosa Cell Host Microbe 25 140–52 30581112 10.1016/j.chom.2018.11.008

[77] Utada AS, Bennett RR, Fong JCN, Gibiansky ML, Yildiz FH, Golestanian R and Wong GCL 2014 Vibrio cholerae use pili and flagella synergistically to effect motility switching and conditional surface attachment Nat. Commun 5 4913 25234699 10.1038/ncomms5913 PMC4420032

[78] Gelimson A, Zhao K, Lee CK, Kranz WT, Wong GCL and Golestanian R 2016 Multicellular self-organization of P. aeruginosa due to interactions with secreted trails Phys. Rev. Lett 117 178102 27824438 10.1103/PhysRevLett.117.178102

[79] Gibiansky ML (2010). Bacteria use type IV pili to walk upright and detach from surfaces: fig. 1. Science.

[80] Teschler JK, Zamorano-Sánchez D, Utada AS, Warner CJA, Wong GCL, Linington RG and Yildiz FH 2015 Living in the matrix: assembly and control of Vibrio cholerae biofilms Nat. Rev. Microbiol 13 255–68 25895940 10.1038/nrmicro3433 PMC4437738

[81] Conner JG, Zamorano-Sánchez D, Park JH, Sondermann H and Yildiz FH 2017 The ins and outs of cyclic di-GMP signaling in Vibrio cholerae Curr. Opin. Microbiol 36 20–9 28171809 10.1016/j.mib.2017.01.002 PMC5534393

[82] Jones CJ, Utada A, Davis KR, Thongsomboon W, Zamorano Sanchez D, Banakar V, Cegelski L, Wong GCL and Yildiz FH 2015 c-di-GMP regulates motile to sessile transition by modulating MshA pili biogenesis and near-surface motility behavior in Vibrio cholerae PLoS Pathog 11 1–27 10.1371/journal.ppat.1005068 PMC4624765 26505896

[83] Kitts G 2019 A conserved regulatory circuit controls large adhesins in Vibrio cholerae mBio 10 1–22 10.1128/mBio.02822-19 PMC6890996 31796544

[84] Floyd KA (2020). c-di-GMP modulates type IV MSHA pilus retraction and surface attachment in Vibrio cholerae. Nat. Commun.

[85] Zamorano-Sánchez D, Xian W, Lee CK, Salinas M, Thongsomboon W, Cegelski L, Wong GCL and Yildiz FH 2019 Functional specialization in Vibrio cholerae diguanylate cyclases: distinct modes of motility suppression and c-di-GMP production mBio 10 e00670–19 31015332 10.1128/mBio.00670-19 PMC6479008

[86] Zhou H, Zheng C, Su J, Chen B, Fu Y, Xie Y, Tang Q, Chou SH and He J 2016 Characterization of a natural triple-tandem c-di-GMP riboswitch and application of the riboswitch-based dual-fluorescence reporter Sci. Rep 6 20871 26892868 10.1038/srep20871 PMC4759541

[87] Christen M, Kulasekara HD, Christen B, Kulasekara BR, Hoffman LR and Miller SI 2010 Asymmetrical distribution of the second messenger c-di-GMP upon bacterial cell division Science 328 1295–7 20522779 10.1126/science.1188658 PMC3906730

[88] Caro F, Place NM and Mekalanos JJ 2019 Analysis of lipoprotein transport depletion in Vibrio cholerae using CRISPRi Proc. Natl Acad. Sci. USA 116 17013–22 31371515 10.1073/pnas.1906158116 PMC6708369

[89] Hengge R (2009). Principles of c-di-GMP signalling in bacteria. Nat. Rev. Microbiol.

[90] Ryu M-H and Gomelsky M 2014 Near-infrared light responsive synthetic c-di-GMP module for optogenetic applications ACS Synth. Biol 3 802–10 24926804 10.1021/sb400182x PMC4277780

[91] Barends TRM 2009 Structure and mechanism of a bacterial light-regulated cyclic nucleotide phosphodiesterase Nature 459 1015–8 19536266 10.1038/nature07966

[92] Cao Z, Livoti E, Losi A and Gärtner W 2010 A blue light-inducible phosphodiesterase activity in the cyanobacterium Synechococcus elongatus Photochem. Photobiol 86 606–11 10.1111/j.1751-1097.2010.00724.x 20408974

[93] Enomoto G, Ni-Ni-Win W, Narikawa R and Ikeuchi M 2015 Three cyanobacteriochromes work together to form a light color-sensitive input system for c-di-GMP signaling of cell aggregation Proc. Natl Acad. Sci. USA 112 8082 10.1073/pnas.1504228112 PMC4491779 26080423

[94] Ryu M-H, Fomicheva A, Moskvin OV and Gomelsky M 2017 Optogenetic module for dichromatic control of c-di-GMP signaling J. Bacteriol 199 e00014–17 28320886 10.1128/JB.00014-17 PMC5573075

[95] Neal L, Ryu M-H, Gomelsky M and Alexandre G 2017 Optogenetic manipulation of cyclic di-GMP (c-di-GMP) levels reveals the role of c-di-GMP in regulating aerotaxis receptor activity in Azospirillum brasilense J. Bacteriol 199 e00020–17 28264994 10.1128/JB.00020-17 PMC5573079

[96] Pu L, Yang S, Xia A and Jin F 2018 Optogenetics manipulation enables prevention of biofilm formation of engineered Pseudomonas aeruginosaon surfaces ACS Synth. Biol 7 200–8 29053252 10.1021/acssynbio.7b00273

[97] Huang Y, Xia A, Yang G and Jin F 2018 Bioprinting living biofilms through optogenetic manipulation ACS Synth. Biol 7 1195–200 29664610 10.1021/acssynbio.8b00003

[98] Shao J (2017). Smartphone-controlled optogenetically engineered cells enable semiautomatic glucose homeostasis in diabetic mice. Sci. Transl. Med.

[99] Whiteley M, Diggle SP and Greenberg EP 2017 Progress in and promise of bacterial quorum sensing research Nature 551 313–20 29144467 10.1038/nature24624 PMC5870893

[100] Dickey SW, Cheung GYC and Otto M 2017 Different drugs for bad bugs: antivirulence strategies in the age of antibiotic resistance Nat. Rev. Drug Discovery 16 457–71 28337021 10.1038/nrd.2017.23 PMC11849574

[101] D’Angelo F 2018 Identification of FDA-approved drugs as antivirulence agents targeting the pqs quorum-sensing system of Pseudomonas aeruginosa Antimicrob. Agents Chemother 62 e01296–18 10.1128/AAC.01296-18 PMC6201120 30201815

[102] Mashburn LM and Whiteley M 2005 Membrane vesicles traffic signals and facilitate group activities in a prokaryote Nature 437 422–5 16163359 10.1038/nature03925

[103] Brameyer S 2018 Outer membrane vesicles facilitate trafficking of the hydrophobic signaling molecule CAI-1 between Vibrio harveyi cells J. Bacteriol 200 e00740–17 29555694 10.1128/JB.00740-17 PMC6040191

[104] Toyofuku M, Morinaga K, Hashimoto Y, Uhl J, Shimamura H, Inaba H, Schmitt-Kopplin P, Eberl L and Nomura N 2017 Membrane vesicle-mediated bacterial communication ISME J 11 1504–9 28282039 10.1038/ismej.2017.13 PMC5437348

[105] Morinaga K, Yamamoto T, Nomura N and Toyofuku M 2018 Paracoccus denitrificans can utilize various long-chain N-acyl homoserine lactones and sequester them in membrane vesicles Environ. Microbiol. Rep 10 651–4 29968275 10.1111/1758-2229.12674

[106] Toyofuku M, Roschitzki B, Riedel K and Eberl L 2012 Identification of proteins associated with the Pseudomonas aeruginosa biofilm extracellular matrix J. Proteome Res 11 4906–15 22909304 10.1021/pr300395j

[107] Schooling SR and Beveridge TJ 2006 Membrane vesicles: an overlooked component of the matrices of biofilms J. Bacteriol 188 5945–57 16885463 10.1128/JB.00257-06 PMC1540058

[108] Turnbull L (2016). Explosive cell lysis as a mechanism for the biogenesis of bacterial membrane vesicles and biofilms. Nat. Commun.

[109] Toyofuku M, Nomura N and Eberl L 2019 Types and origins of bacterial membrane vesicles Nat. Rev. Microbiol 17 13–24 30397270 10.1038/s41579-018-0112-2

[110] Chong GW, Karbelkar AA and El-naggar MY 2018 Nature’s conductors: what can microbial multi-heme cytochromes teach us about electron transport and biological energy conversion? Curr. Opin. Chem. Biol 47 7–17 30015234 10.1016/j.cbpa.2018.06.007

[111] Zacharoff LA and El-Naggar MY 2017 Redox conduction in biofilms: from respiration to living electronics Curr. Opin. Electrochem 4 182

[112] Wang F 2019 Structure of microbial nanowires reveals stacked hemes that transport electrons over micrometers Cell 177 361–9 30951668 10.1016/j.cell.2019.03.029 PMC6720112

[113] Yee MO, Snoeyenbos-West OL, Thamdrup B and Ottosen LDM 2019 Extracellular electron uptake by two methanosarcina species Front. Energy Res 7 29

[114] Røder HL, Olsen NMC, Whiteley M and Burmølle M 2020 Unraveling interspecies interactions across heterogeneities in complex biofilm communities Environ. Microbiol 22 5 31637837 10.1111/1462-2920.14834

[115] Light SH, Su L, Rivera-Lugo R, Cornejo JA, Louie A, Iavarone AT, Ajo-Franklin CM and Portnoy DA 2018 A flavin-based extracellular electron transfer mechanism in diverse Gram-positive bacteria Nature 562 140–4 30209391 10.1038/s41586-018-0498-z PMC6221200

[116] Yates MD (2016). Toward understanding long-distance extracellular electron transport in an electroautotrophic microbial community. Energy Environ. Sci.

[117] Yuan S (2013). A photometric high-throughput method for identification of electrochemically active bacteria using a WO3 nanocluster probe. Sci. Rep.

[118] Hong G, Yang X, Zhou T and Lieber CM 2018 Mesh electronics: a new paradigm for tissue-like brain probes Curr. Opin. Neurobiol 50 33–41 29202327 10.1016/j.conb.2017.11.007 PMC5984112

[119] Lee DD, Prindle A, Liu J and Süel GM 2017 SnapShot: electrochemical communication in biofilms Cell 170 214 28666120 10.1016/j.cell.2017.06.026

[120] Malvankar NS 2011 Tunable metallic-like conductivity in microbial nanowire networks Nat. Nanotech 6 573–9 10.1038/nnano.2011.119 21822253

[121] Qian F and Li Y 2011 A natural source of nanowires Nat. Nanotech 6 538–9 10.1038/nnano.2011.148 21897384

[122] Yalcin SE 2020 Electric field stimulates production of highly conductive microbial OmcZ nanowires Nat. Chem. Biol 16 1136–42 32807967 10.1038/s41589-020-0623-9 PMC7502555

[123] Holmes DE 2006 Microarray and genetic analysis of electron transfer to electrodes in Geobacter sulfurreducens Environ. Microbiol 8 1805–15 16958761 10.1111/j.1462-2920.2006.01065.x

[124] Nevin KP (2009). Anode biofilm transcriptomics reveals outer surface components essential for high density current production in Geobacter sulfurreducens fuel cells. PLoS One.

[125] Leang C, Malvankar NS, Franks AE, Nevin KP and Lovley DR 2013 Engineering Geobacter sulfurreducens to produce a highly cohesive conductive matrix with enhanced capacity for current production Energy Environ. Sci 6 1901–8

[126] Chadwick GL, Jiménez Otero F, Gralnick JA, Bond DR and Orphan VJ 2019 NanoSIMS imaging reveals metabolic stratification within current-producing biofilms Proc. Natl Acad. Sci. USA 116 20716–24 31548422 10.1073/pnas.1912498116 PMC6789570

[127] Yalcin SE and Malvankar NS 2020 The blind men and the filament: understanding structures and functions of microbial nanowires Curr. Opin. Chem. Biol 59 193–201 33070100 10.1016/j.cbpa.2020.08.004 PMC7736336

[128] Shipps C (2020). Intrinsic electronic conductivity of individual atomically resolved amyloid crystals reveals micrometer-long hole hopping via tyrosines. Proc. Natl Acad. Sci. USA.

[129] Belianinov A, Ievlev AV, Lorenz M, Borodinov N, Doughty B, Kalinin SV, Fernández FM and Ovchinnikova OS 2018 Correlated materials characterization via multimodal chemical and functional imaging ACS Nano 12 11798–818 30422627 10.1021/acsnano.8b07292 PMC9850281

[130] Scheer E 2014 Nat. Nanotechnol 9 1012–217 25466536 10.1038/nnano.2014.293

[131] Malvankar NS, Yalcin SE, Tuominen MT and Lovley DR 2014 Visualization of charge propagation along individual pili proteins using ambient electrostatic force microscopy Nat. Nanotech 9 1012–7 10.1038/nnano.2014.236 25326694

[132] Zdrojek M, Mélin T, Diesinger H, Stíevenard D, Gebicki W and Adamowicz L 2006 Phys. Rev. Lett 96 039703 16486790 10.1103/PhysRevLett.96.039703

[133] Yalcin SE, Legg BA, Yesşilbaş M, Malvankar NS and Boily J-F 2020 Direct observation of anisotropic growth of water films on minerals driven by defects and surface tension Sci. Adv 6 eaaz9708 32832658 10.1126/sciadv.aaz9708 PMC7439304

[134] Stewart PS and Franklin MJ 2008 Physiological heterogeneity in biofilms Nat. Rev. Microbiol 6 199–210 18264116 10.1038/nrmicro1838

[135] Nguyen D 2011 Active starvation responses mediate antibiotic tolerance in biofilms and nutrient-limited bacteria Science 334 982–6 22096200 10.1126/science.1211037 PMC4046891

[136] Lopatkin AJ, Stokes JM, Zheng EJ, Yang JH, Takahashi MK, You L and Collins JJ 2019 Bacterial metabolic state more accurately predicts antibiotic lethality than growth rate Nat. Microbiol 4 2109 31451773 10.1038/s41564-019-0536-0 PMC6879803

[137] Balaban NQ (2019). Publisher correction: definitions and guidelines for research on antibiotic persistence. Nat. Rev. Microbiol.

[138] Levin-Reisman I, Brauner A, Ronin I and Balaban NQ 2019 Epistasis between antibiotic tolerance, persistence, and resistance mutations Proc. Natl Acad. Sci. USA 116 14734–9 31262806 10.1073/pnas.1906169116 PMC6642377

[139] Chadwick GL, Jiménez Otero F, Gralnick JA, Bond DR and Orphan VJ 2019 NanoSIMS imaging reveals metabolic stratification within current-producing biofilms Proc. Natl Acad. Sci. USA 116 20716 31548422 10.1073/pnas.1912498116 PMC6789570

[140] Liu J, Prindle A, Humphries J, Gabalda-Sagarra M, Asally M, Lee D-y D, Ly S, Garcia-Ojalvo J and Süel GM 2015 Metabolic co-dependence gives rise to collective oscillations within biofilms Nature 523 550–4 26200335 10.1038/nature14660 PMC4862617

[141] Kholodenko BN 2006 Cell-signalling dynamics in time and space Nat. Rev. Mol. Cell Biol 7 165–76 16482094 10.1038/nrm1838 PMC1679905

[142] DePas WH, Starwalt-Lee R, Van Sambeek L, Ravindra Kumar S, Gradinaru V and Newman DK 2016 Exposing the three-dimensional biogeography and metabolic states of pathogens in cystic fibrosis sputum via hydrogel embedding, clearing, and rRNA labeling mBio 7 e00796–16 27677788 10.1128/mBio.00796-16 PMC5040109

[143] McLean JS, Ona ON and Majors PD 2008 Correlated biofilm imaging, transport and metabolism measurements via combined nuclear magnetic resonance and confocal microscopy ISME J 2 121–31 18253132 10.1038/ismej.2007.107 PMC4454505

[144] Schiessl KT, Hu FH, Jo J, Nazia SZ, Wang B, Price-Whelan A, Min W and Dietrich LEP 2019 Phenazine production promotes antibiotic tolerance and metabolic heterogeneity in Pseudomonas aeruginosa biofilms Nat. Commun 10 762 30770834 10.1038/s41467-019-08733-w PMC6377615

[145] Bhattacharjee A, Datta R, Gratton E and Hochbaum AI 2017 Metabolic fingerprinting of bacteria by fluorescence lifetime imaging microscopy Sci. Rep 7 3743 28623341 10.1038/s41598-017-04032-w PMC5473825

[146] Ghannoum M, Parsek M, Whiteley M and Mukherjee P 2015 Microbial Biofilms (ASM Press)

[147] Mark Welch JL, Rossetti BJ, Rieken CW, Dewhirst FE and Borisy GG 2016 Biogeography of a human oral microbiome at the micron scale Proc. Natl Acad. Sci. USA 113 E791–800 26811460 10.1073/pnas.1522149113 PMC4760785

[148] Stewart EJ, Satorius AE, Younger JG and Solomon MJ 2013 Role of environmental and antibiotic stress on Staphylococcus epidermidis biofilm microstructure Langmuir 29 7017–24 23688391 10.1021/la401322k PMC4144346

[149] Drescher K, Dunkel J, Nadell CD, van Teeffelen S, Grnja I, Wingreen NS, Stone HA and Bassler BL 2016 Architectural transitions in Vibrio cholerae biofilms at single-cell resolution Proc. Natl Acad. Sci. USA 113 E2066–72 26933214 10.1073/pnas.1601702113 PMC4833255

[150] Yan J, Sharo AG, Stone HA, Wingreen NS and Bassler BL 2016 Vibrio cholerae biofilm growth program and architecture revealed by single-cell live imaging Proc. Natl Acad. Sci. USA 113 E5337–43 27555592 10.1073/pnas.1611494113 PMC5018804

[151] Beroz F, Yan J, Meir Y, Sabass B, Stone HA, Bassler BL and Wingreen NS 2018 Verticalization of bacterial biofilms Nat. Phys 14 954–60 30906420 10.1038/s41567-018-0170-4 PMC6426328

[152] Keller PJ, Schmidt AD, Wittbrodt J and Stelzer EHK 2018 Reconstruction of zebrafish early embryonic development by scanned light sheet microscopy Science 322 1065–9 10.1126/science.1162493 18845710

[153] Kumar A 2014 Dual-view plane illumination microscopy for rapid and spatially isotropic imaging Nat. Protocols 9 2555–73 25299154 10.1038/nprot.2014.172 PMC4386612

[154] Young KD 2006 The selective value of bacterial shape Microbiol. Mol. Biol. Rev 70 660–703 16959965 10.1128/MMBR.00001-06 PMC1594593

[155] Bjarnsholt T, Jensen PØ, Fiandaca MJ, Pedersen J, Hansen CR, Andersen CB, Pressler T, Givskov M and Høiby N 2009 Pseudomonas aeruginosa biofilms in the respiratory tract of cystic fibrosis patients Pediatr. Pulmonol 44 547–58 19418571 10.1002/ppul.21011

[156] Qin B, Fei C, Bridges AA, Mashruwala AA, Stone HA, Wingreen NS and Bassler BL 2020 Cell position fates and collective fountain flow in bacterial biofilms revealed by light-sheet microscopy Science 369 71 32527924 10.1126/science.abb8501 PMC7426073

[157] Butler MT, Wang Q and Harshey RM 2010 Cell density and mobility protect swarming bacteria against antibiotics Proc. Natl Acad. Sci. USA 107 3776–81 20133590 10.1073/pnas.0910934107 PMC2840483

[158] Zuo W and Wu Y 2020 Dynamic motility selection drives population segregation in a bacterial swarm Proc. Natl Acad. Sci. USA 117 4693–700 32060120 10.1073/pnas.1917789117 PMC7060710

[159] Shrivastava A, Patel VK, Tang Y, Yost SC, Dewhirst FE and Berg HC 2018 Cargo transport shapes the spatial organization of a microbial community Proc. Natl Acad. Sci. USA 115 8633–8 30082394 10.1073/pnas.1808966115 PMC6112710

[160] Xu H, Dauparas J, Das D, Lauga E and Wu Y 2019 Self-organization of swimmers drives long-range fluid transport in bacterial colonies Nat. Commun 10 1792 30996269 10.1038/s41467-019-09818-2 PMC6470179

[161] Chen C, Liu S, Shi X-q, Chaté H and Wu Y 2017 Weak synchronization and large-scale collective oscillation in dense bacterial suspensions Nature 542 210–4 28114301 10.1038/nature20817

[162] Cates ME and Tailleur J 2015 Motility-induced phase separation Annu. Rev. Condens. Matter Phys 6 219–44

[163] Liu G (2019). Self-driven phase transitions drive Myxococcus xanthus fruiting body formation. Phys. Rev. Lett.

[164] Schwarz-Linek J, Valeriani C, Cacciuto A, Cates ME, Marenduzzo D, Morozov AN and Poon WCK 2012 Phase separation and rotor self-assembly in active particle suspensions Proc. Natl Acad. Sci 109 4052–7 22392986 10.1073/pnas.1116334109 PMC3306685

[165] Cotter CR, Schüttler H-B, Igoshin OA and Shimkets LJ 2017 Data-driven modeling reveals cell behaviors controlling self-organization during Myxococcus xanthus development Proc. Natl Acad. Sci. USA 114 E4592–601 28533367 10.1073/pnas.1620981114 PMC5468666

[166] Möbius W and Laan L 2015 Physical and mathematical modeling in experimental papers Cell 163 1577–83 26687351 10.1016/j.cell.2015.12.006

[167] Nadell CD, Drescher K and Foster KR 2016 Spatial structure, cooperation and competition in biofilms Nat. Rev. Microbiol 14 589–600 27452230 10.1038/nrmicro.2016.84

[168] Stewart PS and Franklin MJ 2008 Physiological heterogeneity in biofilms Nat. Rev. Microbiol 6 199–210 18264116 10.1038/nrmicro1838

[169] Hartmann R, Singh PK, Pearce P, Mok R, Song B, Díaz-Pascual F, Dunkel J and Drescher K 2019 Emergence of three-dimensional order and structure in growing biofilms Nat. Phys 15 251–6 31156716 10.1038/s41567-018-0356-9 PMC6544526

[170] Pearce P (2019). Flow-induced symmetry breaking in growing bacterial biofilms. Phys. Rev. Lett.

[171] Díaz-Pascual F 2019 Breakdown of Vibrio cholerae biofilm architecture induced by antibiotics disrupts community barrier function Nat. Microbiol 4 2136–45 31659297 10.1038/s41564-019-0579-2 PMC6881181

[172] Hartmann R 2021 Quantitative image analysis of microbial communities with BiofilmQ Nat. Microbiol 6 151–6 33398098 10.1038/s41564-020-00817-4 PMC7840502

[173] Zhao H, Storey BD, Braatz RD and Bazant MZ 2020 Learning the physics of pattern formation from images Phys. Rev. Lett 124 060201 32109085 10.1103/PhysRevLett.124.060201

[174] Brunton SL, Proctor JL, Kutz JN and Bialek W 2016 Discovering governing equations from data by sparse identification of nonlinear dynamical systems Proc. Natl Acad. Sci. USA 113 3932–7 27035946 10.1073/pnas.1517384113 PMC4839439

[175] Skinner DJ, Song B, Jeckel H, Jelly E, Drescher K and Dunkel J 2021 Topological metric detects hidden order in disordered media Phys. Rev. Lett 126 048101 33576647 10.1103/PhysRevLett.126.048101

