# Supplementary material for: Roadmap on emerging concepts in the physical biology of bacterial biofilms: from surface sensing to community formation
Source: Phys Biol. Author manuscript; Available in PMC 2022 Jun 23. (PMC8506656; doi:10.1088/1478-3975/abdc0e)
Supplement: Related texts

# Research article

**Authors:** Jyot D Antani, Pushkar P Lele

**Affiliations:** Artie McFerrin Department of Chemical Engineering, Texas A & M University, College Station, TX 77843, United States of America

## 2. Role of bacterial flagella in surface sensing

### 2.1. Status

Bacterial motility and chemotaxis are virulence factors that facilitate host invasion. Motility is predominantly mediated by rotary flagella that propel a cell through viscous bulk fluids. The chemotaxis network modulates flagellar reversals to enable the bacterium to migrate in response to chemical gradients. Together, the two processes are crucial for motile bacteria in their search for favorable niches.

Once a motile bacterium reaches a suitable surface, it may transition from its planktonic state to a surface-associated state. The mechanisms of this transition are unknown but likely involve the sensing of surface adhesion by the bacterium and subsequent signal transduction—termed surface sensing. Surface sensing promotes the development of thriving microbial communities on surfaces, such as biofilms, which are adept at withstanding several environmental stressors including antibiotics.

Surface sensing is strongly influenced by the stiffness of the semi-solid or solid surface since it controls the strength of the mechanical load on an adherent bacterium. Changes in mechanical load, which arise due to the attachment of the cell to a surface, are detected through a process termed mechanosensing [1]. Mechanosensing modulates protein structure-function to regulate a myriad of bacterial functions. Although unlikely to be the only surface sensing strategy, mechanosensing is probably a widespread phenomenon in the bacterial kingdom.

Among the known mechanosensors, the bacterial flagella are prominent [2]. The rotation of individual flagellar filaments is powered by a transmembrane motor consisting of several proteins that form a rotor complex and a torque-generating stator complex containing multiple units. Adhesion of the extracellular flagellar filament to a rigid surface obstructs the rotation of the flagellar motor. Such an increase in the mechanical resistance to rotation (also termed as an increase in the viscous load) causes remodeling of the stator complex, recruiting additional units to the stator to deliver a higher torque to the motor [3]. Such adaptation in structure and function following a viscous load-change is the hallmark of mechanosensitive processes.

The flagellar stator plays a crucial role in mechanosensing. Disrupting the stator function (torque generation) eliminates the viscous load on the motor. The loss of load (and torque) inhibits the ability of individual stator units to bind to the motor [4]. Thus, the flagellar stators are most likely the mechanosensitive components within the flagellum. Consistent with this notion, flagellar stator proteins have been implicated in surface sensing and in the formation of biofilms in a variety of bacterial species [2]. How stator remodeling triggers downstream signaling to initiate biofilm formation upon surface adhesion remains an open question.

### 2.2. Current and future challenges

The biochemical pathways triggered by the stators can be termed as mechanosensitive if the downstream effects are initiated by a change in the viscous load on the flagella. Earlier works suggested that the obstruction of motor rotation in mono-flagellated *Vibrio parahaemolyticus* cells triggered changes in the expression of genes responsible for producing numerous lateral flagella. These lateral flagella are necessary for swarming on surfaces. Changes in gene expression associated with the lateral flagella are triggered by several types of perturbations: growth on solid surfaces, suspension in media with high viscosities, as well as the agglutination of cells with the aid of flagellar antibodies [5]. Although the flagellar viscous loads are likely elevated by each of these perturbations, the magnitudes of load-changes vary drastically between them. In general, there is a lack of information about the correlation between the magnitudes of load-changes and the physiological response—in this example, the expression of lateral flagella. Determining the magnitudes of viscous load-changes needed to trigger biochemical signaling will be important in the future to explain the role of flagellar mechanosensing in signaling.

In contrast, flagellar-mediation of surface sensing in *Caulobacter crescentus* merely requires the presence of functional stator and rotor proteins; the extracellular components of the flagellum are not necessary [6]. In the absence of extracellular flagellar components, no load changes are possible. Hence, mechanosensing is precluded. This suggests that the signal that activates flagellar-mediated biochemical signaling may not always be a viscous load-change [1]. Accurately identifying the signals that activate the flagellar stators at a surface will be crucial to constrain the models of flagellar-mediated surface sensing.

A swimming bacterium experiences a constant viscous drag that is proportional to its speed. The inhibition of flagellar rotation due to surface attachment not only perturbs flagellar activity but it also reduces the drag on the cell body by eliminating motility. Due to the coupling of flagellar functions and motility, it is not straightforward to determine if it is the loss of flagellar functions or the concomitant reduction in the viscous drag on the cell that triggers downstream effects. A case in point is the regulation of K-state transition in *Bacillus subtilis*, which regulates natural competence. The transition probabilities were found to be correlated with the viscous loads on the flagella [7]. However, the alterations in the viscous loads also inhibited motility. It is possible that the reduction in the drag triggered mechanosensors on the cell body to regulate K-state transition probabilities, independent of the flagella. Discriminating between these two mechanisms is a significant challenge.

Bacteria produce different types of chemical entities, including metabolites such as indole and molecules involved in quorum sensing (QS) such as autoinducers [8]. Although swimming bacteria cannot outrun small diffusible chemical stimulants [9], motility does ensure that the local concentrations of the endogenously-produced chemical signals around the cell will be lower relative to the concentrations around immobilized cells. If the chemical signal is very slow to diffuse and the cell is highly sensitive to small differences in the signal levels, downstream signaling could be initiated through the build-up of higher local concentrations of the chemical species. Distinguishing between phenomena triggered by chemical sensing and those due to surface sensing is a future challenge.

### 2.3. Advances in science and technology to meet challenges

The load on the flagellar motor increases significantly only if the cell-filament attachment to the surface meets specific criteria [1]. Hence, visualizing how the flagella interact with the surface is necessary to obtain important insights regarding the magnitudes of load-changes and whether the flagella indeed trigger downstream effects. This is often ignored in studies on surface sensing.

Growth on surfaces may result in multiple activation signals and subsequent downstream effects may or may not arise due to flagellar sensing alone. To address this, cells could be suspended in media of high viscosities to increase the viscous loads on the flagella. The limitation of this approach is the weak dependence of loads on medium viscosities [3]. A better approach, in some cases, is to stall the flagellar motors—which causes the maximum possible load-change—by linking filaments together with anti-flagellin antibodies in the bulk fluid [5]. This technique enables flagellar stalling but only in the case of peritrichous cells. This is because locking the flagella belonging to the same cell eliminates all possible rotational degrees of freedom for the motors. In the case of monotrichous bacteria such as *V. parahaemolyticus*, *C. crescentus*, or *Pseudomonas aeruginosa*, the antibody approach fails to stall the motors as the cell bodies freely rotate along their principal axes (figure 1). Advances in the methods to load the flagella, for example with optical traps [3], in monotrichous bacterial species in the bulk fluid will be critical in delineating the role of the flagella in initiating intracellular signaling.

Genetic modification is a standard approach to determine the role of a particular enzyme in bacterial functions. However, the deletion of a flagellar gene typically inhibits motility, causing several types of stimuli to act on the bacterium at once. In the presence of multiple activating signals, observations can become challenging to interpret. Advances are needed in mechanical stimulation techniques to apply a single type of stimulus. Combining such techniques with dynamic gene perturbation methods [10] is anticipated to reveal bacterial adaptations that may occur following surface adhesion; these are likely to be missed in current approaches that tend to focus on the steady-state responses to the loss of enzymatic function. In particular, measurements of the dynamics of surface adaptation are expected to provide information about the direct as well as indirect interactions in the gene regulatory networks that regulate the transition to the surface-associated states.

### 2.4. Concluding remarks

The bacterial flagellum was historically viewed as an apparatus that enables motility. New research has expanded that view by identifying a role for the flagella in surface sensing and other related phenomena. As discussed, several challenges exist in determining the molecular mechanisms by which flagella trigger the transition from planktonic to surface-associated states. Advances in genetic engineering, microscopy, and mechanical stimulation techniques will be necessary to address some of those challenges.

# Research article

**Authors:** Jing Chen, Beiyan Nan

**Affiliations:** 1 Department of Biological Sciences, Virginia Polytechnic Institute and State University, Blacksburg, VA24061, United States of America; 2 Department of Biology, Texas A & M University, College Station, TX77845, United States of America

## 3. Gliding motility of the social bacterium *Myxococcus xanthus*

### 3.1. Status

Bacterial gliding motility refers to the smooth movements of cells on solid surfaces unaided by flagella or pili. Gliding movements in divergent bacterial groups rely on distinct mechanisms. In the past two decades, gliding motility has become a gold mine for the discovery of novel molecular mechanisms. In fact, the protein complexes driving gliding in *M. xanthus*, *Flavobacterium johnsoniae* and mycoplasmas all represent new types of molecular machineries [11]. The gliding of *M. xanthus*, a rod-shaped biofilm-forming bacterium, is arguably the best studied, because most, if not all, of the components in the gliding complex have already been identified.

About 20 proteins form the core gliding complex of *M. xanthus*, among which a three-component proton channel in the inner membrane functions as the motor (figure 2). Other motor-associated components reside in four different compartments of the cell: the cytoplasm, inner membrane, periplasm and outer membrane [12]. The gliding motors of *M. xanthus* transport partially assembled gliding complexes (dynamic complexes) rapidly along helical tracks. Current evidence suggests that filaments of the bacterial actin homolog MreB provides the platforms for the assembly of motor complexes and might serve as the tracks for their helical motion [13–15]. At the sites where cells contact the substrate surfaces, dynamic complexes assemble with additional motor-associated proteins to form force-generating complexes that span the whole cell envelope [12, 16, 17]. Probably due to resistance from the cell wall, fully assembled gliding complexes reduce their velocity, aggregate and appear nearly static with respect to the substratum (static complexes) [12, 14, 16]. Through either deforming the cell surfaces or directly binding with the substratum, these static complexes exert force between the helical track and the substratum, and drive a corkscrew-like motion of the helical track. As a result, the cell also moves forward like a corkscrew (figure 2) [12]. After transient stalls, static complexes quickly disassemble and resume rapid motion [16].

The motor of *M. xanthus* gliding is remarkably versatile. The motor connects to other cellular machineries and facilitates multiple functions beyond motility. For example, the gliding motor carries a secretion system to deposit polysaccharides on the surfaces of developing spores during *M. xanthus* sporulation [18]. Another unexpected feature of *M. xanthus* gliding is its connection with the synthesis of the peptidoglycan (PG) cell wall through MreB. While moving along MreB filaments, gliding motors also transport MreB [13]. As MreB coordinates the synthesis of the PG cell wall by the rod complex, the major PG synthesis machinery for cell elongation, its transport by the gliding motors affects the distribution and activity of the cell wall synthesis machineries and plays an important role in *de novo* establishment of the cell’s rod shape [13, 19]. Interestingly, rod complexes also drive MreB filaments to rotate circumferentially around the long axis of the cell with nm s^−1^ velocities, which is two orders of magnitude slower than the helical transportation of MreB by the gliding motors [13]. It is still unclear if gliding motility and cell wall synthesis are coupled to each other and how MreB accommodates these two functions with distinct velocities and trajectories.

### 3.2. Current and future challenges

Under regular fluorescence microscopy, most of the gliding-related proteins, including the subunits of the motor, localize diffusively and display rather chaotic movements [13, 14]. Such chaos reflects the sum of fluorescence signals from individual molecules that switch between different behaviors, such as stationary, diffusion and directed motion [13, 16]. Due to this fluid nature, it is technically difficult to dissect the assembly of the gliding complexes. While one could presume that the stationary molecules are assembled into the static complexes, functions of the molecules that undergo diffusion and directed motion remain to be investigated. Most importantly, it is still unclear how the static complexes transmit the proton motive force from the inner membrane to the cell surface. Another challenge for understanding the assembly of the gliding complexes stems from the complexity of the machinery itself. Whereas mutagenesis of the gliding-related genes and pairwise colocalization of the components in the gliding complex have provided important information on the assembly process [20], it is challenging to dissect the dynamic interactions among 20 different proteins.

*M. xanthus* gliding used to be considered as the motility for cells that move as individuals. However, mutants lacking gliding motility are usually not able to form mature biofilms (i.e. fruiting bodies), which is a multicellular process. In addition, the localization and dynamic behaviors of gliding-related proteins are regulated by external mechanical cues, such as substrate stiffness (and potentially the physical contacts with neighboring cells) [16]. Thus, gliding might be part of the mechanism by which cells sense their environment and colony mates. The critical roles of gliding in biofilms remain to be understood.

### 3.3. Advances in science and technology to meet challenges

Single-particle tracking is a technology that allows the collection of rich data on protein dynamics in live cells with unprecedented spatial and temporal resolutions (figure 2). These data reveal features not available from regular fluorescence imaging. For example, single-particle tracking is able to record complex dynamic behaviors, such as different subpopulations of the same protein moving in different modes [13, 16]. The current limit of this technique lies in relatively short trajectories of particles due to the short life time of individual fluorescence tags. Most analyses performed to date have been limited to mean-squared displacement, which is not an ideal parameter for analysing short trajectories. Furthermore, these methods emphasize generic modes of motion, such as Brownian diffusion, anomalous diffusion and directed motion. New methodology development in both experimentation and data analysis is needed to dissect more intricate processes expected in gliding, for instance, the transition of a molecule from one state to another.

Despite the latest advances in microscopy, it remains impossible to simultaneously track a large number of proteins that are typically involved in *M. xanthus* gliding [12]. Thus, experimental data only represent fragmented snapshots of the system, which do not readily lead to coherent mechanistic understanding. Mathematical modeling is a powerful tool for studying the gliding complexes from a systems perspective. Mathematical models can weave fragmented data with basic laws of physics and chemistry, which could suggest mechanistic frameworks and inspire new experiments. A previous mechanochemical model, for example, has successfully brought many critical features of *M. xanthus* gliding under a coherent framework, such as the helical motion of motors, the formation of static force-generating complexes, the rotation of the cell body, the gliding velocity and even the sensitivity of motor clustering to substrate stiffness [14]. Building on new experimental observations, future modeling efforts will play a key role in understanding gliding motility by bridging the gap between complex biological observations and their underlying mechanisms.

### 3.4. Concluding remarks

The machineries of bacterial gliding motility are brand-new additions to the collection of force-generating protein complexes. The behaviors of gliding-related proteins in *M. xanthus* suggest a novel surface-sensing mechanism. Studying such a gliding system offers a rare opportunity to understand a fluid machinery that switches between a chaotic, nonfunctional form and an organized, force-generating form. Understanding the *M. xanthus* gliding complexes, especially the mechanisms of their assembly and force generation, will advance our knowledge far beyond motility itself. Studying gliding will also provide new insights in biofilm formation from the aspect of individual cells. Building upon new experimental techniques and modeling approaches, we expect major breakthroughs in the near future in the research of gliding in *M. xanthus* and many other organisms.

# Research article

**Authors:** Marco J Kühn, Alexandre Persat

**Affiliations:** Institute of Bioengineering and Global Health Institute, School of Life Sciences, École Polytechnique Fédérale de Lausanne, Lausanne, Switzerland

## 4. Dynamics and mechanics of type IV pili

### 4.1. Status

Adhesion and motility are crucial ingredients in the initial steps of biofilm formation. Adhesion allows cells to stay on the surface to grow into biofilms, while motility promotes aggregation and surface encounters. Adhesins play a central role in establishing stable attachment when transitioning from swimming to sessile states. In particular, protein polymers that extend from the cell surface called pili promote rapid adhesion upon surface contact. One class of such filaments called type IV pili (TFP) are essential in initiating biofilm formation in *P. aeruginosa* (*Pa*). In addition to adhesion, single *Pa* cells extend and retract TFP to generate traction and displacements on a surface, thus driving a motility mode known as twitching. *Pa* twitches to explore surfaces and to aggregate into microcolonies that eventually mature into biofilms (figure 3).

TFP are dynamic: they extend over several micrometers and actively retract all within seconds, generating forces up to 100 pN. The motor proteins PilB and PilT function respectively as polymerase and depolymerase at the base of the pilus by shuttling single PilA monomer subunits between the inner membrane and the filament. Successive rounds of extension, attachment and retraction power twitching. This mode of motility is slow compared to flagella-mediated swimming (a few micrometers per minute compared to several micrometers per second for swimming) but allows single cells to move while remaining on a surface.

*Pa* optimizes TFP movement by synchronizing retraction with contact of the pilus tip with the surface (figure 4 top), efficiently converting chemical energy into movement [21]. This suggests that PilT responds to a stimulus generated by tip contact. Single *Pa* cells also sense surface contact with TFP to initiate biofilm formation and promote the production of virulence factors (figure 4 middle). A chemotaxis-like system called Chp reads out a signal generated at the level of TFP, transducing it into a cellular, transcriptional response. Thus, TFP play an active role in regulating biofilm formation not only through motility and adhesion but also at the gene expression level.

About 40 genes are required for twitching motility, categorized as regulatory, architectural and dynamic assembly components, which must act in concert to successively extend and retract TFP. Breakthrough structural studies have pieced together the structure of TFP and their secretion machinery, highlighting important conformational changes during assembly [22, 23]. However, we still lack a clear view of the dynamics of these structures during the process of extension and retraction, and how this feeds back into the process of TFP deployment and biofilm formation.

### 4.2. Current and future challenges

To understand how cells deploy TFP to drive motility, we now must probe how its machinery functions dynamically under mechanical load, in other words out of equilibrium. Specifically, the succession of extension and retraction requires exchange of the molecular motors PilB and PilT at the TFP basal assembly site. How tip attachment stimulates retraction is still unknown, but potentially involves a mechanism where transmission of mechanical or chemical stimuli along the fiber allosterically modulates motor activity (figure 4 bottom) [21]. We anticipate that a similar mechanism regulates the activity of PilU, a secondary motor powering retraction under high force load.

In a similar manner, retraction-induced conformational changes in TFP could activate the Chp surface-sensing system controlling the expression of hundreds of genes including those associated with virulence, biofilm formation and TFP machinery itself (figure 4 bottom). This response is mediated by the interface between TFP and the Chp system, likely involving interaction of PilA and the chemoreceptor PilJ [24].

The Chp system converts the signal generated by TFP activity, which functions on the time scale of seconds, into a cellular response on time scales of hours (figure 4 middle). Activation of Chp increases production of a signaling molecule called cAMP, whose levels depend on the balance between production and degradation. High cAMP levels can persist through multiple generations even after detachment. This memory effect is based on a complex temporal relation between cAMP levels and TFP activity [25]. This long-term adaptation raises the question of how cells integrate discrete signaling events into a continuous cellular response, both on transcriptional as well as protein activity levels. Does each TFP cycle incrementally induce cAMP production? Is the signal integrated within the Chp chemosensory system? How are TFP activity and cAMP levels coupled throughout multiple generations?

### 4.3. Advances in science and technology to meet challenges

Robust visualization techniques are critical to probe the dynamics of TFP. TFP are only ~5 nm thick; imaging such thin extracellular structures is in itself a challenge. Two major advances have brought TFP into focus. First, a label-free imaging technique called interferometric scattering microscopy (iSCAT), which permits single protein visualizations, has been applied to TFP imaging at high spatial and temporal resolutions. iSCAT has been instrumental in identifying the process of surface contact-induced retraction [21]. A second method consists of labeling TFP with a synthetic fluorescent maleimide dye, enabled by substitution of exposed residues for cysteine by mutagenesis. This technique has improved our understanding of TFP-mediated surface sensing in *C. crescentus*, TFP extension/retraction dynamics in *P. aeruginosa* and DNA uptake and biofilm formation in *V. cholerae* [26–28]. iSCAT and fluorescent labeling are complementary techniques that will ultimately allow us to probe TFP dynamics at multiple spatial and temporal scales, from the early stages of TFP attachment all the way to mature biofilms.

The functions of TFP are tightly coupled with their ability to generate and sustain forces. Thus, probing the mechanical properties of TFP and their machinery is necessary to generate a holistic understanding of twitching motility and biofilm formation. Instruments such as optical tweezers and atomic force microscopy (AFM) can probe the mechanical behavior of TFP, for example to measure retraction forces or the elasticity of single filaments [29, 30]. Interestingly, force spectroscopy measurements have highlighted stable conformational changes in single TFP filaments [30]. Whether and how these changes can activate retraction or Chp signaling remain to be tested. Thus, the next technical challenge consists of integrating these mechanical characterization techniques with measurements of cellular outputs. For example, simultaneous measurement of force input with cellular activity by imaging of fluorescent reporters would represent a major advance not only in the field of TFP regulation but in mechanobiology as a whole, providing a direct link between force input and cellular outputs.

In summary, solving the dynamic connection between mechanical input and cellular response such as motor swapping or Chp system activation will require the integration of complementary technologies combining mechanical characterization, direct visualization and measurements of cellular responses [31].

### 4.4. Concluding remarks

To characterize how TFP drive twitching motility and ultimately biofilm formation, we must now probe how their molecular components function dynamically to generate force and displacements. This out-of-equilibrium view will benefit from the current knowledge of genetic parts. This must be accompanied by physical models and novel instrumentation. In particular, simultaneously measuring TFP dynamics, mechanics and how a cell coordinates its internal machinery to appropriately respond at the right time and place is a major challenge. The development of hybrid instrumentation that combines mechanical interrogation with cellular output measurements will help answer these fundamental questions. Linking such biophysical measurements to the evolution and function of TFP in relevant ecological context, as in infections, is a parallel and complementary challenge. Altogether, the study of TFP dynamics embodies an exciting interdisciplinary field that reflects previous studies of motility and mechanosensation in eukaryotes that involve polymer assemblies [32]. This emerging topic in biophysics also has potential in fighting infections by stimulating the development of novel treatments [33].

# Research article

**Authors:** Jean-Louis Bru, Nina Molin Høyland-Kroghsbo, Albert Siryaporn

**Affiliations:** 1 Department of Molecular Biology & Biochemistry, University of California—Irvine, California, CA 92697, United States of America; 2 Department of Plant and Environmental Sciences, University of Copenhagen, DK-1871 Frederiksberg, Denmark; 3 Department of Physics & Astronomy, University of California—Irvine, California, CA 92697, United States of America

## 5. Spatial orchestration of bacterial populations by stress responses

### 5.1. Status

Bacteria rely on physical and chemical cues to adapt to environmental change and to respond to environmental threats. Bacteriophages (phages), which are viruses that infect bacteria, pose a prominent danger toward bacterial populations. Bacteria in turn launch anti-phage defenses, including blocking infection, degrading phage genetic material, and committing altruistic suicide to prevent phage progeny spreading [34]. When the bacterium *P. aeruginosa* is under attack by phages, it triggers a stress response that releases the cell–cell signaling molecule *Pseudomonas* quinolone signal (PQS). This stress signal diffuses away from phage-infected cells. Unlike bacterial swarms that readily collide with uninfected bacteria (figure 5(A)), when bacterial cells sense PQS emitted by phage-infected bacteria, their movement is re-directed away from the signal and away from the area containing infected cells [35] (figure 5(B)). This stress response effectively enables bacteria to distance themselves from virus-infected kin by spatially re-organizing the population. This stress response additionally enables bacteria to survive other threats, most notably antibiotics [35]. The ability of bacterial populations to spatially re-organize through a stress response may have a large impact on bacterial survival in complex environments. However, little is known about how stress responses shape the organization of bacterial populations, particularly in biofilms.

In another example, phage-resistant bacteria can protect fellow phage-sensitive bacteria from phage predation when grown together in a spatially structured colony. Specifically, phage-sensitive cells within the center of a bacterial colony are protected by surrounding phage-resistant bacteria, which provide a barrier. This protection is lost when the bacterial populations are attacked by phage in a liquid culture of planktonic cells, which is spatially homogeneous. This effect highlights that spatial structure can enable phage defense [36].

The spatial aspect of stress responses could have a significant role in bacterial colonization in hosts, where environments are not well mixed. Here, stress signals from bacterial populations could promote resistance or evasion against phage therapies and antibiotic treatments, rendering these strategies to fight bacterial infections ineffective. Understanding the impact of stress responses on the spatial organization of biofilms and microbial communities is thus critical for the development of more effective treatments against pathogenic bacterial species.

### 5.2. Current and future challenges

Several critical challenges need to be addressed to investigate the spatial impact of stress responses on bacterial biofilms. In particular, stress responses impact the production of bacterial metabolites and stress-induced signaling molecules. However, the spatial distribution of these molecules has been difficult to track in bacterial populations. Recent work has demonstrated that biofilms are spatially heterogeneous and have distinct metabolic, transcriptional, and translational activities [37]. For example, bacteria that are located at the periphery of a biofilm are exposed to different stresses, such as phages and antimicrobial compounds, compared to those that are insulated deep within the biofilm core and are starved of oxygen and nutrients [38, 39]. The ability to measure cellular activity associated with metabolism and stress has typically relied on fluorescent reporters and dyes. However, for long-term monitoring of cellular activity, there is a risk of photobleaching reporters, phototoxicity to the cells, and incomplete staining of biofilm with dyes, which do not diffuse well into biofilm cores. Thus, the ability to spatially resolve metabolic activity at the single-cell level as well as signaling molecules and metabolites within a biofilm remains a challenge.

While stress responses are investigated in laboratory settings, the relevance of these studies to natural and host environments can at times be unclear. A critical challenge is the ability to mimic the spatial aspect of these environments under well-controlled conditions in the laboratory. This includes reproducing the physical properties of tissue, mucus layers, immune responses, and gradients in nutrients and oxygen. These challenges make it difficult to study the dynamics of spatially structured multispecies communities in a laboratory setting. In addition, the agonistic and antagonistic interactions between different species greatly affect the overall outcome of bacterial encounters with stresses. Therefore, the establishment of structured biofilm models as multi-species communities of bacteria, phages, and other microbes is central to understanding fundamental interactions across kingdoms and their effect on biofilms.

### 5.3. Advances in science and technology to meet challenges

Recent advances in label-free imaging and tissue culturing technologies have the potential to address many of the current challenges to studying stress responses in microbial communities with minimal impact on cell physiology. Fluorescence lifetime imaging microscopy (FLIM) is a label-free technique that provides a real-time measure of metabolite activity. This method has been applied to bacteria to probe the spatial heterogeneity of the central metabolism activity by tracking the nicotinamide adenine dinucleotide (NAD(P)H) activity in *P. aeruginosa* biofilms at subcellular resolution [40]. Adjustments to the frequency and time domains have enabled FLIM to measure additional metabolites including flavin adenine dinucleotide, and may enable the tracking of additional metabolic species and signaling molecules involved in stress responses. Thus, using optical visualization to detect metabolites and signaling molecules has the potential to decipher the spatial distribution and molecular signatures of structured bacterial communities undergoing stresses. Due to the non-invasive nature of the method, it has the potential to track both metabolic activities spatially and temporally.

Advances in organoid and organ-on-a-chip technologies have the promise to replicate the conditions of the host, including restoring tissue and cellular function, producing mucus layers, and providing representative nutrient environment and gradients more accurately. The technology has been extended to produce many tissues including lung, skin, and gut [41, 42]. Bacterial populations that activate stress responses to phage and antibiotics can be tracked in such devices using the label-free imaging method described here.

### 5.4. Concluding remarks

Stress responses facilitate bacterial survival and resistance to environmental threats from phage infection and antibiotic treatments in part through the rearrangement of the spatial organization of their physical environments. However, significant challenges in imaging and analysis have hampered the ability to investigate the spatial component of stress responses in biofilms. Recent developments in label-free imaging through optical imaging have the potential to address these challenges. Coupling recent advances in imitating host environments through organ-on-chip devices and organoids will enable the study of bacterial stress responses that are relevant in hosts, as well as providing a path to investigating stress responses in multi-species communities in greater detail. Uncovering how bacteria organize structurally to avoid dangers such as phages and antibiotics in natural and host environments may lead to development of new drugs that can inhibit such mechanisms. This, in turn, may render populations of pathogens more vulnerable to treatments with antimicrobials.

# Research article

**Authors:** Jacinta C Conrad

**Affiliations:** William A Brookshire Department of Chemical and Biomolecular Engineering, University of Houston, Houston, Texas, TX 77204, United States of America

## 6. Adhesion of bacteria on solid and liquid interfaces

### 6.1. Status

Cells, teeth, medical implants, ship hulls, oil droplets: bacteria can adhere to nearly any natural or engineered surface. Because adhesion is the first, essential step in the formation of biofilms, resilient surface-associated communities, scientists have long sought to understand where, how, and why bacteria adhere.

Bacterial adhesion is generally lower on surfaces that are hydrophilic, electrically net neutral, smooth, and soft. The microscopic interactions that underpin these macroscopic behaviors are commonly described using thermodynamic models, including the colloidal DLVO theory (which includes electrostatic and van der Waals interactions) as well as an extension that includes acid–base interactions. Deviations between the predictions of these models and experimental measurements, however, indicate that other factors must substantially affect adhesion.

Bacteria bear several types of fibrillar surface structures (curli, pili/fimbriae, flagella) that promote surface adhesion (figure 6) through both specific (i.e. fimbriae–mannose) and non-specific (electrostatic, van der Waals, acid–base) interactions. They can also release proteins, surfactants, and extracellular polymeric substances (EPS), including DNA, that modify the surface properties to favor bacterial adhesion. The use of isogenic knockout mutant strains allows the effects of fibrillar structures and exudates to be systematically investigated. In addition, the surfaces to which bacteria adhere themselves may exhibit pronounced heterogeneity in charge, chemistry, topography, and/or mechanics.

These heterogeneities provide sites on which bacteria can adhere on surfaces that are otherwise unfavorable for attachment—for example, bacteria cling to defects in polymer-brush-coated surfaces [43]. Several studies highlight specific ways in which bacterial surface structures can access surface heterogeneities (for example, type I fimbriae access sub-nanometric roughness [44] or flagella access microscale crevices [45]). Nevertheless, a general understanding of how bacteria interact with heterogeneous surfaces remains elusive.

Bacteria can also adhere at the interface between two fluids (liquid–liquid or liquid–gas). Although adhesion to a hydrocarbon phase dispersed in an aqueous solution is commonly used to semi-quantitatively assess microbial hydrophobicity through measurements of solution absorbance [46] and applied to understand bacterial interactions during biodegradation processes [47], only recently has adhesion begun to be systematically investigated from a physical perspective [48, 49]. Insight into how bacteria use filamentous appendages to adhere to fluid–fluid interfaces will likely inform efforts that employ bacteria to remediate pollutants.

There remains an unmet need for improved understanding of mechanisms controlling adhesion on real, heterogeneous interfaces, both solid and liquid. Development of this understanding requires studies that access variations in adhesion of bacterial populations on spatially heterogeneous surfaces, coupled with measurements of forces during adhesion. This understanding will provide new insight into enhancing bioremediation processes or controlling biofilm formation, either to reduce fouling or promote beneficial biofilm growth.

### 6.2. Current and future challenges

Improved understanding of factors affecting bacterial adhesion requires methods that can access the spatiotemporal heterogeneity of bacteria and interfaces during adhesion.

Imaging methods, including optical (bright-field, fluorescence), AFM, and scanning electron microscopy (SEM) are widely used to enumerate bacteria on a small region of a solid surface. These techniques are limited by the area of the field of view. Although they can be applied in principle to obtain information on adhesion over time, these methods are more often used to image samples at a small number of time points. Both optical and electron microscopy have been applied to characterize bacterial adhesion on liquid–liquid interfaces. Electron microscopy, however, typically requires cryogenic techniques to image at the interface between two liquids. Quartz crystal microbalance with dissipation (QCM-D) is increasingly used to characterize adhesion of bacteria on surfaces. Although it is sensitive to mass changes of order 1 ng occurring over seconds or minutes, QCM-D is not able to resolve adhesion of individual bacteria (whose weight is of order picograms).

In contrast to micron-scale bacteria, fibrillar surface appendages have dimensions less than the optical resolution of light microscopes—flagella are 20–40 nm wide, and type 1 fimbriae are 7 nm wide. Observing these appendages using optical techniques requires that the appendages be chemically modified to bear labels. SEM and AFM can be used to directly image fibrillar appendages. The latter method, when combined with appropriately functionalized tips, can quantify the force applied by an appendage during adhesion. Other force measurement techniques include optical or magnetic tweezers. These methods, which require specialized equipment, provide serial measurements and hence are limited in throughput. Finally, forces can be accessed optically through observation of the deformation of soft surface features such as micro or nanopillars fabricated from a soft polymer.

Methods to characterize bacterial exudates usually involve fluorescence staining via lectins, which attach to specific carbohydrates in the extracellular polymers. Motility experiments employing lectin staining found that bacteria followed ‘slime trails’ as they explored a surface, and that post-division daughter cells were more likely to remain in EPS-rich locations [50]. Staining methods provide useful information on the EPS distribution that affects bacterial adhesion, but lack the temporal resolution needed to characterize surfaces as bacteria continually modify them.

Notably, these imaging methods can be used to characterize heterogeneity on nanometer (AFM, SEM) or micron (optical microscopy) length scales, but have typically not been used in conjunction with spatiotemporally resolved studies of bacterial adhesion.

### 6.3. Advances in science and technology to meet challenges

High-throughput single-cell methods have allowed heterogeneous adhesion to be quantified across large populations over time. These methods, widely applied to motile bacteria, offer great promise to generate new insight into processes controlling bacterial adhesion. Tracking of many individual cells, for example, revealed the adhesion fate of initially transiently-attached *Escherichia coli* bacteria on chemically-modified glass slides [51]. Similarly, single-cell tracking applied to distinct strains of a given species of bacteria revealed vibrational motion with nanoscale amplitudes, which was correlated to the surface expression of fibrillar appendages and/or EPS [52]. Most recently, single-cell tracking of adhesion across a clonal population of *E. coli* revealed phenotypic heterogeneity that could be qualitatively described using a colloidal model with varying numbers of patches [53]. Tracking methods, however, have not been combined with simultaneous dynamic characterization of surface heterogeneities. Specifically, characterization methods to identify and assess changes in surface properties over time, compatible with optical tracking, are needed. Ideally, these methods would allow EPS, surfactants, and proteins to be identified and enumerated along with bacteria, or enable characterization of chemical and/or topographic surface heterogeneity.

New microscopy techniques applied to adhesion can offer qualitatively new information. Very recently, total internal reflectance microscopy (TIRM), coupled with darkfield, revealed that immobile cells are located closer to the surface than mobile cells [54]. Because TIRM offers high spatial resolution in the vertical direction, it may offer an intriguing route to understand the effects of small-scale roughness (whether topographic or chemical in origin) on adhesion processes. Again, methods to simultaneously characterize heterogeneous surface properties and their evolution over time are needed.

Along with new combinations of experimental techniques, temporally resolved experiments will also require new analyses to describe adhesive behavior. These analyses may be empirically guided by machine learning or grounded in models for weak, multivalent attachment adapted from chemistry and biochemistry.

Accurate measurement of forces applied by fibrillar appendages across large populations likely require improvement in throughput for techniques (AFM, tweezers) commonly used for force measurements. Alternatively, traction-based measurements on very soft surfaces may provide a route to characterize the forces applied as adhered appendages retract or move.

Finally, understanding adhesion on liquid–liquid interfaces also demands new experimental techniques. While expression of fibrillar appendages such as fimbriae is known to alter adhesion to oil droplets [55], new methods are needed to characterize the local orientation and (capillary) interactions of these nanometer-scale appendages at liquid–liquid interfaces. Electron microscopy has the necessary resolution but is currently limited to cryogenic measurements. Holographic microscopy may offer an appealing route to resolve the positions of motile bacteria in three-dimensional (3D) near curved interfaces such as oil droplets, as light-scattering methods applied to holograms can yield quantitative information about the 3D position and orientation of bacteria in bulk solution [56].

### 6.4. Concluding remarks

Single-cell analyses have opened up new opportunities for identifying dynamic processes involved in adhesion, through tracking adhesion fate over time and in assessing population-scale variance. Coupling these analyses with necessary advances in experimental characterization of heterogeneous surfaces, along with isogenic knockout mutants, will provide new insight into the mechanisms that operate in a variety of physical settings. Thus, advancing our understanding of bacterial adhesion requires collaboration between microbiologists, physical scientists, and engineers.

# Research article

**Authors:** Francesco Carrara, Yutaka Yawata, Roman Stocker

**Affiliations:** 1 Institute of Environmental Engineering, Department of Civil, Environmental and Geomatic Engineering, ETH Zurich 8093 Zurich, Switzerland; 2 Faculty of Life and Environmental Sciences, University of Tsukuba, Tsukuba, Ibaraki 305–8572, Japan; 3 Microbiology Research Center for Sustainability, University of Tsukuba, 305–0001 Tsukuba, Japan

## 7. Bacterial biofilms on marine particles

### 7.1. Status

Marine particles have recently become a new paradigm in the study of bacterial biofilms. In the ocean, organic particles such as marine snow, formed by the aggregation of fragments of decaying organisms and other detritus, represent nutrient hotspots for heterotrophic bacteria amid a highly dilute background, provide favourable ecological niches for the coupling of different metabolic pathways, and serve as hotbeds of horizontal gene transfer. The microscale interactions between bacteria and particles strongly influence ocean biogeochemistry [57]. By navigating toward, attaching to, and consuming particles, marine bacteria contribute to regulating the transfer efficiency of the biological pump [58], that is, how much particulate carbon sinks from the ocean surface to its depth, where it is buried for thousands of years [59] (figure 7).

Bacteria occur as planktonic cells, many of which actively seek resources through motility and chemotaxis, or in surface-attached, dense communities of great taxonomic and metabolic diversity, called biofilms, where cells are embedded in a self-secreted extracellular polymer matrix and interact cooperatively or competitively with near neighbors [60]. These two modes of growth constitute fundamentally different ecological niches, and are characterized by distinct gene expression patterns and energetic investments. On one side, motile planktonic cells need to assemble, operate, and regulate the flagella through chemosensory machinery. On the other side, surface-associated cells need to produce the extracellular matrix, express and secrete biopolymer-targeted extracellular enzymes, and invest in the secretion of autoinducers involved in QS pathways [61].

Microbial habitats, particularly in marine environments, are often characterized by highly heterogeneous and short-lived nutrient fluctuations, which can drive the ecological differentiation of bacterial populations. Such spatiotemporal heterogeneity at the microscale can afford growth advantages to populations that have flexible strategies, whose phenotypes plastically transition between the molecular programs required for biofilm formation and for planktonic state [62]. Such flexible strategies could be beneficial over specialist strategies that rely on either irreversibly attaching to particle surfaces or chasing particles’ plumes and cells’ exudates while remaining planktonic. The underlying fitness trade-offs accounting for the energetics of these behaviors and physiologies dictate how effective each strategy may be under given environmental conditions [63].

Ultimately, the ability to link this microscale behavior and ecology of bacteria with macro-scale consequences including element cycles and carbon export hinges on a quantitative understanding of bacterial interactions with marine particles, from their rates of encounter, surface sensing and attachment mechanisms, to community formation and assembly, degradation rates and metabolic efficiency.

### 7.2. Current and future challenges

In order to robustly establish the links between microscale community ecology, biogeochemical cycles, and ecosystem functioning, it is necessary to better integrate approaches and technologies from different disciplines and across a wide range of spatial scales (figure 8). Recent studies found regions in the ocean where the element cycles and microbial activity cannot be explained by seawater chemistry alone. Mounting molecular and geochemical evidence indicates that the ecological niches of marine microbes are effectively expanded by particle microenvironments, themselves forged in part by bacterial activity. Scientists have proposed a biogeochemical model for denitrifying and sulfate-reducing microbes [64] that implies that anaerobic metabolism should not be confined to the anoxic waters of coastal regions and tropical oxygen minimum zones, but instead could be found more widely due to the formation of denitrifying microenvironments within particles. These altered microenvironments, which are a consequence of diffusion-limitation and concentrated bacterial respiration, are represented in the model of a spherical particle as concentric shells, in which respiration is fueled sequentially by different chemical compounds [65]. Direct experimental evidence is currently lagging behind, in part because of the intrinsic difficulty in measuring these processes in the field. More efforts are needed to elucidate the biophysical, biochemical, and mechanical mechanisms that could lead to the formation of such onion-layer microenvironments within particles, and to derive the kinetics of the degradation process.

Biogeochemical models typically implement a power-law size-spectrum of particles, which are produced in the surface euphotic zone [58, 59, 64]. The size spectrum then changes with depth due to differential settling, disaggregation and remineralization. However, these models often rely on a set of ad hoc assumptions regarding the bacterial attachment–detachment dynamics and metabolic activity of the biofilms on particles, and on poorly constrained rates of particle settling and disaggregation dynamics. Such unresolved microscale details of microbial particle colonization and biofilm activity, which could deeply affect particle microenvironments and settling dynamics, currently limit the predictive power of large-scale ecosystem models.

### 7.3. Advances in science and technology to meet challenges

In landscape and behavioral ecology, the spatiotemporal structure of a resource landscape is a fundamental driver of individual behavior, species interactions, and community composition and functioning. This body of work was epitomized in general frameworks, such as the theory of island biogeography, the metacommunity concept, or optimal foraging theory. Yet, classical techniques in microbiology and microbial ecology—such as growth in batch cultures and chemostats—largely ignore the spatiotemporal characteristics of microbial habitats. In the context of the biological pump (figure 7), where particles are effectively ‘islands’ for bacteria foraging in the ocean, nutrient heterogeneity represents an essential ingredient, given the strong non-linearity of the bacterial search and uptake processes, to correctly scale up and predict the kinetics of microbial degradation. We foresee stochastic theoretical frameworks that account for this heterogeneity, with the application of first-passage processes that consider distributions rather than the average values, as key to advancing the field of microbial ecology through the inclusion of the spatial and temporal components.

Microbial communities with tightly coupled co-evolutionary histories foraging in a seascape of sedimenting particles—where the behaviors, interactions, and physiological adaptations are observed at the scale of the microbes and of the particles—constitute an ideal system to investigate the fundamental principles of particle–bacteria colonization–dispersal and settling dynamics on one side, and the community assembly, microbial physiology, and metabolic efficiency of biofilms on the other side. Model systems for particle–bacteria interactions in heterogeneous landscapes implemented with microfluidic and millifluidic setups, in combination with chemical analyses, such as NanoSIMS, Raman microspectroscopy, single-cell autofluorescence microspectroscopy, or oxygen microoptodes, and real-time multicolor fluorescence imaging to monitor spatiotemporal dynamics, have great promise to establish an integrative community ecology for microbial biofilms on marine particles.

At the same time, we encourage increasing effort to sample *in situ* microbial activities at the microscale and mesoscale [65, 66]. These endeavors stand to benefit greatly from the application of modern micro-engineering and molecular technologies. Such approaches, in parallel with biogeochemical analyses and remote-sensing techniques, will permit us not only to assess the spatial variation in particle size spectra and chemical composition, but also to simultaneously characterize the functional activities of the associated microbes. As a result, we will be in a position to better estimate the contributions of particle size- and community-dependent microenvironmental processes to water column respiration and biological turnover of particulate organic matter [64].

### 7.4. Concluding remarks

The study of bacterial communities on marine particles will require a combination of field measurements to better constrain their global distribution, laboratory experiments implementing realistic model systems to directly observe spatiotemporal dynamics, and modeling efforts that distill the empirical observations while retaining sufficient physical, chemical and biological complexity. This integrated approach will provide a blueprint for a mechanistic understanding of bacterial communities growing on marine particles, and how these sea-snow microcosms—which constitute the metabolic engines of the ocean—depend on cell aggregate properties, particle and seawater chemistry, and scalars such as temperature, oxygen, or turbulence. We foresee research programs unifying themes from ecosystems and microbial spatial ecology with systems biology and evolutionary theory as the most promising to achieve predictive frameworks that help assess the cycling of the elements in the ocean and its adaptation under future global climatic perturbations.

# Research article

**Authors:** Yves V Brun, Gregory B Whitfield

**Affiliations:** University of Montreal, Faculty of Medicine, Montreal, Quebec, H3C 3J7, Canada

## 8. Surface sensing by type IV pili

### 8.1. Status

The capacity for bacteria to perceive their environment and respond through phenotypic adaptation is crucial for fitness. Extracellular appendages have been implicated in surface sensing due to their role in mediating surface contact during biofilm formation. TFP are appendages composed of helical filaments that are extended from the cell surface via polymerization of the inner-membrane localized pilin subunit PilA [67]. The tip of the pilus can adhere to various substrates, then undergo retraction through PilA depolymerization and reincorporation into the membrane [21]. This mechanism can allow TFP to pull bacteria toward a nearby surface. Thus, TFP represent one of the first points of contact between cell and surface, suggesting that the initial signaling events that upregulate biofilm formation may occur via the pilus. In support of this, our work in *C. crescentus* demonstrated that pilus-mediated surface contact leads to rapid deployment of holdfast adhesin and the transition toward irreversible surface attachment [26] and stimulation of cell differentiation [68].

How then does TFP contact with a surface signal intracellularly? One emerging hypothesis from studies of *P. aeruginosa* points to the combined sensing of pilus tension during retraction and detection of depolymerized PilA that has been reincorporated into the membrane after retraction (figure 9; [24]). The latter function has been linked to the sensor PilJ, which directly interacts with monomeric PilA, leading to activation of the Chp chemosensory system and increased production of the bacterial second messenger 3′,5′-cyclic adenosine monophosphate (cAMP; [24]). Chp activation increases TFP extension and retraction activity, while cAMP signals for increased expression of the extracellular pilus biogenesis protein PilY1 [69]. PilY1 contains a putative mechanosensing von Willebrand factor type A domain, which is thought to sense pilus tension [70], and couples this sensing to stimulation of the diguanylate cyclase (DGC) SadC via the membrane-spanning pilus alignment subcomplex composed of PilMNOP [69]. Activated SadC produces cyclic-3′,5′-dimeric guanosine monophosphate (c-di-GMP), the major bacterial signaling molecule regulating the switch to biofilm formation. Thus, in *P. aeruginosa* it is thought that a hierarchical feed-forward mechanism, the Pil–Chp system, amplifies small changes in pilus dynamics to effect TFP-mediated surface sensing and promote biofilm formation after surface contact. In *C. crescentus*, detection of depolymerized PilA by the sensor kinase PleC and subsequent stimulation of pilus retraction was recently demonstrated [71], suggesting that this mechanism of signaling may not be unique to *P. aeruginosa*.

### 8.2. Current and future challenges

While the intracellular signaling events that follow TFP-mediated surface contact have been explored, the mechanistic details of how the surface is initially sensed by the pilus and how the signal is conveyed across the cell envelope are unknown. Part of the challenge lies in the inability to directly visualize pili dynamically as they extend and retract, as available techniques require fixation of cells or disruption of pilus activity. As a result, the role of pilus dynamics in surface sensing has been indirectly inferred from pilus motor mutants [24]. Since export of PilY1 to the cell surface in *P. aeruginosa* requires functional TFP [69], this approach may convolute the interpretation of data gathered. Further confusion stems from the observation that PilY1 performs a separate surface-sensing role in regulating *P. aeruginosa* virulence that is independent of TFP [70]. *P. aeruginosa* also has a second distinct surface sensing pathway, the Wsp system, that is capable of stimulating c-di-GMP synthesis independently of the Pil–Chp pathway, but that is dependent on functional TFP for maximal activation [72]. Thus, without a method to correlate intracellular signaling phenotypes with pilus dynamics, these intertwined pathways in *P. aeruginosa* have proven difficult to fully disentangle.

The scope of our understanding of TFP-mediated surface sensing in bacteria is very narrow since it is derived almost exclusively from *P. aeruginosa*. In other model piliated organisms, such as *V. cholerae*, *C. crescentus*, or pathogenic *Neisseria*, almost nothing is known regarding how TFP surface-sense. This is further complicated by the existence of three distinct mechanisms for TFP assembly, type IVa (T4a), type IVb (T4b), and type IVc (T4c or Tad), which each utilize a subset of unique protein components for pilus biogenesis [67]. Many bacterial species also utilize multiple non-redundant TFP systems. *V. cholerae*, for example, have mannose-sensitive hemag-glutinin (MSHA) T4a pili, toxin co-regulated T4b pili, and chitin-regulated T4a pili [73], while *P. aeruginosa* has an understudied T4c pilus alongside the established T4a system [67]. These species-specific differences in pilus utilization, combined with multiple TFP biogenesis mechanisms, mean that progress in comprehension of surface-sensing mechanisms in one bacterial species may not translate universally.

### 8.3. Advances in science and technology to meet challenges

Two recent complementary advances in microscopic visualization of TFP have allowed for the direct observation of pilus dynamics in live cells. The first involves replacement of a native residue in PilA with a cysteine, allowing for labeling of the pilus fiber with thiol-reactive maleimide dyes (figure 10; [26, 74]). Critically, cysteine-mutagenized PilA does not disrupt pilus function or dynamics, nor does labeling of pili with maleimide dyes. Furthermore, this technique was used to label pili produced via the T4a, T4b, and T4c assembly mechanisms [26]. Since this approach utilizes fluorophores, other bacterial components can be differentially labeled and simultaneously visualized, such as the *C. crescentus* holdfast whose deployment we demonstrated was correlated with a cessation in pilus dynamics [26]. In a separate study, we combined pilus labeling with fluorescent visualization of DNA to demonstrate the mechanism of TFP-mediated natural transformation in *V. cholerae* [73]. Therefore, this approach can be used to correlate pilus dynamics with other cellular phenotypes, including those relevant to surface-sensing. It is also highly accessible, requiring only an epifluorescence microscope to visualize labeled pili [74].

Pilus labeling has proven beneficial for dissecting pilus dynamic activity; however, this approach requires that the bacterial species under study be genetically tractable, and that the major pilin subunit be tolerant of cysteine mutations. To address these issues, a technique that has previously been successful in the label-free visualization of actin filaments *in vitro*, iSCAT, was recently adapted for live bacterial imaging [21]. This approach has allowed for label-free observation of pilus dynamic activity, thus bypassing the need for genetic manipulation of the bacteria in question.

While both fluorescent pilus labeling and iSCAT allow for the observation of pilus dynamic activity in live cells, pilus labeling has the additional advantage that any maleimide-conjugated molecule can hypothetically be linked to the pilus [74]. We employed this rationale to covalently attach bulky PEG5000-maleimide to extended pili in *C. crescentus*, which blocked their retraction due to physical obstruction. This led to the stimulation of holdfast production in the absence of surface contact, providing the first direct evidence that resistance encountered during pilus retraction is a cue for surface contact [26]. Since this approach does not require the cell to contact a surface, contributions to surface-sensing phenotypes by parallel mechanisms, such as the Wsp system in *P. aeruginosa* [72], can be avoided. Furthermore, separate pilus systems, such as those from *V. cholerae*, could be independently examined to determine their relative contributions to surface-sensing phenotypes.

### 8.4. Concluding remarks

Emerging surface-sensing data from a variety of bacterial species implicate TFP as a critical piece of the puzzle. Our data suggests that, at least in the T4c pilus system of *C. crescentus*, tension on retracting pili stimulates surface sensing [26]. Further exploration of other TFP assembly mechanisms from other bacterial species using pilus labeling and retraction blocking methodologies [74] will demonstrate whether this concept is generalizable. Of critical importance in the long term is the identification of the specific mechanosensing component of the TFP architecture that perceives pilus tension upon retraction, and whether this component is conserved between T4a, T4b, and T4c systems. Given that *C. crescentus* does not have a homolog of *P. aeruginosa* PilY1, it is possible that different TFP assembly mechanisms utilize different mechanosensors, or alternatively that PilY1 is not the pilus tension sensor in *P. aeruginosa*.

# Research article

**Authors:** Calvin K Lee, Jaime de Anda, William C Schmidt, Ramin Golestanian, George A O’Toole, Gerard C L Wong

**Affiliations:** 1 Department of Bioengineering, University of California—Los Angeles, Los Angeles, California, CA 90095, United States of America; 2 Department of Chemistry and Biochemistry, University of California—Los Angeles, Los Angeles, California, CA 90095, United States of America; 3 California NanoSystems Institute, University of California—Los Angeles, Los Angeles, California, CA 90095, United States of America; 4 Max Planck Institute for Dynamics and Self-Organization (MPIDS), D-37077 Göttingen, Germany; 5 Rudolf Peierls Centre for Theoretical Physics, University of Oxford, Oxford OX1 3PU, United Kingdom; 6 Department of Microbiology and Immunology, Geisel School of Medicine at Dartmouth, Hanover, NH 03755, United States of America

## 9. Multigenerational signaling and memory during early biofilm formation

### 9.1. Status

A pivotal step in the formation of bacteria biofilms on surfaces is surface sensing, which is the process through which cells detect a surface and begin modifying their behavior to form a biofilm. During surface sensing, bacteria utilize different appendages or extracellular structures, such as flagella and TFP. Besides surface sensing, these appendages are also involved in a variety of behaviors, such as motility, interaction with their environments or neighboring cells, and responding to chemical gradients. These appendages and their associated motor machinery then activate cellular responses that are primarily controlled by intracellular secondary messenger molecules, such as cyclic diguanylate (c-di-GMP) and cyclic AMP (cAMP). Recent studies have elucidated different aspects of surface sensing for a variety of bacterial species. In *V. parahaemolyticus*, restriction of flagellar rotation by either surface binding or a high viscosity environment triggers a surface response [5]. Flagellum rotation is a mechanical signal that triggers signal transduction pathways in *B. subtilis* [75] and in *P. aeruginosa* [76]. In *C. crescentus*, the retraction of surface-bound TFP is a signal for surface sensing [26]. In *P. aeruginosa*, retraction/extension of TFP activates a hierarchical cascade of cAMP-mediated and then c-di-GMP-mediated cellular signaling pathways [24, 69]. In *V. cholerae*, cells swim near a surface using their flagellum while using their TFP to touch the surface, with the strength of this TFP–surface interaction determining the type of swimming motility and subsequent attachment and biofilm formation [77].

Until recently, it was not known why cells that contact the surface do not always surface sense. During the initial stages of biofilm development known as ‘reversible attachment,’ bacteria that land on the surface tend to also detach from the surface. However, eventually a subset of cells will commit to growing on the surface in a process known as ‘irreversible attachment.’ Clearly, surface sensing seems to be heterogeneous, and the process itself seems to change with time. Given that the molecular pathways for surface sensing have been elucidated [24, 69], the next step is to uncover the general concepts needed to understand how these pathways function in a community of cells engaging the surface.

### 9.2. Current and future challenges

Until recently, it was not known why cells that contact the surface do not always surface sense. During the initial stages of biofilm development known as ‘reversible attachment,’ bacteria that land on the surface tend to also detach from the surface. However, eventually a subset of cells will commit to growing on the surface in a process known as ‘irreversible attachment.’ Clearly, surface sensing seems to be heterogeneous, and the process itself seems to change with time. Given that the molecular pathways for surface sensing have been elucidated [24, 69], the next step is to uncover the general concepts needed to understand how these pathways function in a community of cells engaging the surface.

To address these problems, quantitative methods are needed to simultaneously visualize and analyze appendage activity and molecule concentrations in live cells at fast sampling rates (milliseconds to seconds) for data collection periods that are relevant to biofilm time scales (from hours to days). While segmentation of bacteria is relatively straightforward, being able to reconstruct the lineage histories of all cells in the form of family trees is a non-trivial problem. Lineage analysis, combined with single-cell tracking, is crucial for parsing spatiotemporal interactions between cells in a community. Finally, a unified theoretical framework is necessary for representing and analyzing the complexity of these events.

### 9.3. Advances in science and technology to meet challenges

In our recent work, we investigated the complex temporal relationship between cAMP and TFP in *P. aeruginosa* PA14 communities during the early stages of biofilm formation by combining lineage tracking with cAMP measurements (via fluorescence reporters) and TFP activity (via motility tracking) [25], all at single-cell resolution. We found that bacteria displayed adaptive adhesion behavior that changes with time, where planktonic ‘surface-naïve’ cells would overwhelmingly attach poorly to a surface, but ‘surface-sentient’ cells previously exposed to a surface could attach strongly and rapidly proliferate on the surface. This adaptive adhesion resulted from cells that have achieved correlated cAMP levels and TFP activity in the form of damped, coupled oscillations. Furthermore, we developed a Turing stochastic model based on the key components of the Pil–Chp chemotaxis-like surface-sensing system to understand the temporal aspect of these correlated cAMP–TFP oscillations. This model demonstrates that the time lag between observing high levels of cAMP and the corresponding high TFP activity can occur several division generations apart, which indicates that signals and responses can propagate across multiple generations. This cross-generational signaling amounts to a kind of communication between ancestors and descendants. Finally, we show that these cAMP–TFP oscillations can result in the emergence of non-planktonic behavior on the surface, in the form of irreversibly attached lineages that have suppressed levels of TFP activity and detachment, and thereby facilitate exponential increases in surface cell populations observed in biofilm formation. A cognate phenomenon was also observed for c-di-GMP and surface translational motility in *P. aeruginosa* PAO1 [72]. Here, multigenerational c-di-GMP signaling resulted in a heterogeneous population, where some cells produced more EPS and started microcolonies and other cells explored the surface with high surface motility.

These results suggest a social dimension to the strategies employed by bacteria while sensing and colonizing a surface. In the case of *P. aeruginosa* PAO1, surface-sensing cells that deposit EPS on the surface can facilitate the surface commitment and guide microcolony formation of spatial neighbors [78]. A complementary strategy that we have discovered in *P. aeruginosa* PA14 (a strain in a discrete lineage of *P. aeruginosa*) involves multigenerational signal propagation via intracellular secondary messengers, which is passed down via division and helps temporal neighbors (i.e., progeny) ‘remember’ prior surface contact to facilitate attachment. Both strategies are viable and can be advantageous under different circumstances.

### 9.4. Concluding remarks

Figure 11 illustrates how multigenerational lineages contribute to the surface population increase typically observed during early biofilm formation. Due to multigenerational signaling by second messengers, bacteria will appear to behave as if it were not simply a matter of ‘every bacterium for itself’. Not only does cAMP allow planktonic cells to remember a surface, it implies that the pivotal transition relevant to the onset of irreversible attachment is defined in terms of cooperative behavior between temporal neighbors (involving multiple generations of ancestors and descendants) rather than in terms of single-cell events, so that descendants have higher probabilities of staying on the surface.

# Research article

**Authors:** Kyle A Floyd, Fitnat H Yildiz

**Affiliations:** Department of Microbiology and Environmental Toxicology, University of California—Santa Cruz, Santa Cruz, California, CA 95060, United States of America

## 10. Deciphering the c-di-GMP-mediated motile to sessile transition in *V. cholerae*

### 10.1. Status

*V. cholerae*, a natural bacterial inhabitant of aquatic environments, is a facultative human pathogen and etiologic agent of the acute diarrheal disease cholera. Essential to the environmental lifecycle of *V. cholera* is the formation of matrix-encapsulated multicellular biofilm communities (figure 12); however, host ingestion of biofilm particles is linked to bacterial hyperinfectivity and exacerbated disease [80].

Biofilm formation in *V. cholerae*, and many other pathogenic bacterial species (e.g. *E. coli*, *P. aeruginosa*, *Salmonella* sp., etc) is controlled by the secondary messenger signaling molecule 3′,5′-cyclic diguanylate monophosphate (c-di-GMP, figure 12). Production and degradation of c-di-GMP is mediated by DGC and phosphodiesterase (PDE) enzymes, respectively, and is modulated by environmental signals sensed by the enzymes [81]. Upon production, c-di-GMP interacts with receptors (e.g. proteins, mRNA riboswitches) to facilitate/regulate cellular processes. *V. cholerae* has a total of 62 predicted DGC/PDE genes, along with a diverse repertoire of known c-di-GMP receptors [81]. In *V. cholerae*, elevated c-di-GMP levels promote the transition from a free-swimming planktonic state to a biofilm lifestyle, through reduction of flagellar motility, stimulation of surface attachment, and regulation of extracellular matrix component production (i.e. proteins, *Vibrio* polysaccharide) [80].

Biofilm formation of *V. cholerae* requires flagellar motility and production of the type IVa MSHA pilus (figure 12) [82, 83]. Our work has revealed that the MSHA polymerization ATPase, MshE, is a high-affinity c-di-GMP receptor [82]. Recently, we showed that c-di-GMP levels control MSHA extension/retraction dynamics via regulation of MshE functional state [84]. Dysregulation of MSHA pilus dynamics resulted in altered near-surface motility, and attenuated long-term surface colonization requisite for biofilm formation [84]. Our work has provided further evidence of a direct inverse relationship between cell surface MSHA pilus production and flagellar motility. However, the identity of surface sensing pathway(s), and any potential cross-regulatory pathway interactions by which c-di-GMP controls cessation of flagellar motility and production of MSHA pili, remains to be elucidated. Understanding these pathway(s), which facilitate the motile to sessile transition, is vital to deciphering the mechanism(s) that initiate *V. cholerae* biofilm formation, and to the development of anti-biofilm strategies to reduce biofilm-associated hyperinfectivity.

### 10.2. Current and future challenges

Essential to understanding the role of c-di-GMP in the motile to sessile transition is the ability to rapidly determine alterations in c-di-GMP levels. To this end, we adapted a c-di-GMP-responsive fluorescent biosensor, where interactions of c-di-GMP with a double tandem mRNA riboswitch (Bc3-4—*Bacillus thuringiensis*) promote expression of tRFP in a dose-dependent manner (figure 13(A)) [85, 86]. Development of the c-di-GMP-responsive biosensor has afforded the ability to determine c-di-GMP levels at single-cell resolution, which, combined with near-surface cell tracking, has allowed direct analysis of temporal c-di-GMP production dynamics upon surface attachment [84, 85]. These analyses led to identification of key DGCs with probable roles in surface-sensing pathways [85].

Recent advances in pilus visualization, utilizing an amino acid substitution in MshA and thiol-reactive fluorescent dyes, has given us the ability to directly visualize cell–surface MSHA levels and dynamics in real time (figure 13(B)) [84]. With these methods, we established MSHA as dynamic retractable type IVa pili, determined the impacts of intracellular c-di-GMP levels on pilus production/dynamics, and better defined the role of MSHA in regulation of surface colonization [84].

While these methods have aided in the advancement of our understanding of the role of c-di-GMP in *V. cholerae* surface sensing [84, 85], they are not without their limitations or challenges. The biosensor we utilized relies on c-di-GMP interactions with mRNA riboswitches to facilitate expression of tRFP, making it a translation-based reporter. This limits the readout of the biosensor to a reflection of the global intracellular c-di-GMP pool [84, 85], as well as limiting the temporal resolution given the dependency on tRFP translation and protein maturation. Alterations in the global c-di-GMP pool are significant; however, full dissection of surface-sensing pathways will require detection of alterations in localized c-di-GMP levels across the cell.

Real-time visualization of MSHA pili is dependent upon disulfide-linking of fluorescent dyes. Restraints of the labeling methodology, along with photostability of the dyes, limits temporal analysis of pilus production and dynamics of pili activity. Analysis of MSHA-mediated surface-sensing pathways, and examination or links between MSHA- and flagella-mediated pathways, will require rapid real-time pilus visualization techniques.

Finally, current approaches for deciphering *V. cholerae* surface-sensing pathways involve comparisons of single or in tandem DGC/PDE gene deletions, compared to a wild-type control. These strains may exhibit defects in surface attachment, which precludes us from analyzing their role in surface-mediated signaling pathways that occur post-attachment.

### 10.3. Advances in science and technology to meet challenges

Deciphering c-di-GMP-dependent MSHA- and flagella-mediated surface-sensing pathways will require further advances to our current methodologies. Circumventing the limitations of current c-di-GMP-responsive biosensors, for single cell visualization of localized temporal c-di-GMP concentrations, will require development of more rapid diagnostic systems. A FRET-based fluorescent c-di-GMP biosensor has been described, which utilizes a genetically-encoded c-di-GMP-binding protein (YcgR) from *Salmonella enterica* serovar Typhimurium [87]. Such biosensors improve both the spatial and temporal c-di-GMP analysis, and their adaptation for use in *V. cholerae* would allow us to monitor alterations in localized c-di-GMP pools across the cell in response to various stimuli (i.e. surface attachment).

Overcoming limitations in real-time MSHA pilus visualization will require the development of label-free methodologies. Development of such techniques is in process, as seen through the recent the application of iSCAT to the analysis of TFP dynamics in *P. aeruginosa* [21]. Further advancement in technologies such as iSCAT will allow for more in-depth analysis of MSHA, as well as flagella, and surface attachment dynamics in real time.

Sidestepping analysis of single and in tandem gene deletions will require advancement of gene knockdown technologies in bacteria. Recent strides have been made to introduce CRISPR-based gene interference (CRISPRi) into *V. cholerae*, using an inducible enzymatically-dead version of the Cas9 protein, to prevent translation of specific mRNA transcripts targeted by a single guide RNA [88]. CRISPRi targeting of c-di-GMP signaling genes identified as having probable roles in MSHA- or flagella-mediated surface sensing pathways, immediately following surface attachment, would aid in deciphering their role in surface-mediated signaling pathways.

In concert application of these methodologies (e.g. FRET-based c-di-GMP biosensor combined with CRISPRi) stands to greatly enhance our ability to visualize and determine the underlying mechanisms that facilitate c-di-GMP-dependent flagella- and MSHA-mediated surface sensing that control the motile to sessile transition.

### 10.4. Concluding remarks

While we have made great strides in understanding the role of c-di-GMP in reducing flagellar motility and facilitating MSHA-dependent surface attachment, further study is required to define the underlying c-di-GMP signaling pathways behind these processes. The ongoing and future technology advances outlined here will aid in this process, as well as determining possible flagella-MSHA regulatory cross-talk. Defining *V. cholerae* c-di-GMP-dependent surface-sensing pathways that lead to biofilm formation could support the development of therapeutics to attenuate biofilm-associated hyperinfectivity and pathogenesis.

# Research article

**Authors:** Shuai Yang, Fan Jin

**Affiliations:** CAS Key Laboratory of Quantitative Engineering Biology, Shenzhen Institute of Synthetic Biology, Shenzhen Institutes of Advanced Technology, Chinese Academy of Sciences, Shenzhen 518055, People’s Republic of China

## 11. Optogenetic control of bacterial c-di-GMP production

### 11.1. Status

The bacterial cellular c-di-GMP is produced by DGCs and is broken down by specific PDEs [89]. Traditional manipulation of c-di-GMP was implemented by introducing a constitutive or inducible promoter to overexpress the genes encoding its synthesis or breaking enzymes. However, such systems are not adequate for manipulating intracellular c-di-GMP levels in real time due to the inevitable time delays of the activation of gene expression. Additionally, because of the slow degradation of proteins and hard removal of chemical inducers, the reverse process is slow and difficult. Furthermore, the interrogation of signaling c-di-GMP events with high spatial precision, i.e., to stimulate the defined cell types or localized cell populations, using diffusible chemicals is virtually impossible. By contrast, optogenetic approaches are excellent tools for the rapid, noninvasive and targetable manipulation of cells with unprecedented spatiotemporal resolution and thereby devoid of the foregoing deficiencies. A promising approach is to couple the synthesis or degradation of intracellular c-di-GMP to light signals, i.e., optogenetic manipulation of bacterial c-di-GMP production.

The difficulty of engineering novel photoreceptors associated with light-activated DGC or PDE activity is the first impediment to the optical control of c-di-GMP production. In 2014, Gomelsky’s group first reported a semi-synthetic receptor with light-dependent DGC activity, designated BphS [90], to synthesize c-di-GMP upon the irradiation of near-infrared light, and light enhanced the intracellular c-di-GMP levels by 50-fold in *E. coli*. While there have been several reports of light activation PDEs for light-degradation of c-di-GMP, such as BlrP1 [91] SL2 [92] and SseB [93], the low photodynamic range or the need to introduce extra genes limits the applications in living bacterial cells. Until 2017, a blue light-activated PDE with a good performance, named EB1 [94], was described and optimized to complement BphS. Thus far, the combined employment of BphS and EB1 allows for the optogenetic control of bacterial intracellular c-di-GMP production reversibly using two distinctive lights.

c-di-GMP is a ubiquitous second-massager molecule in all major bacterial phyla and the downstream of its signaling regulation pathways are closely linked to various aspects of bacterial physiology and behavior, such as motility and surface phenotypes. Because of this, it is not surprising that the bacterial behavior relevant to c-di-GMP signal transduction can be controlled by light through the optogenetic manipulation of intracellular c-di-GMP. As a simple example, in the *E. coli* cells that expressed BphS, red light-induced increase in c-di-GMP levels resulted in impaired swimming pattern and Congo red-pigmented colonies [90]. At a faster time scale, transient changes of c-di-GMP concentrations using optogenetic approaches could modulate bacterial chemotaxis behavior [95]. Of course, there has been an enormous number of applications in regulating gene expression that we will not discuss here. However, the optogenetic manipulation of c-di-GMP has broad potential applications, and could be expected for various purposes, such as anti-biofilm, bioengineering and even diagnosis (figures 14 and 15).

### 11.2. Current and future challenges

Light-regulated c-di-GMP systems are attractive because of the versatile output of bacterial behavior of c-di-GMP signaling pathways. The straightforward phenotypic changes under static conditions have been researched, and we thus need new approaches and ideal to explore some of the most exciting applications of the c-di-GMP optogenetic modules. From this point, that synthetic biology-inspired engineering approaches and the development of new tools combine to apply the c-di-GMP-based optogenetic modules to solve real-world problems or complement the existing technologies is the ongoing challenge.

Because the acute-to-chronic virulence lifestyle transitions of bacterial cells are involved in c-di-GMP signaling pathways and the light that activates BphS can penetrate most deeply into tissue, an application that will probably develop strongly in coming years is the use of optogenetic control of c-di-GMP for host interactions in mammalian models with the aim to develop innovative therapeutics. Therefore, another exciting challenge is the rational design of genetic circuits based on BphS to engineer bacterial cells to perform a specific therapy function.

### 11.3. Advances in science and technology to meet challenges

Recent developments by several groups have shown that optogenetic manipulation of c-di-GMP production can be used in various fields for distinctive purposes. Some specific advances and strategies for addressing the aforementioned challenges are as follows.

#### 11.3.1. Biofilm dispersal

In the formation of biofilms, cells with low c-di-GMP levels have increased motility and repressed secretion of biofilm-associated exopolysaccharides, and they are prone to be freely planktonic individuals. In this regard, using light to induce biofilm dispersal is a fascinating strategy to fight biofilms. That is, cells could be engineered with PDE photoreceptors, and light stimulates the decrease of c-di-GMP levels and further induces the cells to escape from the clusters of biofilms. A recent study by Pu *et al* has shown that optogenetic manipulation of c-di-GMP levels in the engineered bacterial cells is capable of the prevention of biofilm formation [96]. The authors constructed the cells by incorporating the light transcriptional activation of a natural c-di-GMP effector protein rather than the direct light activation of semi-synthetic PDEs, hence leading to the system with long response time on the scale of hours. As far as we know, however, it is the first attempt to fight biofilms via optogenetic control of bacterial c-di-GMP production. Even though we still need plenty of advances in the design of more sophisticated genetic circuits to apply the optogenetic modules to fight the clinical infections caused by bacterial biofilms, the research provides a prototype of dispersing biofilms by utilizing engineered c-di-GMP-based optogenetic systems and we expect to develop novel antimicrobial strategies using other synthetic optogenetic modules.

#### 11.3.2. Bioprinting

One of the ways in which c-di-GMP directs the biofilm formation in all major bacterial phyla is the controlling secretion of exopolysaccharides, including alginate, PEL and PSL. These components crosslink to form the scaffold for the 3D architecture of biofilms, and thereby have ‘glue’ properties similar to bioinks in bioprinting. Together with the spatial resolution of optogenetics, it is reasonable to apply the c-di-GMP optogenetic systems for the printing of living bacteria cells. For example, engineered c-di-GMP-based optogenetic modules, composed of BphS and BlrP1, have been successfully applied to construct patterned bacterial biofilms of *P. aeruginosa* with a spatial resolution of 10 *μ*m [97]. Remarkably, this strategy could be expanded to print living mammalian (or other species) bacterial cells to create functional ‘living materials’ and complement the available bioprinting techniques. However, success in the bioprinting area of optogenetics still requires new development of illumination methods to control the 3D structures of the printed cells and new techniques for the enhancement of spatial precision.

#### 11.3.3. Bacterial cell-based therapies

The acting downstreams of c-di-GMP signaling pathways include large amounts of transcription factors that can respond to the change of intracellular c-di-GMP levels, subsequently providing a wide variety of promoters to control the production of drugs for different treatment options. Additionally, as previously mentioned, the near-infrared absorption spectrum of BphS is advantageous for the penetration of mammalian tissue. These two aspects make BphS attractive for the development of innovative therapeutics. Shao *et al* recently showed a BphS-based genetic circuit to drive light-activated production of insulin or its analogues as antidiabetics, and bacterial cells engineered with this optogenetic circuit were implanted to maintain glucose homeostasis in diabetic mice [98]. In such cell-based therapies, the on/off state of light simply corresponds to the firing/ceasefire of drugs, leading to much finer temporal precision than in chemical molecular-controlled release systems. Future research in this area could be focused on developing digitized and personalized systems for different types of diseases, and translating the optogenetically engineered cell-based therapies into clinic.

### 11.4. Concluding remarks

Superior spatiotemporal resolution of optogenetic approaches and engineered photo sensory proteins, i.e., PDE and DGC photoreceptors, allow the regulation of many aspects of bacterial physiology and behavior for various applications through the optogenetic control of c-di-GMP or the engineering of its signal transduction pathways. In bacterial cells, when making rational adaptations of genetic circuits, c-di-GMP-based optogenetic modules can be served for photocontrol of intrinsic bacterial behavior, such as motility, attachment and biofilm formation, and the corresponding phenotype transitions can be exploited for the application of anti-biofilms and bioengineering such as bioprinting. Moreover, because of the masses of downstream effectors, light-activated c-di-GMP modules can be employed for optogenetic control of gene expression to perform a specific function, such as production of a therapeutic protein. In this respect, combined with the penetrating property of near-infrared light, engineered bacterial cells with a rational c-di-GMP optogenetic circuit can be linked to diagnosis and therapy, which eventually will lead to the development of innovative therapeutics.

# Research article

**Authors:** Masanori Toyofuku, Leo Eberl, Nobuhiko Nomura

**Affiliations:** 1 Microbiology Research Center for Sustainability, University of Tsukuba, Tsukuba, Ibaraki 305–8572, Japan; 2 Faculty of Life and Environmental Sciences, University of Tsukuba, Tsukuba, Ibaraki 305–8572, Japan; 3 Department of Plant and Microbial Biology, University of Zürich, 8008 Zürich, Switzerland

## 12. Membrane vesicles and quantized bacterial signaling

Masanori Toyofuku^1,2^, Leo Eberl^3^, Nobuhiko Nomura^1,2^

### 12.1. Status

The term quorum sensing (QS) describes the phenomenon where bacteria communicate with each other through the release and perception of signaling molecules in order to coordinate group behaviors [99]. This is accomplished by a regulatory system that controls gene expression in a population density-dependent manner through monitoring the concentration of the bacterial signal. As many of the regulated genes encode public goods, QS is considered a social trait. QS systems are ubiquitously distributed across bacterial taxa and have been identified as a key regulator of bacterial virulence. Interference with QS has proven to be an effective strategy for the development of antivirulence drugs, some of which have been commercialized [100, 101]. The general consensus is that bacteria use QS to launch the expression of costly virulence factors, such as toxins, tissue-degrading enzymes, biofilm-forming polysaccharides and biosurfactants only at high cell densities, when the cooperative sharing of these molecules at the group level is most efficient.

Hence, knowledge of the molecular mechanisms underlying QS and the identification of QS-controlled phenotypic traits greatly contributes to our understanding of bacterial lifestyles and behaviors.

### 12.2. Current and future challenges

Although the QS paradigm assumes free diffusibility of the signal, evidence has accumulated that many of the communication molecules are hydrophobic and thus have a poor solubility in water. These hydrophobic signals are often associated with the cell envelope but are not released from the cell. Although in some bacteria transport systems have been identified, it is mostly unknown how hydrophobic signals are released from and taken up by bacteria.

Recent studies have shown that hydrophobic signals can be released and dispersed by membrane vesicles (MVs) (figure 16), which are formed by bacteria via different mechanisms. However, knowledge about the role of MVs in bacterial communication is scarce.

#### 12.2.1. Signaling via MVs

*P. aeruginosa* produces the quinolone signal PQS (2-heptyl-3-hydroxy-4-quinolone), which has an octanol–water partition coefficient (log *P*) of 3.60. PQS has been demonstrated to mediate its own packaging and transport by stimulating outer membrane formation through intercalation into the outer membrane [102]. Another hydrophobic signal (log *P* = 3.05) that is delivered by MVs is Ea-C_8_-CAI-1 [(*Z*)-3-aminoundec-2-en-4-one], which is produced by *Vibrio* species [103]. A recent study showed that hydrophobic members of the most widespread class of bacterial signals in Gram-negative bacteria, the *N*-acyl homoserine lactones (AHLs), are also trafficked by MVs. AHLs consist of a homoserine lactone ring and an acyl side chain of 4–20 carbons. The hydrophobicity of the signal increases with the length of the acyl side chain. *Paracoccus denitrificans* produces the highly hydrophobic (log *P* = 6.05) signal *N*-hexadecanoyl-L-homoserine lactone (C16-HSL) that accumulates in the outer membrane of the bacterium [104]. C16-HSL has been shown to be released by MVs and packaged within MVs so it can also disperse in aquatic environments [102]. Importantly, the amount of signaling molecules carried by an MV is much higher than what is needed to trigger the QS response in a bacterial cell that fuses with the MV. Such fusion events are stochastic and will result in a binary population where cells would either be quorate or not. This signaling mechanism is fundamentally different from the classic QS model, which assumes the free diffusion of the signal in the environment until a critical concentration is reached that induces the QS response synchronously in the majority of cells. Heterogenous gene expression within an isogenic population may serve several functions, such as division of labor and the formation of phenotypically distinct subpopulations. However, the full breadth of the socioecological impact of binary signaling systems is yet to be examined. The chemical nature of the signal molecule and its concentration define the maximum distance (the ‘calling distance’) over which two cells can communicate with each other in diffusion-based signaling models. By contrast, signals ‘quantized’ by MVs rather resemble messages in a bottle, which can travel very long distances. Moreover, because the signals within MVs are protected from degradation, their life span may be increased and thus MVs may also transport messages from the past.

Trafficking signaling molecules by MVs has several advantages over simple diffusion. While in the case of diffusion the signals are shared by all members of the bacterial consortium, MV-mediated signaling allows for targeted communication between cells in polymicrobial communities by specifically fusing with certain bacteria. Furthermore, MVs can sequester signals from the environment, and this signal piracy will allow bacteria to trigger their QS response in the presence of cooperating bacteria that produce the signal [105]. Although our knowledge of how bacteria communicate with each other in polymicrobial communities is limited, MVs are very attractive vehicles to traffic the signals. At present, it is unclear how MVs specifically target certain bacteria. Understanding the molecular mechanism behind this specificity would allow us to shed light on the communication networks in natural communities. This information could also be used to develop strategies for the manipulation of natural consortia. In addition to signal molecules, MVs also carry DNA, and it will be a highly interesting future line of research to investigate how specific trafficking between bacteria affects the spread of antibiotic resistances and DNA transfer in general.

Taken together, knowledge of the spatiotemporal production and distribution of MVs is critical for understanding the dynamics of QS systems that rely on hydrophobic signals. MVs have been shown to be highly abundant in the matrices of biofilms [106, 107], and their production appears to be linked to the release of extracellular DNA through explosive cell lysis, a recently discovered mechanism of MV formation [108]. The current interest in MV research is also triggered by the fact that a better understanding of the mechanisms of MV formation will open novel avenues for the production of tailored MVs for medical and biotechnological applications. MVs show great promise as an innovative platform for the development of vaccines and for applications in nanotechnology.

#### 12.2.2. Different types of MVs

MVs have been demonstrated to serve many biological functions that reflect the diversity of components they can carry as cargo. While previous studies have identified many biological functions of MVs, this was under the assumption that bacteria produce only one type of vesicles, namely classic outer MVs. However, recent work has shown that Gram-negative bacteria produce different types of vesicles that differ in their structures and contents as a consequence of their different biogenesis routes [109]. It is therefore likely that some of the well described biological functions of MVs, including DNA transfer, bacterial killing and the release of cytoplasmic proteins, are in fact intrinsically linked to certain MV types. Additional work will be required to determine how different MV formation routes determine the structures, compositions and functions of different MV types.

### 12.3. Advances in science and technology to meet challenges

Recent advances in microscopy have enabled us to visualize the production of MV particles in real time. Super-resolution microscopes that allow imaging beyond the diffraction limit with fast scan-speed and sensitive detectors have become available, and were used to visualize how MVs are emerging from the bacterial cell surface. Live cell imaging has been particularly valuable for understanding the MV biogenesis process [108], as a snap shot cannot resolve the process in time. Future work to unravel the specificity of cargo delivery to specific cell types will also strongly depend on real-time imaging techniques. This would provide direct visual evidence of how bacteria communicate with each other via MVs and how DNA is exchanged between cells. Deploying microfluidic chambers to grow cells on surfaces could resolve some limitations currently used in microscopic setups. In combination with fluorescent QS reporter strains, these approaches will allow us to explore the impact of MVs on QS at single-cell and single-MV particle level.

Although flow cytometry has been used for analyzing MVs in some studies, sorting of bacterial MVs has remained a very challenging task due to the small size of the particles. To overcome this problem, different MV types could be specifically captured by beads prior to sorting, similar to methods used in exosome research. For this approach it will be essential to identify markers that are specific for the different MV types. An attractive emerging technology to directly sort MVs is based on lab-on-chip devices that sort small particles based on different methods. Although most of these techniques are currently developed for the analysis of particles with the size of bacterial cells, future developments may allow a scaling down to the level of MVs.

### 12.4. Concluding remarks

MV research is a rapidly expanding field that has also brought new concepts to bacterial communication. MVs appear to be highly diverse [108], and thus one of the most promising ways to determine the impact of MVs on bacteria and eukaryotic host cells will be to track the fate of single MV particles in real time.

# Research article

**Authors:** Lori A Zacharoff, Mohamed Y El-Naggar

**Affiliations:** 1 Department of Physics and Astronomy, University of Southern California, Los Angeles, California, CA 90089, United States of America; 2 Department of Chemistry, University of Southern California, Los Angeles, California, CA 90089, United States of America; 3 Department of Biological Sciences, University of Southern California, Los Angeles, California, CA 90089, United States of America

## 13. The electrical frontier of biofilms

### 13.1. Status

Electron transfer drives the bioenergetics of bacterial respiration by coupling the oxidation of electron donors to the reduction of electron acceptors. In an interesting twist relevant to our understanding of surface-attached biofilms, bacteria are not necessarily limited to *soluble* electron donors (e.g. organic molecules) or acceptors (e.g. O_2_) that enter the cell, but can extend their electron transport chains to external *solid* surfaces. Metal-reducing and oxidizing bacteria are the prototypical model organisms for this EET process [110]. EET is also exploited in bioelectrochemical technologies where electrode-colonizing biofilms catalyze electricity generation, waste degradation, bioremediation, electrosynthesis of fuels, and enable new concepts for microbial electronics [111].

The last decade has witnessed remarkable advances in identifying microbe–surface EET mechanisms [110], including via small molecule shuttling, cell surface multiheme cytochrome (MHC) conduits, and micrometer-scale bacterial nanowires, which include cytochrome-containing outer membrane extensions and cytochrome polymers [110, 112] (figure 17). Recent studies even demonstrate that the biofilm itself can serve as a conductive matrix, allowing distant cells electrical access to the redox-active surface [111]. EET may also facilitate syntrophic metabolic relationships where one organism performs electron transfer to another [113].

Much of our mechanistic understanding of biofilm EET is derived from studies of uniform single-species biofilms. However, we know that multispecies biofilms contain complex 3D structures with a high degree of spatial and chemical heterogeneity that gives rise to emergent properties [114]. This complexity is yet to be fully explored in electroactive biofilms where the redox-active surfaces, beyond providing structural support, function as energy sources/sinks that power the biofilm. Given the recent advances in understanding EET mechanisms, and the finding that EET is widespread across phylogenetically diverse bacteria beyond the prototypical environmental isolates [118], we argue that now is the perfect time for convergence between the EET and biofilm research communities.

### 13.2. Current and future challenges

While EET is heavily characterized in metal-respiring bacteria (especially the Gram-negatives *Shewanella* and *Geobacter*), we now know that other phylogenetically diverse bacteria and archaea conduct EET [110]. This realization has taken new impetus after a pioneering study by Light *et al* [115], who identified a new EET mechanism in the Gram-positive fermentative pathogen *Listeria monocytogenes*. While the biological purpose of this observed microbial EET in the host is unknown, the genes underlying this mechanism are present in other Firmicutes, including pathogens and members of the human microbiota known to form biofilms (e.g. *Clostridium* spp., *Enterococcus* spp., *Streptococcus* spp.). To overcome the challenging thick cell wall, these organisms appear to rely on a cell–surface associated flavoprotein in concert with soluble flavins that mediate electron transfer to external acceptors. The reliance on flavins is a curious feature for organisms not equipped with flavin synthesis machinery, and highlights the extent to which EET might rely on the use of resources supplied by the mammalian host or by other bacteria, raising the possibility of emergent EET properties resulting from interspecies interactions in biofilms. The discovery of this mechanism, which does not rely on well-studied cytochrome conduits of metal-respiring bacteria, highlights one of the central challenges: *since genome gazing is not predictive of this functionality, how can we discover new forms of EET?*

Electrochemistry (amperometry, voltammetry, etc), when coupled with genetic methods, has revealed mechanistic insight into EET by single-species biofilms [111]. A challenge again arises, when one considers the need to characterize the prevalence and mechanisms of EET in the more heterogeneous multi-species biofilms. Yates *et al* applied interdigitated microelectrode arrays (IDA) to characterize electron transport through a complex seawater community capable of coupling EET from a cathode to CO_2_ reduction (electroautotrophy) [116] (figure 18). In contrast to single-species biofilms, this community exhibited a more complex electrochemical behavior with seemingly distinct signatures for electrode–cell and cell–cell EET. These findings highlight the need for new electrode technologies that can interrogate electron flow with the spatiotemporal resolution needed to match the heterogeneity (e.g. 3D nature and variable spatial arrangement of the syntrophic partners) in complex biofilms.

### 13.3. Advances in science and technology to meet challenges

While EET is widespread across the microbial world, the molecular mechanisms appear to be as diverse as the organisms, making it difficult to predict which bacteria possess this ability from sequencing and culture-independent molecular approaches. This is a major bottleneck for discovery and highlights the need for new technologies for high-throughput screening of microbial electrochemical activity. The traditional microbial electrochemical reactors used in single-species and enriched biofilm EET studies are continuously improving, but are bulky and time-consuming, making them unsuitable for parallel activity measurements from multiple cultures. We are aware of nascent efforts for harnessing advanced CMOS processing for massively parallel direct measurements of EET, as well as rapid photometric screens that detect interactions between EET-capable microbes and electrochromic nanomaterials [117]. We envision that our knowledge base of bacterial EET will benefit immensely from advances in ‘hyphenated’ techniques that combine electrochemistry, imaging, and spectroscopy.

As illustrated by our discussion of complex multi-species biofilms, there is also a clear need for advanced nanoscale electrode concepts for recording electrochemical signals in three dimensions with sufficiently fine spatiotemporal resolution. Here it is instructive to take inspiration from recent developments in neurotechnologies, such as injectable mesh electronics that can map electrical activity in living brain tissues at single-neuron resolution [118]. It is exciting to consider the application of such technologies to measure electrochemical activity within thick complex multi-species biofilms, perhaps allowing us to map the 3D flow of electrons and resolve intra-biofilm niches.

Finally, we note that electrons are not the only charges flowing in biofilms. Over the last few years, Süel and co-workers have honed our understanding of ionic (potassium) signaling within and across bacterial communities [119]. This ion-channel-mediated signaling allows for the coordination of metabolic activity both within and across *B. subtilis* biofilm communities, and the extracellular potassium emitted from biofilms can even influence the behavior of distant cells of different species [119]. These ionic communication strategies are yet to be investigated in the context of biofilm EET, where the electron and ion flows may interact in a non-trivial manner, giving rise to yet-undiscovered emergent properties.

### 13.4. Concluding remarks

To date, most of our fundamental knowledge of biofilms comes from studies on non-respired surfaces. The question of how EET and ionic signaling through complex 3D biofilm architectures (and across the biotic–abiotic interface) might impact our understanding of biofilm development represents fertile ground for discovery. Exploring this *electrical frontier of biofilms* will require major science and technology advances, such as high throughput electrochemical screens that assess the prevalence of EET/ionic signaling across phylogenetically diverse communities, and advanced nanoscale electrode concepts (beyond surface patterns) that interface to the real 3D architecture of biofilms. The implications are immense, ranging from an improved fundamental understanding of biofilm energetics and communication to novel strategies for either suppressing harmful biofilms or boosting the activity of biofilms in bioelectrochemical technologies and microbial electronics.

# Research article

**Authors:** Sibel Ebru Yalcin, Nikhil S Malvankar

**Affiliations:** 1 Department of Molecular Biophysics and Biochemistry, Yale University, New Haven, Connecticut, CT 06516, United States of America; 2 Microbial Sciences Institute, Yale University, New Haven, Connecticut, CT 06516, United States of America

## 14. Seeing is believing: novel imaging methods help identify structure and function of *Geobacter* nanowires in electricity-producing biofilms

### 14.1. Status

One of the most attractive attributes of *Geobacter sulfurreducens* is its ability to generate high current density by forming biofilms on the anodes of microbial fuel cells with high electronic conductivity rivalling those of synthetic polymers [120]. This conductivity enables bacteria to transport electrons, derived from metabolism, over hundreds of cell lengths [120]. Intrinsic conductivity measurements of living biofilms, using a contact-free four-electrode method, have demonstrated conductivity over centimeter distances, 10,000 times the size of a cell (figures 19(a) and (b)). These conductive biofilms also provide a unique opportunity to develop living electronic materials that can self-repair and replicate [120]. Filamentous appendages on the surface of *G. sulfurreducens*, known as microbial nanowires, confer conductivity to biofilms [120]. However, the nanowires’ identity and underlying conduction mechanism have remained unclear.

We have applied cryo-electron microscopy (cryo-EM) and multimodal AFM, to determine composition and structure of nanowires to correlate with their conductivity [112, 122]. These nanowires were previously thought to be type 4 pili (TFP) composed of PilA protein [120]. However, a cryo-EM structure of conductive filaments isolated from electricity-producing biofilms revealed that, rather than TFP, these filaments are polymerized cytochrome OmcS and OmcZ, with hemes seamlessly stacked over micrometers, providing a continuous path for electron flow [112, 122] (figures 19(c)–(e)). Discovering the nanowire structure represents a technological advance: the proteins forming the nanowires, not known *a priori*, were identified from the cryo-EM density and multimodal AFM imaging [112, 122]. These discoveries also provide a conceptual advance because cytochromes were not known to form filaments naturally, allowing bacteria in biofilms to extend electron transport over hundreds of cell lengths [112, 122].

### 14.2. Current and future challenges

#### Physiological need for two nanowires:

These studies thus solve a longstanding mystery of how microbial nanowires move electrons. Our findings also help explain how both OmcS [123] and OmcZ [124] are critical for electricity production by biofilms. The deletion of the *omcS* gene inhibits electricity production during the early stages of biofilm growth [120, 123], whereas *omcZ* is essential for high current density [124]. In wild-type biofilms, OmcZ accumulates near the electrode, whereas OmcS is distributed throughout the biofilm [125].

However, the role of OmcS in biofilm conductivity was overlooked because Δ*omcS* biofilms were conductive and produced high current densities in microbial fuel cells when grown over prolonged growth conditions [120]. Therefore, we evaluated the possibility of proteins other than OmcS capable of forming nanowires in biofilms [112, 122]. Using an AFM-based multimodal imaging platform, we have found that growing *G. sulfurreducens* biofilms, under current-producing conditions using an electric field, stimulates production of previously unknown OmcZ nanowires that exhibit 1000-fold higher conductivity than OmcS nanowires [122]. The electric field is maximum near the biofilm–electrode interface and decreases away from the electrode. Therefore, OmcZ expression will be maximum at the interface. This could explain the maximum accumulation of OmcZ [125] and highest metabolic activity [126] observed near the biofilm–electrode interface. We therefore propose that *G. sulfurreducens* use OmcS nanowires during the early stages of thin biofilm growth and produce OmcZ nanowires at later stages of growth to form 100 *μ*m-thick biofilms by utilizing the 1000-fold higher conductivity of OmcZ nanowires than OmcS nanowires [127].

#### Role of pili:

Previous studies proposed that conductive *G. sulfurreducens* filaments were TFP because the *pilA* deletion mutant strain lacked conductive filaments [120] and biofilms could not generate high current density [124]. However, there has never been any direct evidence that conductive *Geobacter* filaments are composed of PilA [112]. Instead, the filament composition was inferred from indirect evidence, including the presence of PilA monomer in biofilms and filament preparations [120]. However, PilA is involved in additional functions such as the translocation of OmcS and OmcZ to the outer surface ([112, 127] and references therein). Overexpression of PilA is accompanied by overproduction of OmcS, OmcZ and extracellular filaments that result in the formation of highly conductive biofilms [120, 125, 127]. We did not find any filaments with structure consistent with TFP either in filaments from current-producing wild-type biofilms or in previously published images of intact, cell-attached filaments [112, 122, 127]. Our analysis of previously published filament images showed structure similar to OmcS nanowires [112, 127]. Furthermore, conductivity measurements along the length of individual OmcS and OmcZ nanowires showed values similar to previously published conductivity values for filaments of wild-type [112, 127] and W51W57 strain [122, 127] respectively. All these results suggest that these previous studies interpreted OmcS and OmcZ nanowires as pili. It is therefore important to identify the conditions under which *G. sulfurreducens* can naturally show pili, and determine their composition, structure and conductivity. These studies will help evaluate whether TFP serve as nanowires or provide hitherto unknown functions.

### 14.3. Advances in science and technology to meet challenges

#### Correlate nanowire structure with function using cryo-EM and AFM:

We have correlated cryo-EM studies with high-resolution AFM to confirm that the same OmcS and OmcZ nanowires were studied for both conductivity measurements and structure determination [112, 122] (figures 20(a)–(g)). For example, AFM revealed an axial height periodicity with a 20 nm pitch (figure 20(f)), consistent with the helical pitch determined by cryo-EM (figure 20(a)) [112, 127]. This distinct axial periodicity and the substantial thickness difference observed for OmcS and OmcZ nanowires versus other filaments (figures 20(b)–(f)) were used to confirm that the same nanowires were studied for both structural and conductivity studies. Such correlative studies will ensure that many types of filaments can be readily distinguished, and identical filaments can be studied via multiple methods by mapping their structural features and linking them with functional properties.

#### Contact-free, intrinsic measurements of protein conductivity:

We have applied a four-electrode technique (figure 19(b)) for contact-free measurements of intrinsic electron conductivity in individual protein crystals [128]. We find that the voltage and temperature dependence of protein conductivity are severely impacted by contact resistance with the commonly-used two-electrode method. Our methodology will help to set standards for reporting protein conductivity for accurate comparison of different protein systems.

#### Development of multimodal imaging and spectroscopic methods:

It is important to go beyond the correlative methods to overcome issues associated with sample transfer and storage as well as biological variability [129]. Therefore, we are using multimodal imaging methods to interrogate multiple biomolecular properties within a region of interest on a single platform [122, 127]. Such simultaneous analysis of chemical and functional properties will generate multidimensional datasets as a function of a large number of parameters such as time, pH, temperature, voltage, light, and other external stimuli [129]. These data sets are necessary to apply machine learning, decision theory and artificial intelligence-based approaches to develop autonomous discovery of novel biomolecules involved in electron transport through biofilms. Therefore, we are developing *in situ*, label-free capabilities that will simultaneously image chemical, mechanical and electronic properties of biomolecules using complementary nanoscale tools with a broad spectral range and high spatial and temporal resolution. Previously we have used electrostatic force microscopy (EFM) (figures 20(h)–(k)) to visualize electron transport (bright region in figures 20(k) and (l)) under ambient conditions that have demonstrated conductivity in individual *Geobacter* nanowires [131] in a manner similar to carbon nanotubes [132] (figure 20(l)). We are now combining such functional studies with chemical imaging-based molecular mapping using infrared nanospectroscopy [122, 127, 133]. We have visualized water molecules as they bind to surfaces [133]. By mapping amide vibrational modes of proteins, we have also visualized the secondary structure and pH-induced conformational changes in OmcS and OmcZ nanowires to correlate with conductivity [122]. These studies have revealed that lowering the pH induces formation of beta sheets in these nanowires. This conformational change improves the stacking of hemes and enhances the conductivity and stiffness of nanowires. Such quantitative imaging of structural, electrical, mechanical and optical properties will provide comprehensive information about how to improve performance of microbial nanowires and biofilms. The next step will be to develop *in situ* functional imaging of samples on a transmission electron microscopy grid using a liquid cell design. This approach of combining AFM-based functional imaging with cryo-EM and cryo-tomography of whole cells will help to elucidate the structure–function relations of living cells and associated biomolecules in their native environments.

### 14.4. Concluding remarks

In summary, by applying cryo-EM and AFM together, we have demonstrated the feasibility of correlating structure with function for OmcS and OmcZ nanowires isolated from *G. sulfurreducens* biofilms. Such studies will help identify design principles to use microbial nanowires for the development of new types of materials and sensors to interface living cells with electronics. The next challenge is to correlate structure with function *in situ*. The AFM-based approaches are ideally suited for such multimodal imaging studies because they are inherently label-free and can be used under various physiological environments to induce conformational changes. These imaging methods will help develop microbial nanowire-based multifunctional biofilms with tunable electronic, optical and mechanical properties.

# Research article

**Authors:** Mauricio D Rojas-Andrade, Allon I Hochbaum

**Affiliations:** 1 Department of Materials Science and Engineering, University of California—Irvine, Irvine, California CA 92697, United States of America; 2 Department of Molecular Biology & Biochemistry, University of California—Irvine, California, CA 92697, United States of America; 3 Department of Chemistry, University of California—Irvine, Irvine, California, CA 92697, United States of America; 4 Department of Chemical and Biomolecular Engineering, University of California—Irvine, Irvine, California, CA 92697, United States of America

## 15. Mapping metabolic heterogeneity in bacterial communities

### 15.1. Status

Bacterial cells growing in biofilm communities exhibit marked phenotypic differences compared to free-swimming planktonic cells. The driving forces of this differentiation are complex and dependent on the heterogeneous chemical environments cells experience within these biofilm structures. These cell niches can be dynamic in nature, generating a physiological heterogeneity within the individuals of these communities that fluctuates over position and time. These conditions give rise to a wealth of biochemical mechanisms through which new phenotypes emerge to maximize community fitness and adapt to environmental challenges [134].

Of these mechanisms, change in metabolic state stands out as one of the most conserved and effective means by which bacteria overcome adverse conditions. Under nutrient limitation bacteria experience a stringent response, suppressing metabolic turnover and imparting protection against oxidative stress and antibiotic exposure [135]. Indeed, metabolic state is the main determinant of antibiotic efficacy [136]. Within bacterial cultures, an antibiotic tolerant sub-population of individuals, called persisters, survive due to dormant metabolism rather than genetically adapted resistance mechanisms [137]. This population presents unique challenges to the treatment of infections with antibiotics as they are intrinsic in every bacterial community and increase the likelihood of antibiotic resistance [138]. In order to understand the development of persisters within populations and their relationship to antibiotic resistance more broadly, techniques to assess these metabolic underpinnings with single-cell resolution are necessary.

Metabolic variability is also important in biotechnology applications. For example, EET, by which microorganisms ‘eat’ or ‘breathe’ via extracellular electron donors or acceptors, respectively, is a critical metabolic step in microbial electrochemical devices and bioprocessing technologies [139]. Spatially resolved metabolic mapping can provide insights into design improvements of these technologies.

Our current understanding of the complex factors contributing to metabolic heterogeneity, and its resulting phenotypes, is limited by the destructive, low-resolution nature of conventional analytical, biochemical, and genetic methods discussed below, which lack the dynamic detail characteristic of these systems. The development of techniques that provide adequate spatial and temporal resolution of cellular metabolism *in vivo* therefore promise scientific insights into mechanisms pertaining to antibiotic resistance, persister formation, and electron transfer.

### 15.2. Current and future challenges

The variation of metabolic function within bacterial communities strongly depends on the complexity of regulatory and biochemical networks in constituent cells and the resulting cooperative behavior. For example, one might intuit that cells on the exterior of a biofilm, having greater access to nutrients and oxygen, would exhibit higher metabolic activity than those in the interior. A direct contradiction of this assumption was observed under nitrogen limitation in *B. subtilis* biofilms in which oscillatory metabolic changes were observed in peripheral cells as a result of a nutrient co-dependence with interior cells [140]. These oscillations have mean periods less than cell division times, highlighting the role of regulatory and biochemical networks that control metabolism at time scales of seconds to hours [141]. Consequently, tools to resolve the temporal and spatial dynamics of metabolism are critical to understanding the complex function emergent bacterial communities (figures 21 and 22).

Conventional metabolomic technologies of mass spectrometry and nuclear magnetic resonance excel at accurate molecular analysis of homogenized metabolite solutions. Achieving spatial resolution of data at the single-cell level (~1 *μ*m^3^) desired for metabolic mapping of bacterial communities, however, remains an open challenge. These methods must be improved upon or novel techniques developed in order to deliver sufficient spatiotemporal resolution. Imaging mass spectrometry provides a route to spatial mapping, albeit destructively, of metabolic utilization of nutrients. Nanospray secondary ion mass spectrometry (nano-SIMS) is one such platform which, measuring multiple isotopic labels (^13^C, ^15^N, ^31^P, ^32^S) simultaneously, not only provides remarkable spatial resolution (~100 nm), but also gives insights into the detailed metabolic pathways active throughout the community based on incorporation of isotope-labeled nutrient sources [139].

Early attempts at mapping metabolic activity relied on fluorescence imaging, characterizing its spatial dependence by localization of redox-sensitive dyes or nucleic acid stains (see reference [134] for a detailed review of methods). As a comprehensive example, *in situ* fluorescence identification of cells (via DNA hybridization) and assessment of metabolic rates (via RNA quantification) provides a rich, species-resolved map of metabolic activity [142]. Coupled with confocal microscopy, these methods can be quantitative or semiquantitative, and have excellent spatial resolution, but sample processing and dye toxicity limits their ability to assess metabolism within bacterial communities in real time. A less invasive readout of metabolic activity involves the use of reporter strains, which tie expression of fluorescent proteins to the expression of genes strongly correlated with metabolic activity. The advantages of genetic approaches toward characterizing metabolic heterogeneity are clear, but destructive imaging requirements and the need for genetically addressable strains limits their application *in situ* and *in vivo*.

### 15.3. Advances in science and technology to meet challenges

Promising methods for mapping metabolic heterogeneity in bacterial communities must provide high spatial and temporal resolution. Advances in magnetic resonance imaging (MRI) resolution and specialized sample chambers for biofilm growth have brought the analytical specificity of NMR to bear on this problem. With the ability to resolve metabolitespecific ^1^H chemical shifts, 3D MRI is capable of mapping metabolite concentrations over large areas [143]. However, long imaging times (~h), low spatial resolution (60 *×* 60 *×* 20 *μ*m^3^ voxels), poor sensitivity (~mM), and specialized sample holders limit the potential of this technique to provide the desired resolution of metabolic information. With ever-improving scan rates and excellent spatiotemporal resolution, on the other hand, confocal microscopy-based spectroscopic techniques stand out as ideal candidates for ideal metabolic mapping methods.

Label-free spectroscopic methods do not require genetic manipulation of the organisms under study and, coupled with confocal microscopy, can provide metabolic information within complex communities with single-cell resolution on the time scale of seconds to minutes. Confocal Raman spectroscopy (CRS), though not completely label-free, is one such method that uses long wavelength laser excitation for non-destructive imaging of bacterial samples. The metabolic activity can be measured by the intensity of the C–D stretching band resulting from non-destructive incorporation of deuterons into organic compounds in cells exposed to D_2_O, such as through the citric acid cycle or proton exchange on acidic moieties. This approach has also been utilized to study the effects of phenazine production on the antibiotic susceptibility of cells in *P. aeruginosa* biofilms [144]. Exploiting the significant signal enhancement of stimulated Raman scattering spectra, the heterogeneous distribution of metabolically active cells can be mapped in biofilms with high spatial resolution and in close to real time. The metabolic information provided by CRS is limited to this narrow metric of C–D vibration intensity, prohibiting detailed analysis of metabolic pathway utilization, for example in comparison to nanoSIMS, and it potentially alters metabolic activity through the kinetic isotope effect.

FLIM is another technique that can be integrated into a confocal microscope and used to map metabolic activity. FLIM can differentiate between the excited-state lifetimes of protein-bound and freely soluble endogenous fluorophores—such as NADH—involved in central metabolic function. Several studies have shown that the FLIM signal is indeed correlated with metabolic activity in bacteria, most recently demonstrating the connection with bacterial growth phase and exposure to bacteriostatic and bactericidal antibiotics, and the confocal capability provides subcellular fluorescence resolution [145]. Confocal FLIM is a true label-free technique to assess metabolic characterization with single-cell resolution, though studies have yet to determine whether FLIM (or CRS) signatures can be correlated to persister phenotypes. If successful, these spectroscopic imaging methods would represent ideal candidates for mapping metabolic heterogeneity and the emergence of persisters in bacterial biofilms.

### 15.4. Concluding remarks

Understanding the intrinsic and environmental factors that create metabolic heterogeneity within bacterial communities is challenging, and requires precise measurement of physiological changes at the single-cell level and at representative time scales. Conventional metabolomic methods provided only a glimpse into these systems, as adequate spatial and temporal resolution have not been achieved simultaneously without also being destructive. The benchmarking of non-destructive methods will be essential for characterizing the dynamic metabolic heterogeneity in bacterial communities. Spectroscopic methods such as CRS and FLIM have demonstrated the potential to meet these criteria and will undoubtedly be utilized for more in-depth studies in the near future. The knowledge gained from these studies will provide answers to fundamental questions pertaining to bacterial physiology both at the individual and community level, with significant implications for medicine and biotechnology.

# Research article

**Authors:** Jing Yan, Howard A Stone, Ned S Wingreen, Bonnie L Bassler

**Affiliations:** 1 Department of Molecular, Cellular and Developmental Biology, Yale University, New Haven, Connecticut, CT 06511, United States of America; 2 Department of Mechanical and Aerospace Engineering, Princeton University, Princeton, New Jersey, NJ 08544, United States of America; 3 Department of Molecular Biology, Princeton University, Princeton, New Jersey, NJ 08544, United States of America; 4 Lewis-Sigler Institute for Integrative Genomics, Princeton University, Princeton, New Jersey, NJ 08544, United States of America; 5 The Howard Hughes Medical Institute, Chevy Chase, Maryland MD 20815, United States of America

## 16. Single-cell resolution imaging of bacterial biofilms

### 16.1. Status

Biofilms are ubiquitous surface-attached communities of bacteria embedded in an extracellular matrix [146]. Biofilms represent a predominant bacterial lifestyle in nature and in man-made environments—ranging from the ordinary, e.g., sewage systems, to the exotic, e.g., Yellowstone hot springs. However, biofilms can be problematic in clinical and industrial settings: they can cause chronic infections, such as in cystic fibrosis patients, and they can damage materials in industry.

Imaging is increasingly playing a central role in biofilm analyses. Indeed, high-resolution imaging of biofilm internal structures has revolutionized our understanding of how cells are organized in biofilms, how extracellular matrix components are distributed, and how biofilm structures respond to environmental challenges including shear flow, starvation, and osmotic stress.

Fixation of biofilm samples treated with DNA stains enabled bulk visualization of biofilm cells and the general contours of the biofilms. Recently, techniques including fluorescence *in situ* hybridization (FISH) of 16S rRNA were combined with fixation to define biogeographies inside polymicrobial biofilms. A seminal example (figure 23 and reference [147]) shows an oral biofilm in which combinatorial labeling and spectral imaging FISH (CLASI-FISH) was combined with metagenomic sequence analysis to reveal the spatial organization of the different bacterial genera. Specifically, it was shown that the oral bacterial consortium consisted of a radially arranged, nine-taxa structure organized around a core of filamentous corynebacteria. A step-by-step accession model was proposed to explain the observed pattern, taking into account the metabolic and adhesive properties of the different bacterial species.

In addition to revealing the spatial distributions of bacteria in mixed-species biofilms, biofilm fixation procedures allowed high-resolution imaging and segmentation of biofilms into individual cells. The ability to identify positions and orientations of cells in biofilms allowed researchers to use concepts and tools from colloidal physics to rationalize the observed cellular packing. The first work to exploit single-cell imaging in fixed biofilms used line-scanning confocal microscopy to study *Staphylococcus epidermidis* biofilms [148]. By tracking the centers of the spherical cells and analyzing the radial distribution function, biofilm compactness parameters were quantified. Surprisingly, the packing characteristics of cells in biofilms were found to vary dramatically even within a single sample, ranging from a disordered liquid phase to an open, fractal-like structure. More recently, using images of fixed samples obtained at different times during biofilm maturation, the architectural transitions undergone by cells in *V. cholerae* biofilms were revealed [149]. Specifically, in a mature biofilm cluster, vertical cells reside at the biofilm center and radially orientated cells are present at the periphery.

Fixed biofilm imaging strategies transformed the understanding of which cells are present and where each cell resides. However, fixed biofilms could not be used to study temporal changes in biofilm formation, preventing understanding of the full dynamical process of biofilm development from a single-cell to a 3D community. Recent improvements in confocal microscope design, availability of fluorescent proteins possessing increased photostability and quantum efficiency, and development of new computer algorithms that are particularly useful for resolving small objects (i.e., bacterial cells) made imaging of living biofilms with single-cell resolution possible. First, *V. cholerae* biofilm clusters were imaged as they grew from the founder cell to 10 000 cells [150]. The biofilm clusters transition from a two-dimensional (2D) branched morphology to a dense 3D cluster with a nematically ordered core (figure 24). Combining single-cell live imaging, mutagenesis, and agent-based computer simulations revealed the cellular ordering inside the biofilm to be the physical consequence of a competition between biofilm expansion and cell surface adhesion [151]. Specifically, during the initial 2D expansion phase, friction with the surface due to surface adhesion proteins impedes the expansion of the biofilm cluster. As a result, cells at the center of the cluster are under compressive force and transition from lying parallel to the surface to re-orienting perpendicular to the surface. Once verticalized, these cells send their progeny further into the third dimension, thereby creating a dome-shaped 3D biofilm cluster.

### 16.2. Current and future challenges

The ability to image individual live cells in 3D bacterial biofilms now makes possible the study of their behaviors. By measuring the levels of expression of specific genes using fluorescent reporters, questions that can now be addressed include whether cells in different regions of a biofilm produce distinct subsets or levels of quorum-sensing autoinducers, whether cells in close proximity to one another coordinate to build particular portions of the biofilm architecture, and whether persister cells that survive transient antibiotic exposure arise in specific locations in the biofilm. Moreover, single-cell imaging can be extended to polymicrobial biofilms to reveal the rich dynamics underpinning how different species compete or cooperate during biofilm development.

Several challenges need to be overcome to generate the next wave of information regarding spatiotemporal development of biofilms. For example, can we follow cell lineages inside biofilms? Can we resolve the shapes of individual biofilm-dwelling cells? Can we image biofilms in complex 3D environments similar to those found in nature? Here, we highlight some approaches that are being pursued to address these challenges.

- The time resolution of imaging will need to be increased to follow cell lineages in 3D biofilms. Lineage tracing has revolutionized our understanding of eukaryotic development. Indeed, the fate of each individual cell has been mapped in model organisms such as nematodes and zebrafish using lineage tracking [152]. Achieving lineage tracing for 3D biofilms is more challenging due to issues arising from the small sizes of bacterial cells along with phototoxicity and photobleaching, which currently limit the time between consecutive imaging frames to >10 min. Ideally, one would need at least 5–10 time-steps between each bacterial division event, which imposes an upper limit of 3–5 min between image acquisitions. Light sheet microscopy is poised to overcome this challenge due to the dramatically reduced phototoxicity and photobleaching of the technology [152]. The dual-view inverted selective plane illumination microscopy setup is particularly well suited for visualizing bacterial biofilm geometry [153, 156]. In addition to hardware improvements, new software developments are required to trace lineages in 3D biofilms, including improving segmentation accuracy and incorporating tracking algorithms similar to those developed for 2D bacterial colonies.
- Image analysis procedures need to be extended to bacteria with more complicated shapes than rods and spheres, for example, filamentous bacteria and spirochetes [154]. In the case of *V. cholerae*, which has been the focus of many of these single-cell analyses, the bacterium is a curved rod. However, current confocal optical resolution does not allow quantitative assessment of individual cell curvature inside of biofilms. Having algorithms that can extract detailed shape information for individual cells will allow researchers to ask questions such as: do bacteria change their shapes during biofilm maturation? What is the correlation between individual bacterial shape and the overall biofilm architecture? How does heterogeneity in bacterial shape affect cellular packing inside a biofilm?
- The current imaging setup (i.e., unconstrained biofilms on flat glass) is far from the environments in which biofilm-forming bacteria reside in nature or during infection. The geometries, stiffnesses, surface topographies, and surface chemistries of substrates all influence biofilm development. Thus, a key challenge will be to adapt imaging procedures such that complex surfaces with non-ideal optical properties are made suitable for high-resolution imaging. As an example, bacteria such as *B. subtilis* form biofilms in the 3D soil environment. Optically transparent particles mimicking soils (such as irregularly shaped glass beads) need to be developed and coupled with new imaging protocols that can handle the non-flat geometry of the growth substrate. Moving away from solid substrates, many clinically relevant biofilms, such as those made by *P. aeruginosa*, form while embedded in the mucus layer in the lungs of cystic fibrosis patients [155]. Mucus is complex with respect to thickness, chemical composition, and stiffness, and furthermore, these properties vary from patient to patient. Understanding how the heterogeneous mucus substrate as well as other in-host or in-environment milieu affect biofilm development will be required if the promise of new therapeutic or industrial approaches to chronic biofilm infections/biofouling/clogging is to be met.

### 16.3. Concluding remarks

The ability to visualize the location, orientation, shape, and progeny of individual cells in 3D biofilms has begun to define the key biophysical steps driving biofilm formation. Ultimately, we envision that single-cell imaging technology will become routine for biofilms. Together with genetic and biochemical perturbations, and the use of non-uniform substrates, we will gain a comprehensive understanding of how bacteria build their communities cell by cell.

# Research article

**Authors:** Yilin Wu, Haoran Xu

**Affiliations:** Department of Physics and Shenzhen Research Institute, The Chinese University of Hong Kong, Shatin, New Territories, Hong Kong, People’s Republic of China

## 17. Self-organized collective motion in bacterial communities

### 17.1. Status

Bacteria can move by various motility mechanisms, such as flagella rotation, pilus retraction, slime extrusion, or gliding with hidden engines. Motility is generally believed to be important for bacterial community development. During development of structured bacterial communities, motile and sessile subpopulations often coexist, and the motile subpopulation may be present as high-density groups. It is well known that self-propelled agents interacting with each other either physically or chemically can display rich patterns of self-organization in space and time. However, the potential forms of self-organized collective motion in bacterial communities as well as their roles in community development are not well understood.

Self-organized bacterial collective motion was first noticed and extensively studied in quasi-2D bacterial swarms. During swarming, cells move in densely packed groups and translocate across surfaces in a coordinated manner, forming transient clusters, vortices and streams. Swarming dynamics can be recapitulated by active matter models consisting of self-propelled particles that interact physically. Self-organization during swarming facilitates predation by the colony on microbial prey species and confers multi-drug tolerance to the colony [157]. The directed movement of cellular clusters and streams also enables long-range transport of non-motile cells of different bacterial species [158, 159]. This type of cargo transport may help to establish mutualistic relations during range expansion and to shape the spatial structure of polymicrobial communities such as those found in the human microbiome [158, 159].

Collective motion is much less studied in heterogeneous bacterial communities where motile cells coexist with other phenotypes. Recently it was found that the motile subpopulation in sessile colonies of flagellated bacteria can self-organize into two adjacent motile rings surrounding the colony [160] (figure 25(a)). Cells in the outer ring move unidirectionally in a clockwise manner with high polar order, while the inner ring is nematically ordered. This unique form of self-organization arises from steric and hydrodynamic interactions between swimming cells. It rectifies fluid flows due to flagellar rotation at the single-cell level in a way similar to what cells do at the edge of swarms, thereby driving stable colony-scale fluid transport at a speed of ~30 *μ*m s^−1^ (figure 25(b)). This mechanism of long-range active transport may have a profound effect on the physiology of bacterial communities in heterogeneous environments by redistributing nutrients and chemical signals.

In addition to spatial self-organization, populations of densely packed bacteria can synchronize in time and display collective oscillation. Collective oscillatory behavior is ubiquitous in nature and it plays a vital role in many biological processes. Collective oscillation in multicellular systems often arises from physicochemical coupling (e.g. via diffusive chemical signals) between individual cells that act as local oscillators. By contrast, we recently reported a new form of biological collective motion that does not require individual cells to have any oscillatory degree of freedom [161]. In this phenomenon, individual cells in a bacterial swarm move in an erratic manner, but highly robust collective oscillatory motion emerges after averaging the motion of a large number of cells (figures 25(c) and (d)). This weak synchronization phenomenon results from spontaneous symmetry breaking mediated by purely local interactions between individual cells. Oscillatory flows associated with the collective oscillation may influence the spatial distribution of sessile cells in the swarm and eventually affect the order of biofilm patterning [161].

### 17.2. Current and future challenges

First of all, potential forms of self-organized collective motion in structured bacterial communities are largely unexplored. The difficulty to acquire large-scale data for characterizing the physiological state, trajectory, and fate of different subpopulations throughout the development of a bacterial community presents a major hurdle. Probing the physiology and behavior of cells in structured communities would require technological advances in quantitative multidimensional and multispectral microscopy. Another challenge is the lack of knowledge about the *in situ* physicochemical microenvironment within structured bacterial communities. Such knowledge is essential for evaluating the interaction between motile cells as well as the interaction between cells and their microenvironment, which is in turn crucial to understanding the mechanisms underlying any emergent collective behavior. While advances in non-equilibrium physics, especially in active matter theory, are providing an increasing number of novel predictions, the predictions would offer more useful guidance if cell–cell and cell–environment interactions in bacterial communities could be better understood experimentally with the development of reporter and noninvasive sensor techniques.

Second, the roles of self-organized collective motion in bacterial community development are still elusive. Upon discovering new forms of self-organized collective behavior in bacterial communities, it is often easier to understand the underlying mechanisms than to understand their roles in community development, because the development of bacterial communities is a dynamic process occurring over a time scale usually much longer than the self-organized collective behavior would last. To understand the roles, we could perturb the collective behavior by manipulating single-cell motion, cell–cell interaction, or cell–environment interaction, and see how the developmental process would be affected by these perturbations.

### 17.3. Advances in science and technology to meet challenges

Advances in non-equilibrium physics may generate novel conceptual ideas and experimentally testable models on self-organization in general active matter systems. Such advances will help to understand the mechanisms of bacterial self-organization, and will provide useful guidance to discover new forms of self-organized collective motion in structured bacterial communities. For example, motility-induced phase separation proposed in active matter systems [162] has been used to explain fruiting body development in biofilms of the gliding bacteria *M. xanthus* [163]; depletion-induced phase separation that causes aggregation of motile cells in liquid suspensions [164] could give rise to novel forms of collective motion in structured bacterial communities.

Efforts to discover collective motion in structured bacterial communities will benefit from advances in microscopy principles and imaging techniques, such as light-sheet microscopy and novel fluorescence labeling methods. The advances will enable acquisition of large-scale data on the physiology and behavior of cells throughout the development of a heterogeneous bacterial community. Such information could also fuel data-driven agent-based modeling of collective motion in structured bacterial communities [165]; this approach may overcome the limitation of incomplete knowledge of cell–cell and cell–environment interactions, while obtaining essential insights into the phenomenon.

As suggested above, in order to understand the roles of self-organized collective motion in bacterial community development, it may be useful to perturb the collective behavior by manipulating the motion and interaction patterns of cells. This would require noninvasive and *in situ* control of physicochemical microenvironments and cellular behavior in the bacterial community. Advances in microfluidics, synthetic biology, optogenetics, and biomaterials engineering would make this goal possible. For instance, optogenetic control of type IV pilus motility and c-di-GMP synthesis in *P. aeruginosa* enables manipulation of spatial organization of cells during biofilm development [96].

### 17.4. Concluding remarks

Here we introduced several remarkable forms of self-organized collective motion in bacterial communities and discussed their implications in material transport and biofilm development. Advances in fields such as microscopy imaging, microfluidics and synthetic biology will boost the discovery of new forms of collective motion in structured bacterial communities and will help to elucidate their biological functions. We also envision that advances in non-equilibrium physics may generate conceptual ideas and experimentally testable models that will provide useful guidance to discover new forms of bacterial collective motion.

# Research article

**Authors:** Knut Drescher, Jörn Dunkel

**Affiliations:** 1 Max Planck Institute for Terrestrial Microbiology, 35043 Marburg, Germany; 2 Department of Physics, Philipps-Universität Marburg, 35043 Marburg, Germany; 3 Department of Mathematics, Massachusetts Institute of Technology, Cambridge, Massachusetts, MA 02139–4307, United States of America

## 18. Learning principles of bacterial biofilm dynamics from the behavior of single cells

### 18.1. Status

At first glance, what microbiologists and physicists refer to as a ‘model’ of a process or a system appears to be very different: whereas physicists think of equations, microbiologists typically think of a summary of the key processes depicted in the form of a schematic diagram [166]. Yet, despite being formulated in different languages, these two complementary types of models have in common that they summarize and conceptualize hypotheses about the relevant processes and mechanisms of a given system. The successful integration of biological and physical models holds the key for understanding bacterial biofilm dynamics, as their inherent abstraction may reveal generic optimization principles that govern growth, evolution, and stress responses in these multicellular communities. The identification of overarching dynamical principles, analogous to the Darwinian principle of evolution or the principle of least action in physics, promises a unified understanding of biofilm dynamics across different species and environmental conditions. Moreover, when put into mathematical form, such principles can serve as a starting point for formulating quantitative models with predictive power for particular species and environments.

For bacterial biofilm dynamics on evolutionary time scales, several principles for the effects of social interactions on selection in the spatial context of biofilms have already been identified [167]. These concepts have enabled accurate and predictive models for communities with low species complexity. In contrast, multi-species spatial evolutionary dynamics remain poorly understood.

For biofilm dynamics on the multi-generational time scales of community growth, several important principles have also been identified. These include the concept that during biofilm growth, concentration gradients are generated by the community via the consumption and release of diffusible molecules, resulting in spatially-organized heterogeneous behaviors and growth rates on length scales that are significantly larger than the size of a single cell [168]. Another generic principle underlying biofilm community growth is that the multicellular architecture development of small biofilms is primarily driven by mechanical interactions between cells and the cellular growth dynamics [169, 170]. At the molecular level, commonalities exist between different species and growth environments in terms of the regulatory principles underlying surface adhesion, matrix production, and dispersal. Despite such major progress, however, we are not yet able to accurately predict biofilm growth and architecture dynamics for previously uncharacterized species and environments.

For dynamical processes on intra-generational time scales in biofilms, such as the response of biofilm communities to abiotic and biotic stresses, broadly applicable principles have yet to be discovered [171].

The usefulness of fundamental principles for enabling quantitative predictions of multicellular growth phenomena depends on the level of their abstraction (or ‘coarse-graining’). For example, at some very abstract level, all developmental processes in biofilms appear unified by the fact that they occur in a living system that is driven out of thermodynamic equilibrium by metabolism. Although certainly true, this ‘non-equilibrium principle’ is unlikely to lead to a predictive model of biofilm growth dynamics. On the other end of the abstraction spectrum, the biochemical principles for amino acid binding also hold limited predictive power for describing the biofilm architecture development at the intercellular level. Identifying the appropriate level of abstraction to capture the essential mechanisms underlying biofilm development therefore remains a key challenge.

### 18.2. Current and future challenges

The limited number of viable principles for understanding and predicting the multiscale dynamics of bacterial biofilms calls for new integrated experimental and theoretical approaches that can bridge the relevant range of length and time scales. Such efforts should be guided by the goal to establish a conceptual framework that will allow us to predict macroscale community growth and morphology on the basis of intracellular gene expression, local cell interactions and fast stress response mechanisms. A promising starting point can be the hypothesis that, on biofilm microcolony growth time scales, mutational effects are less relevant, whereas intracellular dynamics and cellular interactions are of central importance. If we accept this premise, then detailed data capturing the behavior of single cells in their multicellular context within biofilm microcolonies will be essential for successful model development.

### 18.3. Advances in science and technology to meet challenges

Recent advances in microscopy and image analysis now allow the 3D tracking of all individual cells during biofilm growth up to a few thousand cells with high accuracy (figure 26). In addition, it is possible to simultaneously quantify fluorescent reporters within each cell and reconstruct cellular lineage relationships in space and time [169, 170], using specific software for biofilm image analysis [172]. Translating these unprecedented high-dimensional data into a qualitative or mathematical model poses a formidable challenge. Traditionally, scientific progress has relied heavily on the human ability to recognize patterns in sufficiently low-dimensional data. Combined with hypotheses about relevant mechanisms, these patterns informed the formulation of biological and physical models—irrespective of whether the systems of interest were atoms, molecules, cells, or planets. However, over the last few years, there has been significant progress in data science that may lead to a paradigm shift in how models are formulated in all fields of science. Embracing these new data-driven paths toward model development, and combining them with existing knowledge and concepts, promises a major leap in our ability to discover the key mechanisms, processes, and ultimately principles that govern dynamical processes in biofilms.

How can we successfully integrate data-driven model development to understand biofilm growth dynamics? This challenge can be divided into three interwoven sub-problems: data representation, data compression (dimensionality reduction), and model inference. State-of-the-art experiments provide nearly complete time-resolved positional and orientational information as well as internal state parameters for each individual cell [169, 170]. Given the volume and complexity of these data, a traditional physics-based approach is to construct coarse-grained order-parameter fields by averaging selected variables over local cell-neighborhoods. By adopting such a continuum description, one could then try to learn effective continuum models directly from data using recently developed learning techniques for partial differential equations (PDEs) [173]. For this approach to be feasible, prior knowledge regarding physical symmetries and conservation laws is essential to constrain the model search space. Although definitely worth pursuing, PDE-based continuum descriptions of early-stage biofilm development may face fundamental limitations. In contrast to classical physical systems, which contain macroscopically large particle numbers within the coarse graining volume, the typical averaging volumes in biofilms only contain a relatively small number of cells (often ≪ 100). This means that local-order parameter estimates can have large variance and may become very sensitive to changes in the coarse-graining volume. As a consequence, measurements of field derivatives, which are needed as input for PDE-learning algorithms, can be corrupted by noise, and both structure and parameters of learned dynamical models can vary depending on the coarse-graining volume.

An alternative and potentially more robust approach, which makes explicit use of the single-cell resolution data, is agent-based modeling. In this case, the key challenge is to infer the effective cell–cell interaction processes as functions of the internal genetic and metabolic cell-parameters, the spatiotemporal extracellular matrix composition, and the externally applied biochemical and physical stimuli. A promising path toward inferring the underling dynamical equations is through sparse identification of nonlinear dynamical systems [174]. Moreover, GPU-based simulation techniques make it possible to perform large parameter scans for experimentally relevant biofilm sizes [169], so that explicit agent-based modeling is computationally competitive relative to coarse-grained approaches.

Finally, we mention another approach, which may enable major conceptual advances, but thus far has not been exploited in the context of biofilms. Single-cell data enables spatiotemporal network representations of biofilm architecture that can be efficiently combined with optimal transport ideas [28] to yield a statistical topological characterization of biofilm growth dynamics. Optimal transport theory studies how probability distributions can be evolved while minimizing one or more cost constraints. This framework has found successful applications in a wide range of areas, including image retrieval, machine learning and inverse problems, but it is not yet widely used for describing multicellular dynamics. The combination of optimal transport concepts with traditional modeling approaches seems particularly promising for identifying general principles governing biofilm dynamics. If one were able to reconstruct the approximate cost function(s) that are optimized as biofilms grow, which should be feasible with current experimental and computational techniques, then this would bring us one step closer to understanding biofilm dynamics on a more principled basis, similar to the optimization principles governing classical physical systems.

### 18.4. Concluding remarks

The recent technological advances in microscopy and image analysis that enable the characterization of all individual cells in their multicellular context during early biofilm development could provide the basis for the data-driven learning of models with a tunable level of abstraction. These approaches provide a direct path from data to quantitative models and hypotheses for relevant processes and mechanisms in biofilm dynamics. Due to the varying degree of abstraction that these models promise, these data-driven approaches may ultimately lead to the identification of dynamical optimization principles. These concepts and techniques could provide a major step forward in our understanding of multicellular microbiology, which may be just around the corner.
